# Antiviral responses induced by Tdap-IPV vaccination are associated with persistent humoral immunity to *Bordetella pertussis*

**DOI:** 10.1038/s41467-024-46560-w

**Published:** 2024-03-08

**Authors:** Joshua Gillard, Madeleine Suffiotti, Peter Brazda, Prashanna B. Venkatasubramanian, Pauline Versteegen, Marien I. de Jonge, Dominic Kelly, Sagida Bibi, Marta Valente Pinto, Elles Simonetti, Mihaela Babiceanu, Andrew Kettring, Cristina Teodosio, Ronald de Groot, Guy Berbers, Hendrik G. Stunnenberg, Brian Schanen, Craig Fenwick, Martijn A. Huynen, Dimitri A. Diavatopoulos

**Affiliations:** 1 Laboratory of Medical Immunology, Radboud Institute for Molecular Life Sciences, Radboud University Medical Center, Nijmegen, The Netherlands; 2 Radboud Center for Infectious Diseases, Radboud University Medical Center, Nijmegen, The Netherlands; 3 Department of Medical BioSciences, Radboud University Medical Center, Nijmegen, The Netherlands; 4 Department of Anesthesiology, Perioperative and Pain Medicine, Stanford University School of Medicine, Stanford, CA USA; 5 Service of Immunology and Allergy, Lausanne University Hospital, University of Lausanne, Lausanne, Switzerland; 6 Princess Máxima Center for Pediatric Oncology, Utrecht, The Netherlands; 7 Center for Translational Immunology, University Medical Center Utrecht, Utrecht University, Utrecht, The Netherlands; 8 Centre for Infectious Disease Control, National Institute for Public Health and the Environment, Bilthoven, The Netherlands; 9 Department of Paediatrics, Oxford Vaccine Group, University of Oxford, Oxford, UK; 10 Oxford NIHR Biomedical Research Centre, Oxford University Hospitals NHS Foundation Trust, Oxford, UK; 11 Egas Moniz Center for Interdisciplinary Research (CiiEM), Egas Moniz School of Health & Science, Caparica, Almada Portugal; 12 Sanofi Pasteur VaxDesign, Orlando, FL USA; 13 Leiden University Medical Center, Immunohematology & Blood Transfusion, Leiden, The Netherlands

**Keywords:** Inactivated vaccines, Innate immune cells, Inactivated vaccines, Infection

## Abstract

Many countries continue to experience pertussis epidemics despite widespread vaccination. Waning protection after booster vaccination has highlighted the need for a better understanding of the immunological factors that promote durable protection. Here we apply systems vaccinology to investigate antibody responses in adolescents in the Netherlands (N = 14; NL) and the United Kingdom (N = 12; UK) receiving a tetanus-diphtheria-acellular pertussis-inactivated poliovirus (Tdap-IPV) vaccine. We report that early antiviral and interferon gene expression signatures in blood correlate to persistence of pertussis-specific antibody responses. Single-cell analyses of the innate response identified monocytes and myeloid dendritic cells (MoDC) as principal responders that upregulate antiviral gene expression and type-I interferon cytokine production. With public data, we show that Tdap vaccination stimulates significantly lower antiviral/type-I interferon responses than Tdap-IPV, suggesting that IPV may promote antiviral gene expression. Subsequent in vitro stimulation experiments demonstrate TLR-dependent, IPV-specific activation of the pro-inflammatory p38 MAP kinase pathway in MoDCs. Together, our data provide insights into the molecular host response to pertussis booster vaccination and demonstrate that IPV enhances innate immune activity associated with persistent, pertussis-specific antibody responses.

## Abstract (web summary)

Epidemics of whooping cough caused by *Bordetella pertussis* have been seen and are linked to waning immunity globally. Here the authors explore responses to inactivated poliovirus (IPV) in the Tdap-IPV vaccine and show it stimulates early antiviral responses in monocytes and dendritic cells that are associated with long-lived pertussis antibody responses.

## Introduction

Pertussis is a highly transmissible respiratory disease that is caused by the bacterium *Bordetella pertussis*^1^ and has re-emerged as a serious public health issue despite high vaccination coverage in many countries^2^. Apart from primary vaccination of infants, booster vaccination with acellular pertussis (aP) vaccines is widely used to augment protection against pertussis in preschool children, adolescents, and adults including pregnant women^3^. Pertussis booster vaccines are formulated with tetanus and diphtheria toxoid (Tdap), either with or without inactivated poliovirus (IPV) (Tdap-IPV)^4^ and induce pertussis-specific memory T and B cells^5,6^ and IgG antibodies against pertussis antigens^7^. Although vaccine-induced serum antibody levels have been associated with clinical protection against pertussis in a household exposure setting^8^ and in vaccine efficacy trials^9,10^, both serum antibody levels and clinical protection induced by aP vaccines decrease rapidly after immunization^11,12^. The waning immunity that is associated with aP vaccines is believed to be at least partially responsible for the re-emergence of pertussis in vaccinated populations^13^, highlighting the need for new vaccination strategies and an improved understanding of the mechanisms of persistent vaccine-induced immunity.

The quality and magnitude of the adaptive immune response to vaccination is largely determined by the interactions of vaccine components with the innate immune system^14^ and it has thus become increasingly important to identify the cellular and molecular mechanisms by which these components are sensed. Systems biology approaches that quantify early immune processes have led to the identification of innate immune molecular signatures predicting humoral immunity up to 1 year after vaccination. Furthermore, these studies have highlighted that vaccines directed against different infectious agents can elicit different transcriptional and cellular responses soon after vaccination^15–22^. Currently, innate drivers of adaptive immune responses against pertussis have not yet been identified in humans. A systems biological analysis of aP-containing vaccines may therefore provide insights into how durable humoral immune responses are generated against different vaccine antigens and the mechanisms by which such vaccines are sensed by the innate immune system.

Since adolescents have a very high pertussis disease burden and represent a core transmission group^23–25^, we conducted an international, multicenter vaccination study in this age group to investigate early signatures of persistent humoral responses to Tdap-IPV vaccination. Serum antibody responses to Tdap-IPV vaccine antigens were quantified up to 1 year after vaccination. Gene expression analysis of whole blood early after vaccination highlighted innate immune gene expression pathways that are correlated with antibody responses. To identify the cell subsets and signaling pathways underlying this transcriptional signature, we performed mass cytometry and single-cell RNA sequencing of circulating APCs. We examined the response to Tdap-IPV vaccination at multiple levels, including changes in circulating cell abundance, cytokine production, gene expression, and phospho-signaling responses. Finally, publicly available transcriptomic data from Tdap vaccinated individuals^26^ were analyzed in order to compare responses with those induced by Tdap-IPV. Our study identifies early correlates of persistent humoral responses, provides insights into the molecular host immune response to aP vaccines, and identifies a previously unrecognized role for IPV.

## Results

### Study design and antibody responses to Tdap-IPV vaccination

**Fig. 1:**
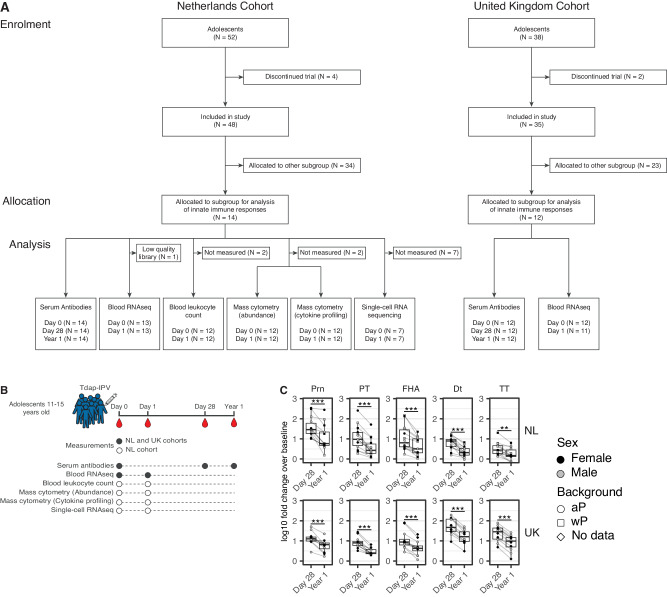
Flow diagram, study procedures, and humoral responses to Tdap-IPV vaccination. In a phase IV multi-center clinical study conducted in the Netherlands (NL) and United Kingdom (UK), male and female adolescent participants between 11–15 years old who were primed in infancy with either an acellular (aP) or a whole-cell pertussis (wP) vaccine received a booster dose of tetanus-diphtheria-acellular pertussis-inactivated polio (Tdap-IPV). *N* = 14 participants (NL cohort) and *N* = 12 participants (UK cohort) were allocated for analysis of early post-vaccination immune responses. **A** Flow diagram of participants who were enrolled in the current study and analyzed. **B** Study design indicating blood sampling and analyses performed at each timepoint. Closed circles indicate measurements performed in both cohorts, and open circles indicate those performed only in the NL cohort. **C** Antibody response (log10 fold change of IgG concentrations vs baseline) for the specified Tdap antigens at 28 days and 1 year post-vaccination in the NL and UK cohorts. Data are *N* = 14 for the NL cohort and *N* = 12 in the UK cohort and are represented as box plots, with bounds from 25th to 75th percentile, median line, and whiskers, which extend to the largest or smallest value no further than 1.5 * the inter-quartile range. Statistical significance was determined using a two-sided paired Wilcoxon test, ** *p* < 0.01, *** *p* < 0.001. Nominal *p* values are reported. NL cohort: PT, *p* = 0.00012; FHA, 0.00012; Prn, *p* = 0.00012; Dt, *p* = 0.00012; TT, *p* = 0.0067. UK cohort: PT, *p* = 0.00049; FHA, 0.00049; Prn, *p* = 0.00049; Dt, *p* = 0.00049; TT, *p* = 0.00049. Abbreviations: FHA filamentous hemagglutinin, Prn pertactin, PT pertussis toxin, Dt diphtheria toxoid, TT tetanus toxin, F female, M male. Source data are provided in the Source Data file.

As part of a large international multicenter phase IV clinical trial to investigate the immune response to Tdap-IPV vaccination^7^, we performed an immunological substudy in two cohorts of male and female adolescents aged 11–15 years from the Netherlands (NL, *N* = 14) and the United Kingdom (UK, *N* = 12) (Fig. 1A). NL participants in this age range have either an aP or a whole-cell pertussis (wP) vaccine priming background, whereas participants in the UK cohort were all primed with aP vaccines. All participants received an aP preschool booster vaccination at 3–4 years of age. Blood samples were collected from all participants before vaccination (D0), as well as 1 day (D1), 28 days (D28), and 1 year (Y1) post-vaccination for various immunological analyses (Fig. 1B, Table S1). The early peripheral response to Tdap-IPV vaccination was examined at D1, which corresponds to the peak of the innate response following vaccination with aP vaccines^26,27^, and innate immune activity at this timepoint has been correlated with humoral responses for other vaccines^16,20,21^. Antibody responses were measured in serum at D0, D28 and Y1 post-vaccination. Using a multiplex immunoassay^28,29^, we measured IgG antibody concentrations against pertussis toxin (PT), filamentous haemagglutinin (FHA), pertactin (Prn), as well as diphtheria toxoid (Dt), tetanus toxin (TT), and the three poliovirus serotypes (Polio.I Polio.II and Polio.III). Antibody concentrations increased 28 days following Tdap-IPV immunization, and decreased by 1-year post-vaccination, such that the levels approached the baseline concentrations for some participants and antigens (Fig. S1)^7^. Next, we calculated the magnitude of the antibody response as the log10 fold change (LFC) over baseline. Antibody responses as measured by LFC were significantly higher at day 28 compared to 1-year post-vaccination (Fig. 1C). Of the five Tdap antigens, antibody responses to Prn were the strongest at both D28 and Y1 in the NL cohort (Fig. S2A, B), as has also been observed in other age groups^7^. Conversely, Dt and TT antibody responses were highest at both D28 and Y1 in the UK cohort (Fig. S2C, D). Of note, pre-vaccination Dt and TT antibody levels were significantly higher in the UK cohort than those in the NL cohort (Fig. S3A). We also observed higher baseline levels of pertussis-specific memory B cells in PBMCs of the UK cohort participants (Fig. S3B), a variable we previously reported as a biomarker of vaccine responsiveness^6^. Participants in the NL cohort were older than those in the UK cohort (Fig. S3C) and differed in the proportion of participants with aP or wP priming backgrounds (Table S1). The magnitude of the antibody response varied considerably between participants, with >20-fold difference in responses for some antigens and time points (Fig. 1C). Furthermore, dispersion of antibody responses, as measured by the coefficient of variation (CV), was highest for 1-year antibody responses in both NL and UK cohorts (Table S2). CV values of antibody responses at this timepoint were higher in the NL cohort (CV range between antigens = 80–124%) than the UK cohort (CV range = 29–55%). In our previous report, no effect of sex or priming background (aP vs wP vaccines) on antibody responses was observed^7^ and within our substudy cohort, neither sex nor vaccination background explained variation in the Tdap antibody response after 1 year in either cohort (Table S2). This raised the question whether we could identify early cellular and/or molecular events that correlate with antibody responses and potentially serve as early biomarkers for long-lived humoral immunity against pertussis.

### Tdap-IPV vaccination induces innate immune activation and antiviral responses

**Fig. 2:**
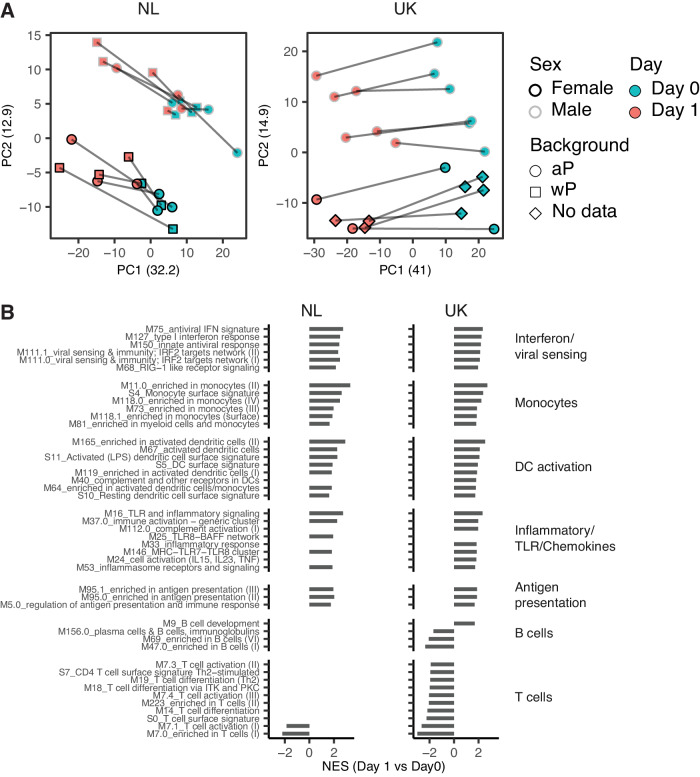
The early immune response to Tdap-IPV vaccination is characterized by innate immune activation and antiviral responses. **A** Principal component analysis of whole blood RNA sequencing data for participants in the Netherlands (NL, left panel) or United Kingdom (UK, right panel) cohorts. Sample timepoint, gender, and vaccination background are shown, as well as the portion of variance explained by each component. Lines connect a participant’s pre (D0) and 1 day post (D1) vaccination samples. Abbreviations: F female, M male, aP acellular pertussis vaccine, wP whole-cell pertussis vaccine. **B** Blood transcription modules (BTMs) enriched (FDR < 0.05) 1 day after Tdap-IPV vaccination (continued on fig. S5) in the NL (left panel) and UK (right panel) cohorts. Gene set enrichment analysis (GSEA) was used to calculate the normalized enrichment score (NES) of BTMs using a gene list ranked by the log2-fold change of gene expression over baseline (D1/D0). Statistical significance and *p*-values were calculated against an empirical null distribution and reflect two-sided tests. False discovery rate (FDR) adjusted *p* values were calculated; enriched BTMs (FDR < 0.05) are grouped based on their biological function. Data are 26 paired samples from *N* = 13 participants in the NL cohort, and 22 paired samples from *N* = 11 participants in the UK cohort. Source data are provided in the Source Data file.

To examine the early immune response to Tdap-IPV vaccination, we quantified gene expression signatures in blood. Principal component (PC) analysis at D0 and D1 showed strong vaccination effects on the first PC, whereas the transcriptomes of male and female participants separated on the second PC, reflecting a gene expression pattern in blood between the sexes^30^ (Fig. 2A). To derive biological insights from differences in the transcriptomes, we analyzed differential gene expression. Comparison of D0 vs D1 responses between sexes (NL cohort, *n* = 7 males and *n* = 6 females; UK cohort, *n* = 6 females and *n* = 6 males, Fig. S4A, B) and priming backgrounds (NL cohort, *n* = 6, aP-primed; *n* = 7, wP-primed, Fig. S4C) revealed few, if any differentially expressed genes. Therefore, based on the similarity of antibody and transcriptional responses, participants were not separated by gender or priming background in subsequent analyses. We identified 559 differentially expressed genes (DEGs, FDR < 0.05, −0.5 < LFC > 0.5) in the NL cohort and 1709 DEGs in the UK cohort (Fig. S4D, E). We performed gene set enrichment analysis (GSEA^31^) using blood transcription modules (BTMs^17^) to identify pathways that were altered by Tdap-IPV vaccination (Fig. 2B). Response patterns in the NL and UK cohorts were generally very similar. For instance, BTMs for monocytes, antigen presentation, dendritic cell (DC) activation, and inflammatory responses were highly enriched following vaccination in both the NL and UK cohorts. Strikingly, antiviral immunity and interferon response BTMs were among the most strongly enriched in both cohorts. Furthermore, both cohorts exhibited enhanced neutrophil gene expression and decreased natural killer (NK) cell gene expression after 1 day (Fig. S5). Of note, our analysis also revealed markedly different gene expression signatures between the cohorts for specific BTMs, where the UK cohort displayed significant enrichment of BTMs for plasma cells and B cells (*M156.0 plasma cells & B cells, immunoglobulins; M47.0*, *M69 enriched in B cells (I and VI)*), as well as multiple BTMs for T-cell activation and differentiation (Fig. 2B).

### Early gene signatures correlate with the antibody response to Tdap-IPV

**Fig. 3:**
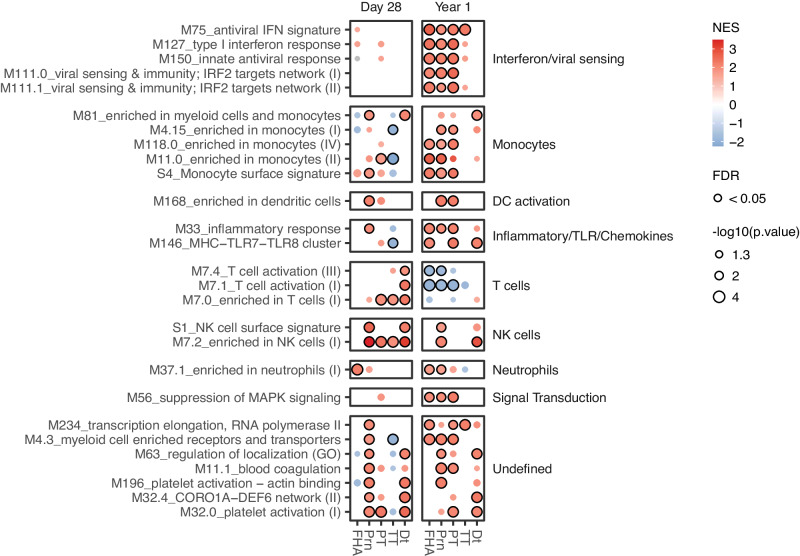
Molecular signatures associated with the adjusted log-fold change antibody response induced by Tdap-IPV. Dot plot of blood transcription modules (BTMs, rows) whose activity 1 day post-vaccination (Day 1/Day 0) is associated with adjusted log10-fold change (Methods) of Tdap-IPV induced antigen-specific antibody responses (columns) 28 days (Day 28/Day 0) or 1 year (Year 1/Day 0) post-vaccination. Data are shown for the combined participants of the Netherlands (NL, 26 paired samples from *N* = 13 participants) and United Kingdom (UK, 22 paired samples from *N* = 11 participants) cohorts. Gene set enrichment analysis was used to identify positive (red) or negative (blue) enrichment of BTMs within pre-ranked gene lists, where genes were ordered according to their correlation between gene expression and antibody response. Statistical significance and p-values were calculated against an empirical null distribution and reflect two-sided tests. False discovery rate (FDR) adjusted *p* values were calculated. BTMs shown, (nominal *p* value < 0.05) have more than two FDR adjusted significant associations (FDR < 0.05) with antibody responses, and enriched BTMs with FDR < 0.05 are highlighted with a black border. BTMs are annotated according to biological function on the right. Abbreviations: NES normalized enrichment score, FHA filamentous hemagglutinin, PRN pertactin, PT pertussis toxin, Dt diphtheria toxoid, TT tetanus toxin. Source data are provided in the Source Data file.

We aimed to determine whether changes in the gene expression of BTMs post-vaccination (Fig. 2B) correlated with variations in the magnitude of the humoral response between individuals (Fig. 1C). Considering that baseline antibody levels were highly correlated with antibody responses, as has been previously described for other vaccines^32,33^ (Fig. S6), we accounted for the cross-correlation of antibody LFC with baseline antibody levels by calculating the adjusted log-fold change (adjLFC, Methods) for each response. Thereafter, the adjLFC at D28 and Y1 post-vaccination was correlated with the D1/D0 log2-fold changes for each gene in the transcriptomics dataset. We then applied GSEA using BTMs to the resulting gene list ranked by the correlation with the antibody response. We first analyzed correlations per cohort, revealing both similarities and discrepancies between cohorts in the early molecular signatures of antibody responses, particularly B-cell responses that were positively associated with the induction of antibodies in the UK cohort, as well as high correlations of innate immune BTMs in the NL cohort (Fig. S7). Analysis of D28 IPV antibody responses in the NL cohort furthermore revealed molecular signatures of poliovirus serotypes (Fig. S8). To enhance statistical power and uncover the shared molecular signature of Tdap-IV vaccination in the NL and UK cohorts, we normalized antibody and gene expression responses per cohort (Methods) and calculated correlations and BTM enrichment across all pooled participants. We observed correlations of BTMs for interferon and antiviral sensing, monocytes, DC activation, and inflammatory responses with pertussis-specific PT, Prn, and FHA antibody responses at 1 year post-vaccination (Fig. 3). Similarly, following ranking correlations of BTMs with pertussis antigens by normalized enrichment score (NES), we observed signatures primarily related to monocytes, DCs, and antiviral immunity (Fig. S9A), of which the M*75 antiviral IFN signature* BTMs had one of the highest correlations with 1-year FHA antibody responses. Leading edge analysis of genes in this BTM revealed the virus-response genes *IRF7*, *IFIT1*, *RSAD2*, *HERC5*, *OAS3*, and *DDX60*, which were overall positively correlated with the FHA antibody response in both cohorts (Fig. S9B). Considering that BTMs largely overlapped between Y1 pertussis responses (Fig. 3), we ranked the top correlating genes according to the mean correlation with PT, FHA, and Prn responses in order to identify common gene-level biomarkers of vaccination (Fig. S9C). Individual genes belonging to BTMs associated with antiviral responses, interferon, monocytes, antigen presentation, and DC activation were positively correlated with antibody responses. In this context, major histocompatibility complex (MHC) class II genes HLA-A and HLA-DMA, which are primarily expressed by professional APCs such as monocytes and DCs, were highly correlated with antibody responses. In addition, we observed positive correlations with *IRF7* and interferon-stimulated genes (ISGs) *ISG15*, *IFI30*, *IFIT1*, *RSAD2*, *HERC5*, and *IFI6*. Overall, our results demonstrate that innate immune activation, including antiviral responses, 1 day post-vaccination is associated with antibody responses up to 1-year post-vaccination with Tdap-IPV.

### Tdap-IPV vaccination induces type-I interferon and pro-inflammatory cytokine production in monocytes and dendritic cells

Whole blood gene expression analysis identified a clear vaccination-induced innate immune signature in both cohorts that was associated with antigen presenting cells (APCs) such as monocytes and DCs. Furthermore, we observed strong correlations of BTMs for monocyte and DC activation with the Y1 antibody response to multiple vaccine antigens. We used mass cytometry to examine whether these findings could be explained by changes in cellular abundance and/or activation, using a panel designed to capture cellular heterogeneity and functional states (Table S3). Whole blood was collected from participants in the NL cohort at baseline and 1 day after vaccination and was incubated in vitro for a short period without further stimulation in the presence of brefeldin A, followed by intracellular cytokine staining and acquisition on a mass cytometer. Cells were manually gated based on phenotypic surface marker expression (Fig. S10A), which identified 12 populations of innate and adaptive immune cells (Fig. S10B, C).

**Fig. 4:**
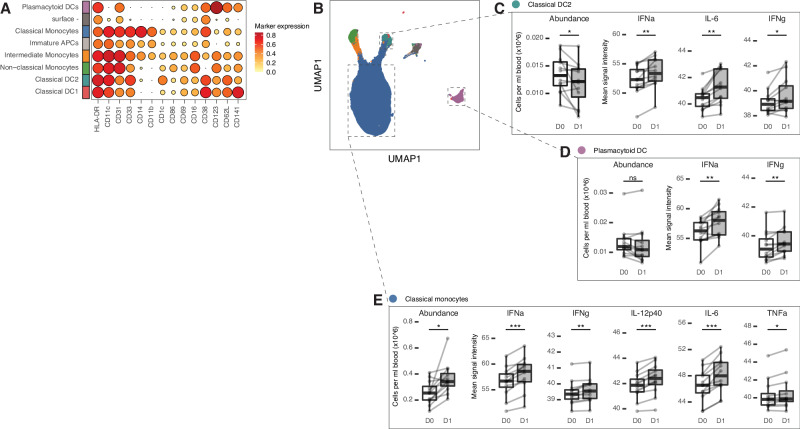
Tdap-IPV vaccination induces interferon and inflammatory cytokine production in classical monocytes, plasmacytoid and classical DC2 cells. Eight subpopulations of antigen presenting cells were identified across baseline (D0) and Day 1 (D1) blood samples after clustering and manual merging of similar clusters (Supplementary Information, Fig. S11). **A** Dot plot of normalized phenotypic protein marker expression values for each of the subpopulations indicated on the left margin. **B** UMAP visualization of all subpopulations with vaccine-responding subpopulations indicated with a dashed frame. Changes in abundance (box plots displaying the number of cells in blood) and cytokine production (box plots displaying the mean signal intensity) are shown for (**C**) classical DC2 cells, (**D**) plasmacytoid DCs, and (**E**) classical monocytes. Data are 24 samples from *N* = 12 participants in the Netherlands cohort, data points for (**C**), (**D**), and (**E**) represent values for each sample and those from the same participant are joined by a gray line. Box plots display bounds from 25th to 75th percentile, median line, and whiskers, which extend to the largest or smallest value no further than 1.5 * the inter-quartile range. Box plots with white fill correspond to D0 samples, and those with gray fill correspond to D1 samples. Statistical significance (false-discovery rate (FDR) adjusted two-sided p.value) was calculated from a mixed-effects regression model for each response (abundance or cytokine) with study day and participant number specified as fixed and random effects, respectively * FDR < 0.05; ** FDR < 0.01; *** FDR < 0.001, ns FDR > 0.05. Source data are provided in the Source Data file.

One day after vaccination, the number of total circulating leukocytes was significantly increased in blood (Fig. S10D). T-cells were significantly lower 1 day after vaccination (Fig. S10E), which may explain the decreases in the expression of T-cell BTMs (Fig. 2B). We also observed significant increases in granulocytes and monocytes. Next, we conducted a detailed subpopulation analysis of the APC compartment, based on surface marker expression (Methods, Fig. S11). Altogether, eight populations of APCs were defined, including classical monocytes (cMo), intermediate monocytes (iMo), non-classical monocytes (ncMo), pDCs, classical CD141+ and classical CD1c+ dendritic cells (DC1 and DC2, respectively), as well as apparently immature CD19- HLA-DR+ immune cells that expressed CD11b and CD11c (Immature APCs), and a population that almost completely lacked all phenotypic marker expression (surface-, Fig. 4A, B). For each of these subpopulations, changes in abundance and cytokine production were calculated (D0 vs D1). In total, four cell subpopulations displayed elevated inflammatory cytokine production, two of which were also differentially abundant post-vaccination (Table S4). Circulating DC2 cells decreased in the blood post-vaccination but upregulated the expression of IFN-a and IFN-g, as well as IL-6 (Fig. 4C). Similarly, pDCs also displayed significantly increased interferon production (Fig. 4D). The cMo subpopulation upregulated the highest number of pro-inflammatory cytokines that we measured, including IFN-a, IFN-g, IL-12p40, TNFa, and IL-6 (Fig. 4E). We evaluated cytokine co-expression in the cMo population (Fig. S12A) in order to determine whether these cytokine responses were arising from different cells or whether the same cells could be driving both interferon and pro-inflammatory responses. While single-positive IL-6 and IFN-a cMo were more highly abundant post-vaccination, we also detected significant increases in a relatively small population of cells co-expressing both cytokines (Fig. S12B). We also observed significant increases in circulating cMo cells in the blood at D1, a pattern which has previously been reported following Tdap-IPV booster vaccination^27^. Finally, the iMo subpopulation increased IFN-a and IL-12p40 production (Table S4). To determine whether changes in APC activation were associated with vaccine-induced antibody responses, we cross-correlated D1-D0 cell abundance or cytokine expression values with adjusted antibody LFCs. This analysis revealed that the strongest correlations were between changes in TNFa/IL-6 expression in classical/intermediate monocytes, and the 1-year Prn and FHA antibody responses (Fig. S12C). Overall, the early immune response to Tdap-IPV vaccination is characterized by an efflux of T-cells and DC2 cells. In parallel, an absolute increase in the number of circulating granulocytes and monocytes was observed shortly after vaccination, coupled with the induction of inflammatory cytokine and type I IFN production in monocytes and DCs, in particular IFN-a.

### Tdap-IPV induces antiviral gene expression in monocytes and dendritic cells

**Fig. 5:**
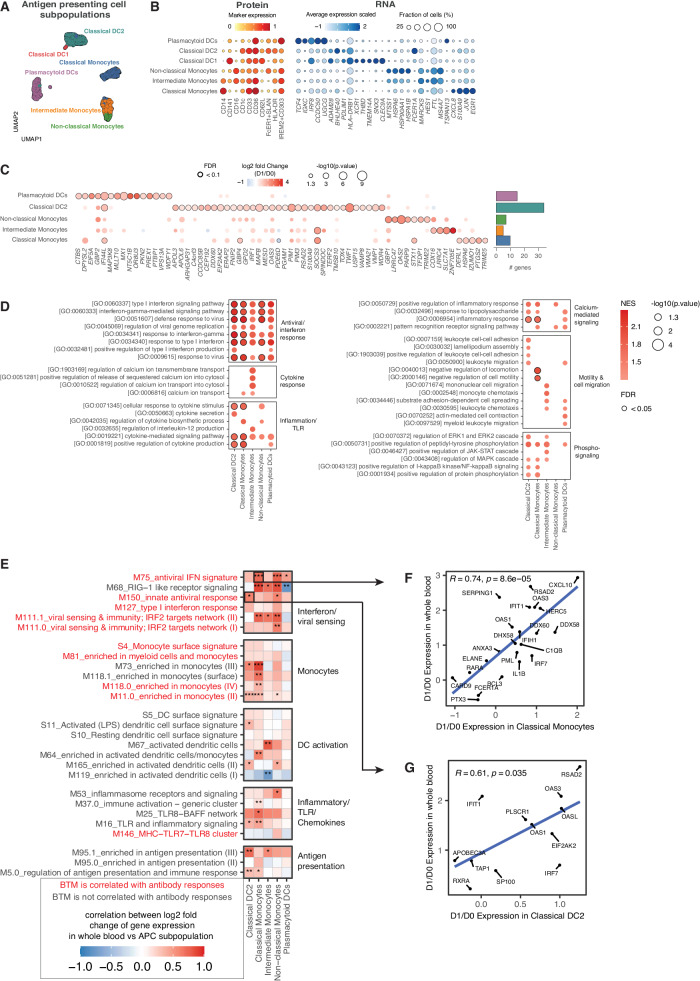
Single-cell RNA sequencing reveals type-I interferon and anti-viral gene expression in antigen presenting cells. **A** UMAP visualization of all single-cells (*N* = 6348 cell across 14 blood samples representing two timepoints (D1 and D0) from *N* = 7 participants of the Netherlands cohort) colored according to subpopulation with (**B**) dot plots of protein markers (left panel) and RNA expression profiles of top discriminating genes (right panel). **C** Upregulated differentially expressed genes (DEGs) 1 day after vaccination are marked with a black border. Expression of DEGs is also shown for other subpopulations if the nominal *p* < 0.05. Right margin: the number of upregulated DEGs for each subpopulation. Statistical significance was calculated with a negative binomial linear model and false-discovery rate (FDR) adjusted two-sided *p* values were calculated. Upregulated DEGs were defined as log2 fold change (D1/D0) > 0, FDR < 0.1. **D** Gene set enrichment analysis (GSEA) was used to calculate the normalized enrichment score (NES) of GO terms for each subpopulation using a list of all genes ranked by the log2-fold change over baseline (D1/D0). Statistical significance and *p*-values were calculated against an empirical null distribution and reflect two-sided tests. FDR adjusted *p* values were calculated. Selected GO terms are shown (nominal *p* < 0.05) and those with FDR < 0.05 are highlighted with a black border. **E** Correlations of whole blood BTM gene expression (y-axis) with gene expression of each APC subpopulation (x-axis). BTMs that are enriched at D1 in whole blood of the Netherlands cohort are shown on the y-axis (related to Fig. 2B) and are colored according to whether those BTMs are correlated with antibody responses (related to Fig. 3). Heatmap color indicates Pearson’s correlation coefficient. The degree of statistical significance (nominal two-sided *p* value) is also shown * *p* < 0.05; ** *p* < 0.01; *** *p* < 0.001. BTMs are grouped based on their biological function on the right margin. **F** Correlations between log2 fold change (D1/D0) of gene expression in whole blood vs classical monocytes for the M75_antiviral IFN signature BTM (*N* = 22 genes), and (**G**) correlations between gene expression in whole blood vs classical DC2 for the M150_antiviral IFN signature BTM (*N* = 12 genes). Genes in each module are labeled with Pearson’s correlation coefficient and nominal two-sided *p* value. Source data are provided in the Source Data file.

The observed induction of transcriptional responses in whole blood, specifically related to antiviral and interferon BTMs, may occur alongside changes in APC abundance or cytokine production. In order to determine changes in the transcription of individual APC subpopulations, we performed single-cell RNA sequencing. Within the NL cohort, circulating APCs were isolated by fluorescence-activated single-cell sorting (FACS) for single-cell RNA sequencing at D0 and D1. Our approach leverages the resolution of single-cell protein and RNA expression levels to define subpopulations, which were then used to generate “pseudobulk” samples for cell-type specific differential expression analysis of vaccination responses (Methods, Fig. S13). In total, we identified six subpopulations of cells that correspond to the APC populations identified in the mass cytometry analysis (Fig. 5A). Analysis of protein and RNA expression profiles revealed characteristic genes and surface markers for each subpopulation (Fig. 5B). To identify transcriptional changes that were associated with vaccination, we performed differential gene expression analysis comparing the D0 and D1 transcriptomes for each subpopulation and examined the most highly upregulated DEGs (Fig. 5C). DC2 cells displayed the largest changes per gene and the most differentially expressed genes, many of which also displayed increased expression in other subpopulations, though to a lesser degree. To detect significantly affected pathways per subpopulation we performed GSEA on the total gene lists ranked by the log2-fold change (D1/D0) in expression and examined enriched gene ontology (GO) terms^34^ for each subpopulation. Enrichment of diverse GO terms highlighted several biological processes (Fig. 5D). GO terms for antiviral and interferon responses were the most strongly enriched and were present in all five subpopulations of cells. GO terms relating to cytokine responses and TLR signaling were also highly enriched, primarily in cMo and DC2 cells. We also observed enrichment of the JAK-STAT, NFkB, MAPK, and ERK phosphorylation dependent signaling pathways in cMo, iMo, and DC2 cells.

The enrichment of GO terms indicated that APC subpopulations upregulate type-I interferon and antiviral response pathways, consistent with the changes we observed by bulk gene expression and mass cytometry analysis. To further substantiate that changes in whole blood BTMs were at least partly due to changes in expression per subpopulation, rather than changes in the frequency of subpopulations, for each BTM enriched in the NL cohort (Fig. 2B), we correlated the log2-fold change (D1/ D0) of genes with the expression of those of each APC subpopulation (Fig. 5E). In this context, gene expression of most BTMs was primarily correlated with cMo, ncMo, and DC2 gene expression. BTMs that were also correlated with antibody responses (Fig. 4), such as *M75 antiviral IFN signature* and *M150 innate antiviral response* showed a high degree of correlation with expression levels for cMo (Fig. 5F) and DC2 cells (Fig. 5G), respectively. Since equal cell numbers at D0 and D1 were used to derive changes in gene expression for APC subpopulations (Fig. S13E), high correlations of these genes with blood gene expression levels indicate that intracellular transcriptional changes in these subpopulations, potentially alongside changes in cell abundance in blood post-vaccination, could explain the enrichment of BTMs.

### Inactivated poliovirus stimulates p38-mitogen-activated protein kinase signaling in monocytes and dendritic cells

**Fig. 6:**
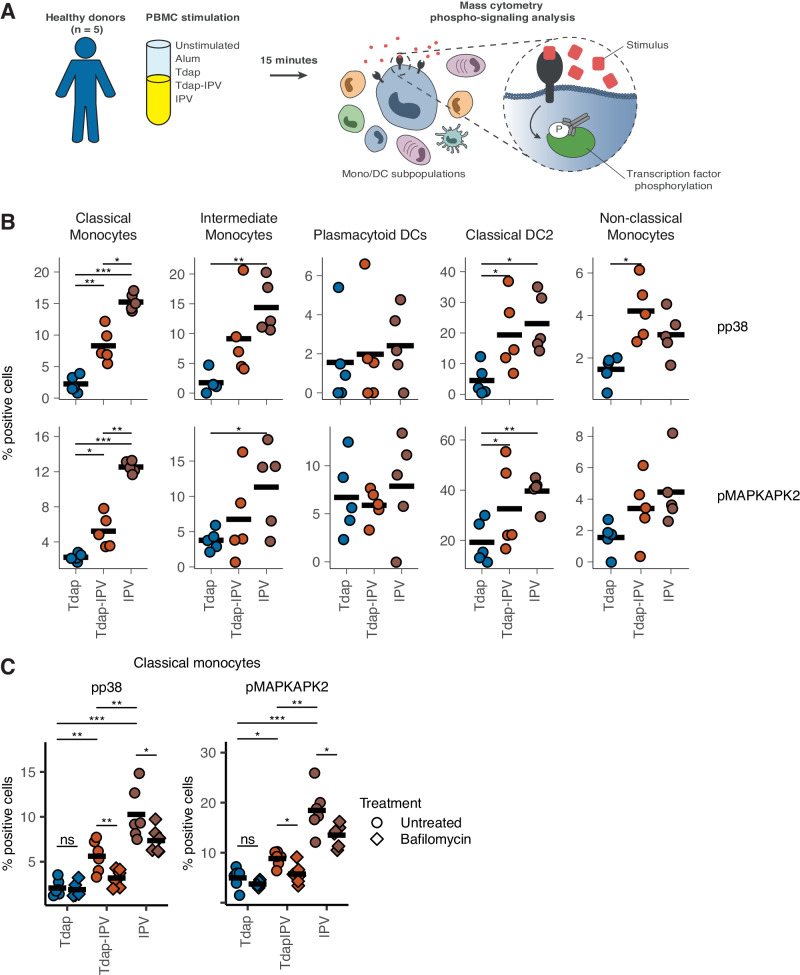
Inactivated poliovirus stimulates P38 and MAPK phospho-signaling responses in classical monocytes and dendritic cells. **A** Experimental plan for the analysis of phospho-signaling responses after stimulation with vaccines. PBMCs from healthy donors were left unstimulated or incubated for 15 min with one of the indicated stimuli. Mass cytometry was then used to identify innate immune cell populations and quantify phospho-signaling responses. **B** The proportion of cells expressing phosphorylated p38 (pp38) or MAPKAPK2 (pMAPKAPK2) in response to Tdap, Tdap-IPV, or IPV stimulation. Responses of five antigen presenting cell subpopulations are shown. Data are *N* = 5 healthy donors. Classical monocytes (pMAPKAPK2): TdapIPV vs Tdap *p* = 0.023; IPV vs Tdap *p* = 1.98 × 10^−5^; TdapIPV vs IPV *p* = 0.001; IPV vs TdapIPV *p* = 0.001. Classical monocytes (pp38): TdapIPV vs Tdap *p* = 0.009; IPV vs Tdap *p* = 2.41 × 10^−5^; IPV vs TdapIPV *p* = 0.016. Classical DCs (pMAPKAPK2): TdapIPV vs Tdap *p* = 0.039; IPV vs Tdap *p* = 0.004; Classical DCs (pp38): IPV vs Tdap *p* = 0.011; TdapIPV vs Tdap *p* = 0.045; Intermediate monocytes (pMAPKAPK2): IPV vs Tdap *p* = 0.03; Intermediate monocytes (pp38): IPV vs Tdap *p* = 0.002; Non-classical monocytes (pp38): TdapIPV vs Tdap *p* = 0.02. **C** In a second experiment, PBMCs were pre-incubated with bafilomycin or left untreated and were subsequently stimulated with the indicated vaccines. pp38 and pMAPKAPK2 expression is shown for classical monocytes. Data are of *N* = 6 healthy donors. Classical monocytes (pMAPKAPK2): TdapIPV.Bafilomycin vs TdapIPV.Untreated *p* = 0.012, IPV.Bafilomycin vs IPV.Untreated *p* = 0.036, Tdap.Bafilomycin vs Tdap.Untreated *p* = 0.15, TdapIPV.Untreated vs Tdap.Untreated *p* = 0.02, IPV.Untreated vs Tdap.Untreated p = 0.0002, IPV.Untreated vs TdapIPV.Untreated p = 0.0046; Classical monocytes (pp38): TdapIPV.Bafilomycin vs TdapIPV.Untreated *p* = 0.007, IPV.Bafilomycin vs IPV.Untreated *p* = 0.040, Tdap.Bafilomycin vs Tdap.Untreated *p* = 0.73; TdapIPV.Untreated vs Tdap.Untreated *p* = 0.004, IPV.Untreated vs Tdap.Untreated *p* = 0.0002, IPV.Untreated vs TdapIPV.Untreated *p* = 0.008. * *P* < 0.05, ** *P* < 0.01, *** *P* < 0.001, statistical significance (nominal *p* value) was determined using a two-sided paired *T* test. Horizontal black lines in each plot correspond to the sample mean. Source data are provided in the Source Data file.

Given the antiviral responses observed early after Tdap-IPV vaccination, we hypothesized that the presence of inactivated poliovirus (IPV) may play a role in activating monocytes and DCs. We examined differences in intracellular phospho-signaling induced by aP vaccines with and without IPV in vitro. PBMCs from healthy donors were stimulated for 15 min with Tdap, Tdap-IPV, IPV, alum, or phosphate-buffered saline (Fig. 6A), after which samples were analyzed by mass cytometry with a panel of phenotypic APC and phospho-protein markers (Table S5, Fig. S14). Whereas Tdap-IPV and IPV induced significant phosphorylation of p38 (pp38) and MAPKAPK2 (pMAPKAPK2) in cMo and DC2, Tdap in the absence of IPV did not (Fig. 6B). Of note, although the Tdap-IPV and IPV vaccines used here contain the same number of poliovirus particles, differences in the phosphorylation signal were observed between the two vaccines, such that IPV induced the highest phosphorylation of p38 and pMAPKAPK2 in cMo. Compared to unstimulated cells, both Tdap-IPV and IPV induced phosphorylation of p38 and MAPKAPK2 in cMo and DC2 cells (Fig. S15). To investigate whether activation of the p38-MAPK pathway was dependent on TLR signaling, we repeated the experiment in the presence of bafilomycin A1, which blocks signaling through endosomal TLRs by inhibiting endosomal acidification^35^. Bafilomycin A1 treatment significantly, but not completely blocked p38 and pMAPKAPK2 phospho-signaling in cMo cells following Tdap-IPV and IPV stimulation (Fig. 6C), while only a minimal effect was observed after Tdap stimulation. These findings suggest that intracellular sensing of IPV by endosomal TLRs expressed by classical monocytes and other APCs may be important for driving the downstream inflammatory response to Tdap-IPV vaccine components.

### Antiviral responses induced by Tdap-IPV vaccination are enhanced compared to Tdap

**Fig. 7:**
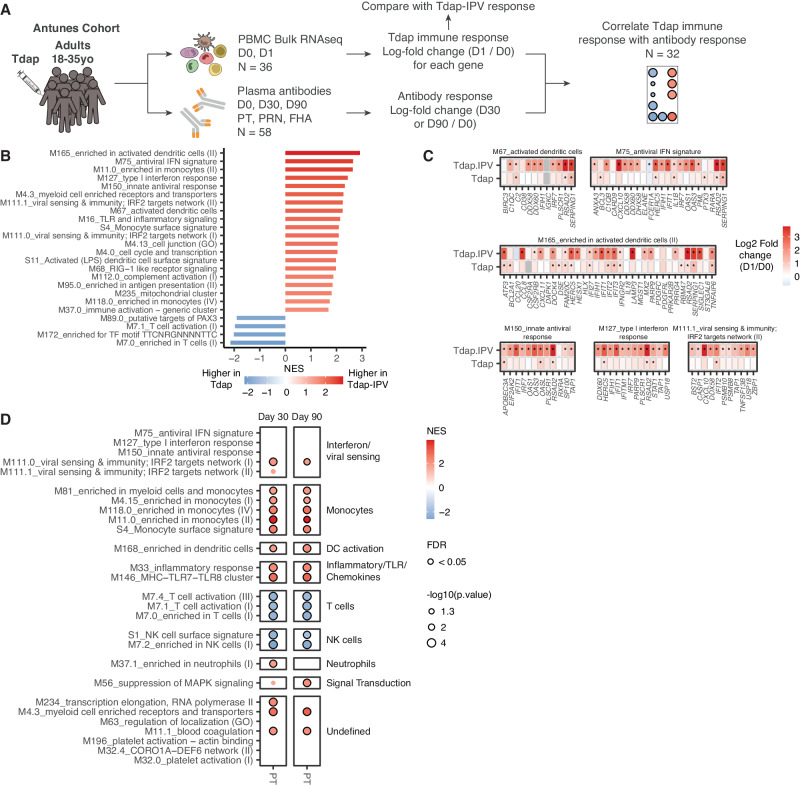
Tdap-IPV induces stronger anti-viral gene expression compared to Tdap. **A** Approach for comparing the effects of Tdap and Tdap-IPV vaccination. **B** Gene set enrichment analysis (GSEA) was performed on a list of genes ranked by the difference in log2-fold change (D1/D0) between Tdap-IPV (combined NL and UK cohorts) and Tdap vaccination (Antunes cohort). Significantly enriched (FDR < 0.05) blood transcription modules (BTMs) are shown. **C** Genes of selected BTMs from (**B**) are shown with the log2-fold expression (D1/D0) of Tdap-IPV (combined NL and UK cohorts) and Tdap vaccines. Differentially expressed genes (DEG, FDR < 0.05 and | Log2 fold change| > 0.5) are marked with an asterisk. Statistical significance was calculated with a negative binomial linear model and FDR adjusted two-sided *p* values were calculated. **D** Dot plot of selected BTMs (rows) whose activity 1 day post-vaccination (D1/D0) is associated with the adjusted log10 fold change of PT-specific antibody responses (columns) 30 days (Day 30/Day 0) or 90 days (D90/Day 0) post Tdap vaccination. Data are *N* = 32 participants in the Tdap cohort. GSEA was used to identify positive (red) or negative (blue) enrichment of BTMs within pre-ranked gene lists, where genes were ordered according to their correlation between gene expression and antibody response. Selected BTMs that are associated with antibody responses after Tdap-IPV vaccination (related to Fig. 3) are labeled on the y-axis, and significant correlations of those BTMs with the Tdap-induced PT antibody response are shown (nominal *p* < 0.05), enriched BTMs with FDR < 0.05 are highlighted with a black border. BTMs are annotated according to biological function on the right. Statistical significance and *p*-values of GSEA analyses (**B**, **D**) were calculated against an empirical null distribution and reflect two-sided tests. False discovery rate (FDR) adjusted *p* values were calculated. Abbreviations: FHA filamentous hemagglutinin, PRN pertactin, PT pertussis toxin, PBMC peripheral blood mononuclear cells. Source data are provided in the Source Data file.

An important question arising from our results is whether aP formulations that contain IPV induce different innate immune responses compared to those without IPV, and whether this might impact the adaptive immune response to aP antigens. In the absence of a randomized clinical trial to formally compare these vaccines, we explored this question by aligning the results of our study with an external dataset from a recently published Tdap booster vaccination study of aP and wP-primed adults^26^ (Fig. 7A). Since early immune response patterns following Tdap vaccination at 1 day post immunization were not different between aP and wP-primed individuals^26^, all participants across both priming backgrounds were pooled for downstream analysis to compare Tdap and Tdap-IPV immune responses. While Tdap generally induced a similar gene expression profile to Tdap-IPV 1 day post-vaccination, including up-regulation of monocyte, DC activation, antiviral inflammatory BTMs, T- and B-cell BTMs (Fig. S16), Tdap-IPV vaccination induced significantly higher antiviral, interferon, monocyte, and DC BTM responses 1 day post-immunization than the Tdap vaccine (Fig. 7B). Similarly, compared to Tdap vaccination, we observed increased differential expression of antiviral genes and ISGs after Tdap-IPV vaccination (Fig. 7C). Tdap vaccination significantly increased IgG against FHA and PT at 30 days and 90 days post-immunization (Fig. S17A). Because the antibody response to PT was the strongest (Fig. S17B), displayed a typical vaccine response, and was highly correlated with D0 antibody levels (Fig. S17C), we calculated the adjLFC for anti-PT IgG and determined correlations with transcriptional responses after 1 day. Like Tdap-IPV vaccination, PT antibody responses were highly correlated with BTMs for antigen presentation, DC activation, monocytes, and inflammation. However, in contrast to Tdap-IPV vaccination, minimal positive correlations were observed with BTMs for interferon and antiviral responses (Fig. 7D). Altogether, these results suggest that the presence of IPV in Tdap-IPV vaccines induces an antiviral response to vaccination, the strength of which is associated with the antibody response to co-administered pertussis antigens.

## Discussion

We applied a systems vaccinology approach to identify early correlates of the humoral response in two cohorts of adolescents and to characterize the innate response following Tdap-IPV vaccination. We found that Tdap-IPV vaccination induced a marked up-regulation of transcriptional responses relating to monocytes and DCs, as well as pro-inflammatory, antiviral, and interferon genes in both cohorts 1 day after vaccination that were correlated with the persistence of antibody responses 1-year after vaccination for multiple pertussis vaccine antigens. Variation in antibody responses were observed between the UK and NL cohorts. This might be explained by differences in pre-existing memory, since baseline pertussis-specific memory B cells were significantly higher in the UK cohort compared to the NL cohort (Fig. S3B) and is possibly related to increased exposure to *B. pertussis* in this population. We also observed that the baseline levels of Dt and TT IgG was significantly higher in participants of the NL than in the UK cohort. This is likely caused by differences in the timing of Td-IPV booster vaccination, which in the Netherlands is given at 9 years of age, but in the UK at 14 years of age^36^. Consequently, at study inclusion the Dutch but not the UK participants had already received a booster dose of Td-IPV. In line with this, we observed that IgG responses to Dt and TT antigens were overall higher in the UK cohort than in the NL cohort (Fig. S2).

Our result that Tdap-IPV stimulates antiviral responses that are correlated with the induction of antibodies is consistent with systems-level studies investigating virus-containing vaccines against yellow fever^15,17^, influenza^16,17,19,20^, and Ebola^21^, where antiviral and interferon responses 1 day post-vaccination were highly associated with adaptive immune responses, including antibody and T-cell responses. Notably, an antiviral interferon signature was not observed following vaccination with a pneumococcal vaccine that is composed of bacterial components^22^. These results indicate that the Tdap-IPV vaccine, which is a multi-component and multivalent vaccine, may be immunologically more similar to a viral vaccine rather than to a bacterial vaccine.

An important question arising from our results is whether the presence of IPV in aP vaccines stimulates augmented immune responses compared to aP formulations without IPV. Prior to our study, the potential role for IPV in modulating immune responses to pertussis in multivalent vaccines had not been described, and consequently, a principal limitation of our study is that it is not a randomized clinical trial. While our study design cannot directly verify whether IPV enhances pertussis vaccine immunogenicity, we attempted to circumvent this limitation by comparing the results of our study that used a Tdap-IPV vaccine to those of a recently published Tdap booster vaccination study^26^. Although the two studies vary with regards to e.g., age and pertussis vaccine formulation, a key difference was that Tdap-IPV vaccination induced significantly higher antiviral, interferon, monocyte, and DC gene expression responses than Tdap vaccination. This suggests that the presence of IPV in Tdap-IPV vaccines may potentiate innate immune responses (Fig. 7B). Our results also indicate that IPV may modulate the antibody response to co-administered pertussis antigens, as was previously suggested for *Haemophilus influenza* type b^37^. Positive correlations between innate immunity-related BTMs and aP vaccine antibody responses largely overlapped between Tdap and Tdap-IPV vaccination, apart from those BTMs relating to antiviral and interferon responses (Fig. 7F). Nevertheless, a randomized controlled trial is needed to address whether co-administration of IPV increases persistence of the pertussis antibody responses.

Within the NL study cohort, which was comprised of aP- and wP- primed adolescents, we could not detect differences between these groups in the early immune response to the Tdap-IPV vaccine or in the antibody response. This result is largely in agreement with the Da Silva Antunes et al. study, which reported similar responses at 1 day post-vaccination^26^. It should be noted that 30% of aP-primed participants in this cohort displayed a divergent inflammatory immune response pattern of 14 genes at 7 days post Tdap boosting, as well as 36 genes at 3 days post-vaccination, possibly reflecting qualitative differences in the adaptive response following priming with aP or wP vaccines. In addition, this subset of aP-primed study participants also displayed increased post-boost PT-specific IgE responses, as well as differences in the pre- and post-boost levels of IgG3 and IgG4 subclasses for certain antigens. However, we note that there were no differences reported in overall pertussis antigen IgG levels, which coincides with the results of our study. In our study cohorts, which were relatively small and examined transcriptomic responses after 1 day, we could not directly verify the results reported by da Silva Antunes et al. Further studies are needed to explore the specific effects and heterogeneity resulting from aP priming in infancy. Although small gender-specific effects on gene expression were observed in the UK cohort, the limited sample size of our cohorts precluded a thorough investigation of the effect of gender on pertussis vaccine responses.

Using complementary single-cell technologies, we identified cellular components that drive the innate immune response to Tdap-IPV vaccination. At the level of individual APC subpopulations, we observed increased antiviral, interferon, and inflammatory cytokine responses in classical monocytes, intermediate monocytes, CD1c+ classical DC2, and plasmacytoid DCs. This result is concordant with our transcriptome analysis of these APC subpopulations that identified differential expression of ISGs, as well as enhanced pathway-level antiviral and interferon responses. While changes in the abundance of APC subpopulations might partially explain the enrichment of antiviral and interferon BTMs in blood, including those that were correlated with antibody responses, our results show that gene expression of these BTMs was highly correlated with intracellular gene expression changes in monocytes and DC2 cells, but not pDCs (Fig. 5E). A potential explanation may be that myeloid APCs are responsible for simultaneously sensing aP vaccine antigens and responding to IPV, a process which ultimately shapes the magnitude of the antibody response. In line with this, there is evidence that type-I interferon signaling in myeloid APC subpopulations drives maturation and tunes APC co-stimulatory profiles, which may impact T-cell responses^38^. Mechanistically, the high correlations we observed of monocyte, dendritic cell, antiviral, and interferon BTMs with pertussis antibody responses 1 year after vaccination point to the activation of long-lived plasma cells (LLPCs) that reside in the bone marrow^39^. Type-I interferon responses have been reported to promote B-cell activation and antibody responses to soluble protein antigens^40,41^. Furthermore, type-I interferons also enhance T-cell responses, which in turn promote B-cell development^42^. A limitation of our study is that we did not measure pertussis-specific T-cell immunity, as Th17 and Th1 immune responses are considered important for protection against pertussis^43^. In contrast to many systems vaccinology studies that analyze immune responses in adults, our study cohort consisted of adolescents, a population with a high pertussis disease burden and transmission^23–25^. One consequence is that serial blood draws in this population are restricted and thus limited our ability to examine responses at other timepoints, including plasmablast responses and T-cell responses that peak within one to two weeks post aP vaccination^5,44^. However, we did observe an enrichment of T-cell transcriptional modules that were positively associated with the D28 antibody response induced by Tdap-IPV (Fig. 3).

We speculate that IPV influences the induction of antibodies against co-administered vaccine antigens, effectively acting as a vaccine adjuvant. In our study, in vitro stimulation with Tdap-IPV or IPV induced significantly higher p38 and MAPKAPK2 phosphorylation in monocytes and DC2 cells than Tdap (Fig. 6). These phosphoproteins are part of the MAP-K signaling pathway that is downstream of Toll-like receptor (TLR) sensing and induces the expression of genes that regulate the inflammatory response. This suggests that IPV contains pathogen-associated molecular patterns (PAMPs) that specifically activate myeloid APCs. One mechanism through which IPV may stimulate innate immune responses is through viral RNA, which is present in commercial formulations of IPV vaccines^45^. Viral single-stranded RNA that is contained within inactivated poliovirus particles is detected in humans by the endosomal receptors TLR8 and TLR7. While TLR8 and TLR7 are structurally similar and both recognize ssRNA, they preferentially bind AU- or GU-rich sequences respectively^46^. Of note, TLR8 and TLR7 exhibit different cellular expression patterns in humans, with TLR7 being mainly expressed by B cells and pDCs and TLR8 primarily expressed in monocytes, myeloid DCs and neutrophils^47^. In light of the 54% AU-content of the poliovirus genome^48^ and the in vitro stimulation data, our results point to a role for TLR8 in the recognition of IPV rather than TLR7. Of note, preclinical studies that examined immunity to pertussis, including recent baboon studies, rarely, if ever, use licensed formulations of aP vaccines that contain IPV^18,49,50^. Given that mouse TLR8 does not recognize ssRNA ligands or RNA viruses due to the deletion of five amino acids that are necessary for RNA binding by human TLR8^51^, it is uncertain whether the antiviral response to IPV in mice is similar to that in humans. Interestingly, animal studies have demonstrated that inclusion of ligands that target endosomal TLR7, which in contrast to TLR8 is fully functional in mice, enhances immune responses to pertussis and protection^52^, demonstrating that engagement of TLRs that recognize ssRNA can modulate immunity to pertussis. Similarly, mice that received a vaccine containing nanoparticles with TLR7 ligands as adjuvants with soluble protein antigens displayed durable antibody responses and persistent LLPCs that required the activation of multiple DC subsets^53^. Vaccination studies in non-human primates that use TLR7/8 agonists as adjuvants have also highlighted their potential to induce persistent antibody responses and LLPCs^54^. Possibly, species-specific innate immune responses to IPV may contribute to the observed discrepancy in skewing of pertussis-specific memory T-helper cells between mice and humans following aP vaccination^55^.

Our results suggest that there may be a previously unappreciated role for IPV in protecting against pertussis, warranting epidemiological studies that compare pertussis vaccine formulations to provide formal evidence of the effect of IPV co-administration. Currently, the role of IPV in enhancing immunity against other diseases has not been investigated in epidemiological studies, although previous studies have suggested that co-administration of IPV enhances IgG and Th1 T-cell responses to vaccination against other co-administered antigens such as TT and the *Haemophilus influenzae* type B capsular PRP antigen^37,56^. We speculate that the current level of immunity against pertussis in the population is co-dependent on the presence of IPV in aP formulations. While the first generation of pediatric DTaP-combination vaccine formulations were developed and licensed without IPV, subsequent non-inferior immunogenicity trials supported the inclusion of IPV in aP-based pediatric vaccination programs^4,57–59^. Whereas early reports on rapid waning of protection against pertussis following aP vaccination were based on vaccines that did not yet include IPV^11,60^, most pentavalent and hexavalent aP-containing vaccines that are used in pediatric vaccination programs today include IPV^61,62^. This may partially explain the apparent discrepancy between those early epidemiological studies and more recent mathematical modeling of pertussis re-emergence that indicates that immunity conferred by aP vaccines wanes more slowly than widely believed^25,63^. Importantly, IPV is not universally included in aP booster vaccines, which are available with or without IPV^64^. Future changes in the use of IPV may be expected, given the planned withdrawal of oral poliovirus vaccines as part of the Polio Eradication and Endgame Strategic Plan of the Global Polio Eradication Initiative^65^. Furthermore, there is increasing discussion about reducing the number of IPV doses in booster vaccination programs in geographic regions that have eliminated poliovirus infections^66^. Our study suggests that one solution in preparation for the anticipated withdrawal of IPV from national immunization programs may be to supplement aP-based vaccine formulations with adjuvants other than aluminum salts. Future studies that directly compare multivalent vaccines with or without IPV are needed in order to determine its potential off-target effects on immunity in different populations.

## Methods

### Study design

This human clinical study was designed and conducted in accordance with the provisions of the Declaration of Helsinki (1996) and the International Conference on Harmonisation Guidelines for Good Clinical Practice. The trial is registered at the EU Clinical Trial database (EudraCT number 2016-003678-42) and was approved by the Medical Research Ethics Committee United (MEC-U, NL60807.100.17-R17.039) in the Netherlands and the South Central - Hampshire B Research Ethics Committee (REC, 19/SC/0368) in the UK. Written informed consent was provided by participants and/or legal guardians at the start of the study, after the nature and possible consequences of the studies were explained. Clinical data of participants was recorded using OpenClinica, electronic case record form software that enables compliance with regulatory guidelines such as 21 CFR Part 11. The same online database system was used across the Dutch and UK sites. In the Netherlands, participants/parents/legal guardians were asked to keep their vaccination booklets at hand for the first visit. In case participants/parents/legal guardians did not have a vaccination booklet anymore, permission was asked to contact DVP (Vaccine Supply and Prevention Programs Service) to check their vaccination status according to NIP. In the UK, vaccination history was obtained either from the child’s “Red Book”, or alternatively by means of the participant seeking confirmation from the GP recorded on a standard document.

The aim of this study was to identify early molecular and cellular correlates in peripheral blood that are associated with the humoral response that is mounted in response to Tdap-IPV vaccination in healthy adolescents. This study was conducted as part of a larger multi-center phase IV clinical study that evaluated immune responses to Tdap-IPV vaccination in participants from the United Kingdom, Finland, and the Netherlands^7^. Participants in the Dutch arm of the study were enrolled between October 2017 and March 2018. Participants were recruited by mail-outs in the Hoofddorp region, facilitated by the Municipal Administration, and the study was conducted by the Spaarne Academy (Spaarne Hospital, Hoofddorp, the Netherlands). Participants in the UK cohort were enrolled between April 2018 and January 2020 via mail out to eligible participants within postal areas. Participants in the NL or UK cohorts were included if they were in good health and had received all regular vaccines for their age group according to the Dutch or UK national immunization program (NIP), including an aP booster vaccination in preschool. Sex was not explicitly considered in the study design and all children between ages 11 and 15 years old were eligible for inclusion in the study. Male and female adolescents with either aP or wP vaccination priming backgrounds were included in the present study (NL cohort: *N* = 14, 8 males and 6 females; UK cohort: *N* = 12, 6 males and 6 females), Table S1. Assignment of aP or wP background in this study was based on the participant date of birth and the available pertussis vaccine in the Netherlands at that time^7^. The number of participants included in the present study was informed on the basis of feasibility for carrying out a study for systems vaccinology. All participants received a dose of Tdap-IPV vaccine (Boostrix^TM^-IPV, GlaxoSmithKline (GSK), Wavre, Belgium) administered by intramuscular injection in the deltoid muscle of the upper arm at baseline (D0). The primary outcome was pertussis toxin-specific IgG antibody concentration at D28 post-vaccination. Secondary outcomes include but are not limited to IgG responses against the other Tdap vaccine antigens TT, Dt, Prn, FHA at D0, D28, and Y1, and PT at D0 and Y1. Exploratory outcomes relevant for this manuscript were measured at D0 and D1 post-vaccination, whole blood transcriptomic analysis, measurements of immune cell abundance and cytokine production by mass cytometry, and single-cell gene expression profiling of innate immune cells.

### Participant samples

Whole blood was collected in tubes containing sodium heparin (Greiner vacuette in 4 ml, 6 ml, and 9 ml volumes, catalog numbers 454030, 456028, 455051) to prevent coagulation. Complete blood counts were obtained using a Sysmex XN-450 hematology analyzer and sera were stored at −20 ^o^C until analysis. Whole blood was processed for single-cell RNA sequencing or mass cytometry analysis (detailed below). Whole blood was also collected directly in PAXgene Blood RNA Kit tubes (PreAnalytix) for transcriptome analysis and frozen at −80 ^o^C until processing.

### Serological Analysis

Sera were analyzed for PT-, FHA-, Prn-, TT-, and Dt-specific IgG antibody concentrations using a fluorescent-bead-based multiplex immunoassay^29^. Antigens were covalently coupled to distinct color-coded activated carboxylated MicroPlex Microspheres (beads) (Luminex, Austin, Texas, USA) according to the manufacturer’s instructions. The following antigens were used for coupling: PT (GSK, Belgium), FHA (Sanofi, France), Prn (GSK, Belgium), diphtheria toxoid (Netherlands Vaccine Institute) and TT (T3194, Sigma-Aldrich, Saint Louis, Missouri, USA). After a wash step in PBS, 12.5×10^6^ carboxylated beads/mL were activated in PBS containing 2.5 mg of 1-ethyl-3-(-3-dimethylaminopropyl)-carbodiimide hydrochloride (Thermo Fisher Scientific, Waltham, Massachusetts, USA) and 2.5 mg of N-hydroxy-sulfosuccinimide (Thermo Fisher Scientific, Waltham, Massachusetts, USA). The antigens for coupling were diluted in PBS to a concentration of 10ug of PT, FHA or Prn, 100ug of Dt or 25ug of Tt per 12.5 × 10^6^ activated beads and incubated for 2 h at room temperature in the dark under constant rotation. After 3 wash steps the antigen-coupled beads were stored in the dark in PBS containing 0.03% (wt/vol) sodium azide and 1% (wt/vol) bovine serum albumin at 4 °C until use.

Sera diluted 1/200 and 1/4000 in PBS containing 0.1% (vol/vol) Tween 20 and 3% (wt/vol) BSA were incubated with antigen-coupled beads in a 96-well filter plate for 45 min at room temperature at 750 rpm in the dark. Reference sera in a dilution series, quality control sera and blanks were included on each plate. The in-house reference standard for pertussis was calibrated against WHO 1st IS Pert 06/140 and serially diluted 4-fold over 6 wells (1/200 to 1/204800). The in-house reference standard for Dt and Tt was calibrated against WHO NIBSC DI-3 and TE-3 and serially diluted 4-fold over 8 wells (1/50 to 1/819200). Following incubation, wells were washed 3 times with PBS, incubated with R-phycoerythrin-labeled goat anti-human IgG antibody (Jackson Immunoresearch Laboratories, West-Grove, PA, USA, catalog number, 109-115-098, 1:200 dilution) for 30 min and washed. Beads were included in PBS and median fluorescence intensity (MFI) was acquired on a Bio-Plex LX200. MFI was converted to IU/mL by interpolation from a 5-parameter logistic standard curve using Bioplex Manager 6.2 software (Bio-Rad Laboratories, Hercules, California, USA) and exported to Microsoft Excel.

Sera in the NL cohort were also analyzed for Polio.I, Polio.II, and Polio.III-specific IgG antibody concentrations using a fluorescent-bead-based multiplex immunoassay^28^. Type-specific capture monoclonal antibodies (MAb), respectively antipoliovirus type 1 clone 9B4 (HYB 295-17-02 ThermoFischer scientific, Waltham, MA USA), type 2 clone 24E2 (HYB 294-06-02 ThermoFischer scientific) and type 3 clone 4D5 (HYB 300-06-02 ThermoFischer scientific) were conjugated to three distinct activated carboxylated microspheres. Briefly, 800ul of carboxylated microspheres (12.5 × 10^6^ beads/ml; Bio-Rad Laboratories, Hercules, CA USA) were activated by adding 100 μl of 50 mg/ml N-hydroxy-sulfosuccinimide (sulfo-NHS, ThermoFischer scientific) and 100 μl 50 mg/ml 1-ethyl-3-(-3-dimethylaminopropyl)-carbodiimide hydrochloride (EDC, ThermoFischer scientific) in PBS. The microspheres were activated at room temperature for 20 min in the dark under constant rotation, washed once with 1 ml PBS and resuspended in 1 ml PBS containing 50 μg/ml of antipoliovirus monoclonal antibody. The beads were incubated for 2 h at RT in the dark under constant rotation. Subsequently, the beads were washed three times with PBS and stored in the dark in PBS containing 0.05% (wt/vol) sodium azide and 1% (wt/vol) bovine serum albumin at 4 °C until use.

An in-house reference standard serum (RIVM MIA reference standard serum, Bilthoven, the Netherlands) was calibrated against the 3rd International Standard antiPoliovirus serum Types 1, 2 and 3 (NIBSC code: 82/585 assigned potency 11/32/3 IU/ml) and used as the reference standard in the polio MIA assay. After calibration, the international standard was used as a control serum.

For quantification of Polio.I, Polio.II, and Polio.III antibodies in sample sera, reference standard serum, and control serum, sera were diluted and pre-incubated with monovalent IPV type 1, 2, 3 (National Vaccine Institute NVI/Bilthoven Biologicals, the Netherlands^28^) The reference standard (RIVM MIA reference standard serum) was diluted in 10 steps of 1.5-fold dilutions (1/18–1/692). Sample sera were diluted 1/2 and 1/25 and the control serum diluted 1/20 in dilution buffer (PBS containing 0.25% (v/v) Tween-20, 1% (w/v) BSA and 0.5 M NaCl). Subsequently all dilutions were mixed 1:1 (v/v) with IPV in dilution buffer containing IPV type 1:56 DU/ml, IPV type 2:16 DU/ml and IPV type 3:80 DU/ml, ergo resulting in a final reference serum dilution of 1/36–1/1384, in serum dilutions of 1/4 and 1/50, in a control serum dilution of 1/40 and a IPV concentration of 0.7/0.2/1.0 DU/well (25ul) for IPV type 1, 2 and 3. After 2 h incubation on a shaking platform (1000 RPM) at RT, 25ul of the samples, reference standard and controls dilutions were transferred to a pre-wetted 96-well Multiscreen HTS filter plate (Millipore Corporation, Billerica, MA) containing a 25ul per well mix of antipoliovirus MAb type 1, 2 and 3 conjugated microspheres (4000 beads/region/well) in dilution buffer. The plates were incubated for 1 h at room temperature in the dark on a plate shaker at 600 rpm. The beads were collected by filtration using a vacuum manifold and washed three times with 100ul PBS. A recombinant Human CD155/FC chimera (rhCD155/Fc −100 μgr, Sino Biological Inc. Beijing, China) was labeled with R-phycoerythrin (RPE) using the SiteClick RPE antibody labeling kit (ThermoFischer/life technologies) following manufacturer’s instructions and used for detection of IPV type 1, 2 and 3. To each well 50ul of a 1/2000 dilution of RPE conjugated CD155-Fc (1 mg/ml) in PBS was added and the plate was incubated for 30 min with continued shaking (600 rpm). Plates were washed and beads were included in PBS and median fluorescence intensity (MFI) was acquired on a Bio-Plex LX200. MFI was converted to IU/mL by interpolation from a 5-parameter logistic standard curve using Bioplex Manager 6.2 software (Bio-Rad Laboratories, Hercules, California, USA) and exported to Microsoft Excel.

### RNA-sequencing from whole blood

RNA extraction from whole blood, library preparation, sequencing, data processing, and differential gene expression analysis is described in the Supplementary Information.

### Whole blood differential gene expression analysis

Differential gene expression analysis of pre- (D0) and post-vaccination (D1) blood samples was performed with DESeq2 (v1.38.3), which takes a raw read count x sample matrix as input and includes library size normalization, estimation of dispersion, model fitting and gene filtering steps. We analyzed the effect of priming background (aP or wP vaccine) and sex on the vaccine response (D1 vs D0) with a model that included the background or sex variable, as well as its interaction with vaccination day and subject ID. A gene was considered differentially expressed if the FDR adjusted p-value was <0.05 and |log2 fold change| > 0.5. As such, we analyzed gene expression with a final model that specifies vaccination day as the covariate of interest and controls for subject-level variations. The output from the DESeq2 pipeline includes a gene matrix with log2 fold change (D1/D0) and FDR-corrected p-values, which was used for downstream analysis.

### Mass cytometry from whole blood

Processing of whole blood samples for mass cytometry is described in the Supplementary Information.

### Derivation of whole blood mass cytometry cell type, cell abundance, and intracellular cytokine responses features

FCS files were imported into Cytobank data analysis software (v6.1.2) and the arcsinh transform of marker expression values was calculated for downstream analysis. Major immune cell lineages were manually gated (Fig. S10A). For subpopulation analysis of APCs, manually gated monocytes, pDCs, and mDCs were exported per participant per timepoint, for a total of 24 FCS files (one pre- and one post-vaccination sample per participant for 12 participants). These files were imported into R (v3.6.1) for unsupervised clustering and subpopulation analysis according to a predefined workflow^67^. In total 285,780 APCs were identified across all 24 samples and were pooled for clustering, which was performed using the following markers: HLA-DR, CD69, CD31, CD86, CD16, CD123, CD33, CD14, CD1c, CD11c, CD62L, CD38, CD141, CD11b. Cluster markers were selected on the basis of their importance for describing heterogeneity and activation states among monocytes and dendritic cells. In total, 14 clusters were detected and manually annotated based on their phenotypic marker expression (Fig. S11A). Cluster abundance was similar across all samples (Fig. S11B). We derived a high-level stratification of cells for downstream analysis by manually merging clusters of APCs based on their similarity, which yielded eight subpopulations (phenotypic marker expression and the mapping of clusters to subpopulations is provided in Fig. S11C). The frequency of each cell type and APC subpopulation in our mass cytometry data was calculated as a fraction of CD45+ cells for each sample. These frequency values were multiplied by the total leukocyte counts (Fig. S10D) per ml of blood per sample to derive cell type (Fig. S10E) and APC subpopulation (Fig. 4C–E) abundance values. Expression values for analysis of intracellular cytokine responses were calculated as the mean signal intensity of each subpopulation for each sample. In order to identify co-expression of IL-6 and IFN-a in classical monocytes (Fig. S12), per donor, classical monocytes from D0 and D1 samples were pooled and the 95% quantile was calculated for each cytokine. Cells that were above the 95% quantile were classified as positive, and cells below this threshold were classified as negative. Thereafter, the frequency of single-positive or double-positive cells was calculated among all cells within a D1 or D0 sample.

### Single-cell RNA sequencing from whole blood

Single-cell index sorting of immune cells, RNA extraction, library preparation, pre-processing of raw RNA sequencing data, filtering of low-quality cells, as well as pre-processing of single-cell FACS data is provided in the Supplementary Information.

### Multi-modal integration of single-cell RNA sequencing and FACS data

We defined subpopulations of APCs by integrating FACS marker and single-cell gene expression to define populations using multi-omic factor analysis (MOFA^68^), which enables variance decomposition of the datasets and factors using the coefficient of determination (*R*^2^). The MOFA model was constructed using the single-cell FACS marker data and the top 30 principal components of the single-cell RNA sequencing data. We followed the developer’s directions for model selection and downstream analysis (https://biofam.github.io/MOFA2/tutorials.html). Both FACS and single-cell gene expression contributed substantially to the variation in the final model. Furthermore, several factors were identified with shared contributions from each dataset, thereby highlighting the utility of a joint analysis (Fig. S13D). To define subpopulations, we used the single-cell x MOFA factor graph for unsupervised clustering. Similar to the mass cytometry analysis, single-cell data was clustered using a two-step approach. The clusters that were identified are presented in Fig. 6A, and their identity was verified by examining both single-cell surface marker and gene expression profiles. In total, we identified classical monocytes (cMo), intermediate monocytes (iMo), non-classical monocytes (ncMo), classical DC1 and DC2, as well as pDCs, which correspond to the same subpopulations previously defined in blood using integrated single-cell FACS and gene expression^69^.

### Pseudobulk differential expression analysis

We created ‘pseudobulk’ RNA libraries by computationally pooling the gene expression of cells in a given subpopulation for a given pre- or post-vaccination sample. Cells from different single-cell RNA sequencing batches were also pooled. The total number of cells in each pseudobulk sample is shown in Fig. S6E, where each colored segment for each subpopulation represents the number of cells in a pseudobulk sample. The classical DC1 subpopulation was removed from downstream analysis since no sample had >10 cells. Next, raw gene counts of cells within a pseudobulk sample were summed together. We used the DESeq2^70^ bulk RNAseq pipeline to perform differential gene expression analysis based on the developer’s directions (http://bioconductor.org/packages/devel/bioc/vignettes/DESeq2/inst/doc/DESeq2.html). We analyzed gene expression of each subpopulation separately with a model that specifies the number of days after vaccination as the covariate of interest and controls for subject-level variations. The output from the DESeq2 pipeline includes a gene matrix for each subpopulation with log2 fold change (D1/D0) and FDR-corrected p-values. These gene matrices were used for downstream analysis of differentially expressed genes (Fig. 5C), GSEA (Fig. 5D), and correlations with whole blood gene expression (Fig. 5E).

### Bioinformatic analyses

GSEA of whole blood and single-cell RNA sequencing data, as well as the comparison with external Tdap vaccination data from in ref. ^26^. is provided in the Supplementary Information.

### Phospho-signaling analysis of innate immune cells

Details of the stimulation of peripheral blood mononuclear cells (PBMCs) from healthy donors with vaccines and mass cytometry analysis are provided in the Supplementary Information.

### Statistical analyses

Antibody concentration values were log10-transformed to account for their skewed distribution. Antibody responses were calculated as log10-fold change (LFC) over baseline (D28 or Y1/D0). In order to account for the cross-correlation of LFC antibody responses with D0 antibody levels (Fig. S6), we calculated the adjusted LFC response as described previously in refs. ^32,33^. Briefly, for each antibody response, at each timepoint, and within each cohort, we fit a linear model with the LFC as the response and the log10 D0 antibody concentration as the predictor variable. Residuals from this model were extracted and summed with the intercept to obtain the adjLFC for each sample of every antibody response. Statistical comparisons of antibody concentrations, antibody responses, and phospho-signaling responses were calculated using a paired Wilcoxon test. Statistical parameters are reported directly in the figures and figure legends. Based on the similarity of antibody responses and transcriptional responses, participants were not separated based on sex or priming background in subsequent analyses (Mass cytometry and single-cell RNA sequencing analysis). Examination of the effects of Tdap-IPV vaccination with mass cytometry consisted of two parts: (i) differential expression (DE) of intracellular cytokines, and (ii) differential abundance (DA) analysis for each subpopulation of cells (Table S4). DE analysis was calculated using the mean signal intensity values for the indicated cytokines, for each subpopulation in each sample. DA analysis was performed on cell counts per unit of whole blood. For both DE and DA analyses, the effects of Tdap-IPV vaccination were determined using a linear mixed-effects model, fit with the lme4 (v1.1–33) R package^71^, with cell counts or mean signal intensity as the response, and vaccination day as the fixed effect. *P* values were generated using the lmerTest (v3.1–3) package^72^. We accounted for sample pairs (before and after vaccination) by introducing the subject ID as a random effect. P-values were extracted and corrected for multiple testing using Benjamini-Hochberg method^73^. Scatterplots displaying the correlation of LFC values with D0 antibody levels (Fig. S6, Fig. S17C) present Spearman correlation coefficient and associated p.value with linear regression trendline. Other statistical analyses (differential gene expression, GSEA) are reported in their respective section of the methods.

### Reporting summary

Further information on research design is available in the Nature Portfolio Reporting Summary linked to this article.

### Supplementary information

Supplementary Information

Reporting Summary

### Source data

Source Data

## Data Availability

Whole blood and single-cell RNA sequencing data of this study has been deposited to the GEO database (https://www.ncbi.nlm.nih.gov/geo/query/acc.cgi?acc=GSE195627). The processed data generated in this study are provided in the Source Data file. The raw data are available from the corresponding author upon reasonable request. The raw data are not publicly available due to data privacy laws. External whole blood RNA sequencing data analyzed in this study is also available on GEO. External antibody data analyzed in this study are available in the original report of the corresponding study ^26^. Source data are provided with this paper.

## Code availability

R code to replicate the findings of this study is available on the corresponding Github repository^74^. Any additional information required to reanalyze the data reported in this manuscript is available from the lead contact upon request.
