# Supplementary material for: Antiviral responses induced by Tdap-IPV vaccination are associated with persistent humoral immunity to Bordetella pertussis
Source: Nat Commun. 2024 Mar 8;15:2133. doi: 10.1038/s41467-024-46560-w (PMC10923912; doi:10.1038/s41467-024-46560-w)
Supplement: Supplementary file 1 — Supplementary Information [file 41467_2024_46560_MOESM1_ESM.pdf]

Supplementary Information for

Antiviral responses induced by Tdap-IPV vaccination are associated with persistent humoral immunity to pertussis

Joshua Gillard, Madeleine Suffiotti, Peter Brazda, Balaji Venkatasubramanian, Pauline Versteegen, Marien I. de Jonge, Dominic Kelly, Sagida Bibi, Marta Valente Pinto, Elles Simonetti, Mihaela Babiceanu, Andrew Kettring, Cristina Teodosio, Ronald de Groot, Guy Berbers, Hendrik G. Stunnenberg, Brian Schanen, Craig Fenwick, Martijn A. Huynen, Dimitri A. Diavatopoulos*

*Corresponding author. E-mail: dimitri.diavatopoulos@radboudumc.nl

This document includes:

Supplemental Methods
References (1-12)
Supplemental Figures:

Figure S1. Serum antibody concentrations induced by Tdap-IPV vaccination and comparison of antibody responses.
Figure S2. Post-vaccination Tdap-IPV antibody responses.
Figure S3. Baseline characteristics of the Netherlands and United Kingdom cohorts.
Figure S4. Differential gene expression of blood transcriptional responses one day post Tdap-IPV vaccination.
Figure S5. Molecular signatures induced by Tdap-IPV vaccination (continued from Figure 2B).
Figure S6. Post-vaccination antibody responses are negatively correlated with baseline antibody levels.
Figure S7. Molecular signatures associated with the adjusted antibody response induced by Tdap-IPV per cohort.
Figure S8. Molecular signatures associated with the adjusted inactivated poliovirus-specific antibody responses induced by Tdap-IPV.
Figure S9. Top BTMs and genes correlating with adjusted one-year antibody responses.
Figure S10. Mass cytometry analysis of circulating immune cells.
Figure S11. Subpopulation analysis of circulating antigen presenting cells.
Figure S12. Enhanced interferon-alpha and IL-6 cytokine co-expression in classical monocytes and correlations with adjusted LFC antibody responses.
Figure S13. Multi-modal single-cell RNA sequencing analysis of antigen presenting cells.
Figure S14. Gating strategy of immune cell populations for analysis of phospho-signaling responses.
Figure S15. Phospho-signaling responses of innate immune cells in response to Tdap, Tdap-IPV, and IPV stimulation compared to unstimulated cells.
Figure S16. Molecular signatures induced by Tdap vaccination.
Figure S17. Pre-vaccination and post-vaccination Tdap antibody responses.
Table S1. Participant characteristics and samples used for the various assays
Table S2. Antibody Log-fold change summary statistics and Anova
Table S3. Mass cytometry panel for analysis of circulating immune cells
Table S4. Mass cytometry differential cytokine and abundance analysis results
Table S5. Mass cytometry panel for analysis of phosphosignaling responses

Methods

RNA-sequencing from whole blood

Whole blood RNA extraction and library preparation

RNA was isolated from blood samples stored in Paxgene tubes according to the manufacturers' instructions (Qiagen). The extracted RNA samples were quantified using the Nanodrop 8000 UV spectrometer (Fisher Scientific). To assess RNA integrity, the samples were analyzed using a Tapestation 2200 (Agilent Technologies) using the R6K ScreenTape Reagents (Agilent) and concentration via the RNA HS assay on the Qubit.

Whole blood RNA sequencing

Globin transcripts in blood RNA were blocked with the Ribo-Zero Globin (Illumina) before library preparation. Libraries were prepared using the TruSeq Stranded Total RNA library preparation kit (Illumina). Libraries were validated, pooled at equimolar concentrations, and sequenced on an Illumina Next Seq 550. Files were converted from bcl to fastq using Illumina software (Illumina, Version 1.8.4), constructed as an integrated environment for preprocessing and analyzing NGS data.

Whole blood RNA seq data pre-processing

Raw FASTQ files were mapped to the human transcriptome (HG38) using the STAR aligner (*1*) and summation of gene counts per sample was performed with featureCounts (*2*). Genes with a biotype annotated as "protein coding" were used for downstream analysis. Library preparation failed for one baseline sample (~96% of genes with 0 or 1 count), this sample and its paired post-vaccination sample were excluded from analysis.

Mass cytometry from whole blood

Mass cytometry staining and data acquisition

Fresh blood aliquots (200 µl per participant, per timepoint) from 12 participant were incubated with 0.2 µl GolgiPlug (BD Biosciences) for 3 hours at 37˚C + 5% CO_2. Aliquots were then stained with 38ul of a cocktail of antibodies **(Table S3)** and incubated for 30 minutes at room temperature. After staining, a 2% paraformaldehyde (PFA) solution in phosphate buffered saline (PBS) solution was added to the blood aliquot to achieve a final concentration of 0.5% PFA. Samples were incubated for 5 minutes at room temperature, and then 350ul of Smart Tube stabilizer (Fisher Scientific) was added and incubated for 10 minutes, and then frozen at -80°C until data acquisition. In preparation for mass cytometry cell labelling, all frozen samples from an individual participant were thawed in parallel, with the cells re-suspended in complete RPMI medium (Gibco, Life Technologies) supplemented with 10% heat-inactivated fetal bovine serum (FBS, Institut de Biotechnologies Jacques Boy), and then washed in CSM-S buffer (PBS supplemented with 5 mg/mL BSA and 0.3% saponin (Sigma-Aldrich)). Barcoding of participant samples by visit was performed by washing cells in PBS and 0.3% saponin (Sigma-Aldrich), then labelled with either 106 or 108 palladium isotopes chelated to isothiocyano-benzyl-EDTA (final concentration of 200 nM) (*3*). Following 30 minutes incubation at 4 °C, cells were re-suspended in PBS supplemented with 10% FBS, washed with CSM-S then D0 and D1 samples from an individual participant where pooled. Next, cells were washed and incubated for 30 min at RT with a 100-µl cocktail of metal-conjugated antibodies. Cells were washed and fixed for 10 min at RT with 2.4% paraformaldehyde (Sigma-Aldrich). Total cells were identified by DNA intercalation (1 µM Cell-ID intercalator; Fluidigm/DVS Science) in 2% PFA at 4°C overnight. Labelled samples were assessed by using a Helios mass cytometer instrument (Fluidigm), using a flow rate of 0.030 ml/min and an event rate of ~300 cells/second. Flow cytometry standard (FCS) files were normalized to EQ Four Element calibration beads using CyTOF software.

Single-cell RNA sequencing from whole blood

Single cell sorting of immune cells

In order to isolate cells for single-cell RNA sequencing, up to 4ml of whole blood was treated with in-house prepared ammonium-chloride-potassium red blood cell lysis buffer for 15 minutes at RT. Cells were washed with PBS and stained with a solution of PBS + 2mM EDTA (hereafter referred to as PBS-EDTA) containing fixable viability dye (FVD) blue (Thermo Fisher) at RT, and then washed again. Cells were then stained with a mixture of fluorescently labelled antibodies **(Table S6)** for 30 minutes at RT, washed with PBS-EDTA, resuspended in FACS buffer (PBS + 0.2% bovine serum albumin + 0.09% sodium azide) with 2mM EDTA (Sigma), filtered through a 30µm pore size mesh cell strainer (Sysmex) and kept on ice. Cells were analyzed and sorted on a FACS Aria II cell sorter (BD Bioscience) using FACSDiva software version 7 (BD Bioscience). In order to simultaneously profile the heterogeneity of the APC compartment and to ensure that sufficient cell numbers of were analysed, we applied a broad gating strategy to identify CD14+ CD16- monocytes, CD14-/+ CD16+ monocytes, myeloid and pDCs **(fig. S13A)** and sorted a fixed number of cells per population for each blood sample for RNA sequencing. Cells from different populations were evenly distributed across plates. Single innate immune cells were index-sorted into 384-well plates containing 5µl of mineral oil with unique primers (details below) in each well for single-cell RNA library preparation. Plates were then centrifuged, snap frozen on dry ice and stored at -80°C until library preparation.

Single-cell RNA library preparation

For single-cell RNA sequencing we applied the SORTseq protocol (*4*), an alternative CEL-Seq2 protocol (*5*), with minor modifications (listed here). Plates were processed in two batches and plates from the same participant were processed in parallel. Before processing, frozen plates were centrifuged at 1000 RPM for 2 minutes at 4oC. To dispense the reagents of the lysis step, RT-reaction, and the second-strand synthesis, we used the Nanodrop II

micro-dispenser (BioNex). In the first-strand reaction of library preparation, we applied random octamer primers that contain part of T7 sequence of Illumina library oligos. cDNA library was amplified and enriched by 7 cycles of PCR using KAPA hyper prep kit and adding forward and reverse primers against Illumine T5 and T7 sequences in which the reverse primer contained the unique index sequence. Each library obtained from one plate was sequenced for on average 30 million reads using NextSeq 500 sequencer.

Single-cell RNA sequencing data analysis

A custom in-house script was used to map reads, demultiplex transcripts per cell, and count transcripts per cell for downstream analysis. Raw FASTQ files (Read1 contains the 8-nucleotide barcode + 8nt UMI and Read2 contains the mRNA sequence) for each plate were mapped to the human transcriptome (HG38) using the STAR aligner (*1*). Uniquely mapped reads were demultiplexed per cell using the known cell barcodes, of which the mapping to specific wells in the 384-well plate is known. Unique RNA molecules were counted and summed per gene using the UMI sequence. Finally, a cell x gene matrix (N = 10,655 cells in total) was built with the Seurat v3 R package (*6*). Low-quality cells were filtered from the dataset if they were >3 median-absolute deviations for ERCC counts, mitochondrial counts, library size using the quickPerCellQC() function of scater R package (*7*). Multivariate outliers were also removed using the same quality-control variables. Cells were filtered for total detected genes per sorted population (CD14+ CD16- monocytes: less than 250 genes or more than 5000; CD14-/+ CD16+ monocytes, myeloid, and pDCs: less than 750 genes or more than 7000). After filtering, we obtained N = 6348 high quality cells in total. Mitochondrial and ribosomal genes were removed from the dataset for downstream analysis. Gene expression data was normalized using the SCTransform() function of Seurat v3 which was also used to regress the sample batch. Lastly, the RunPCA() function of

Seurat v3 was used to calculate the principal components of the dataset, of which the top 30 were retained for downstream analysis.

Processing of single-cell FACS data

Single-cell index sorting provides a FCS file for each 384-well plate that is sorted, which contains marker expression values that are mapped to each well of the plate. The R package flowCore (*8*) was used to perform the data handling steps (demultiplexing and annotation of cells) before integration with single cell RNA sequencing data. Marker expression values were bi-exponential transformed with the estimateLogicle() function of flowCore. Since the mapping of each cell to each well is known, marker expression values were mapped to the transcriptome of each individual cell and then stored with matched single-cell RNA sequencing data using Seurat v3.

Phospho-signaling analysis of innate immune cells

Peripheral blood mononuclear cells (PBMCs) were isolated from fresh blood samples of healthy donors using the Ficoll Paque™ PLUS (GE Healthcare) density gradient media according to the manufacturer's protocol. PBMCs suspended in complete RPMI medium (Gibco, Life Technologies) (10% heat-inactivated fetal bovine serum [FBS] [Institut de Biotechnologies Jacques Boy] and stimulated with either PBS control, Alum, Tdap vaccine, Tdap-IPV vaccine, or IPV vaccine at a 1:5 dilution, and then incubated for 15 minutes at 37 °C, fixed with Smart Tube stabilizer (Fisher Scientific) and frozen according to the manufacturer's protocol. Where the effect of bafilomycin A1 was examined, PBMCs were pre-treated with Bafilomycin A1 (InvivoGen; tlrl-baf1, 1 µM) or with PBS for 30 minutes at 37 °C prior prior to stimulation with vaccines and sample processing as indicated above. In preparation for mass cytometry cell labeling, all frozen samples from an individual donor were thawed in parallel with cells re-suspended in complete RPMI medium and then washed

in CSM-S buffer (PBS supplemented with 5 mg/mL BSA and 0.3% saponin (Sigma-Aldrich)). Barcoding of donor samples by treatment condition was performed by incubating cells with one of five different palladium isotopes chelated to isothiocyano-benzyl-EDTA (final concentration of 200 nM). Following a 30 minute incubation at 4 °C, cells were re-suspended in PBS supplemented with 10% FBS, washed with CSM-S then all donor samples where pooled. Next, cells washed in PBS and 0.3% saponin (Sigma-Aldrich), incubated for 30 min at RT with a 100-µl cocktail of metal-conjugated antibodies then fixed for 10 min at RT with 2.4% paraformaldehyde (Sigma-Aldrich). Total cells were identified by DNA intercalation (1 µM Cell-ID intercalator; Fluidigm/DVS Science) in 2% PFA at 4°C overnight. Labeled samples were assessed by using a Helios mass cytometer instrument (Fluidigm), using a flow rate of 0.030 ml/min and an event rate of ~300 cells/second. Flow cytometry standard (FCS) files were normalized to EQ Four Element calibration beads using CyTOF software. For conventional cytometric analysis of immune cell populations, FCS files were imported into Cytobank data analysis software (v7.3.0).

Bioinformatic analyses

Gene set enrichment analysis

Enriched gene sets were identified using gene set enrichment analysis (GSEA, (*9*)) using the pre-ranked list of genes mode. GSEA was calculated using the ClusterProfileR R package *(10)* using 10,000 permutations. To identify differential pathways in whole blood post-vaccination, raw genes were counts per million normalized and then ranked by their log2-fold change (D1/D0) and assessed with a gene set list of blood transcriptomic modules (BTMs, (*11*)) from the 'tmod' R package(*12*). All pathways with FDR < 0.05 are presented (**Fig. 2b** and **fig. S5**). To identify BTMs that are associated with antibody responses per cohort, changes in antibody responses (D28 or Y1 / D0) were correlated with changes in expression (D1 / D0) for each gene. GSEA using BTMs was applied to a pre-ranked gene

list in which each gene is ranked by its correlation with antibody responses. The BTMs with more than two FDR-adjusted significant associations (FDR < 0.05) with antibody responses are shown (**Fig. S8**). To identify BTMs that are associated with antibody responses and are shared between the UK and NL cohorts, we combined data from the NL and UK cohorts. We normalised gene expression changes and adjusted antibody responses per cohort by subtracting the mean and dividing by the standard deviation for each gene or antibody response. Thereafter, the gene expression and antibody response matrices from the UK and NL cohorts were concatenated to calculate correlations and subsequently, GSEA on the ranked gene list as described above. BTMs with more than two FDR-adjusted significant associations (FDR < 0.05) with antibody responses are shown (**Fig. 3**). To identify differential pathways in single-cell pseudobulk RNA samples, genes were ranked by their log2-fold change (D1 / D0) and assessed with BTMS or the Gene Ontology (GO) terms gene set list.

Comparison with external Tdap vaccination data from da Silva Antunes et al. (2021)

Transcriptomic and antibody data were downloaded from the following URLs: https://www.ncbi.nlm.nih.gov/geo/query/acc.cgi?acc=GSE152683, https://insight.jci.org/articles/view/141023/sd/4. Low-quality RNAseq samples were identified at the URL: https://github.com/JasonPBennett/Pertussis_2020/blob/main/Pertussis_analysis_rnaseq.ipynb and excluded from downstream analysis. Similar to the present study, genes with a biotype annotated as “protein coding” were used for downstream analysis. In addition, we used genes that were present in the count matrix of both the present study and the da Silva Antunes et al. study for better comparability between studies. This process restricted the total number of genes from N = 19190 protein coding genes to N = 19166 protein coding genes present in both studies. Differential gene expression analysis of pre- (D0) and post-

vaccination (D1) samples was performed similarly to the analysis of whole blood transcriptomes in the present study. We used DESeq2 with the raw read count x sample matrix, which identified in total N = 714 DEGs (a DEG was defined as FDR < 0.05 and |log2 fold change| > 0.5). In order to compare post-vaccination Tdap-IPV and Tdap gene expression, we performed GSEA using BTMs on a list of all genes ranked by the difference in log2-fold change in expression between Tdap-IPV and Tdap vaccines. Formally, the calculation was performed as: $\text{Difference}_{gene.x} = \text{Log2}(D1 / D0)_{Tdap\text{-}IPV,\ gene.x} – \text{Log2}(D1 / D0)_{Tdap,\ gene.x}$ and the gene list ranked this way places genes that have higher expression in Tdap-IPV condition at the top of the list. All enriched BTMs are shown in **Fig. 7B**. Antibody concentration values were log10 transformed and antibody responses were calculated as log10 fold changes (D30 or D90 / D0). Concentrations and responses were compared with a paired Wilcoxon test **(Fig. 17A-C)**. In order to identify BTMs that are associated with antibody responses, similar to whole blood analysis in the present study, changes in antibody responses (D30 or D90 / D0) were correlated with log2-fold changes (D1 / D0) changes in CPM-normalized gene expression. GSEA using BTMs was applied to a pre-ranked gene list in which each gene is ranked by its correlation with antibody responses **(Fig. 7D)**.

References

1. A. Dobin *et al.*, STAR: ultrafast universal RNA-seq aligner. *Bioinformatics* **29**, 15-21 (2013).
2. Y. Liao, G. K. Smyth, W. Shi, featureCounts: an efficient general purpose program for assigning sequence reads to genomic features. *Bioinformatics* **30**, 923-930 (2014).
3. E. R. Zunder *et al.*, Palladium-based mass tag cell barcoding with a doublet-filtering scheme and single-cell deconvolution algorithm. *Nat Protoc* **10**, 316-333 (2015).
4. M. J. Muraro *et al.*, A Single-Cell Transcriptome Atlas of the Human Pancreas. *Cell Syst* **3**, 385-394 e383 (2016).
5. T. Hashimshony *et al.*, CEL-Seq2: sensitive highly-multiplexed single-cell RNA-Seq. *Genome Biol* **17**, 77 (2016).
6. T. Stuart *et al.*, Comprehensive Integration of Single-Cell Data. *Cell* **177**, 1888-1902 e1821 (2019).
7. D. J. McCarthy, K. R. Campbell, A. T. Lun, Q. F. Wills, Scater: pre-processing, quality control, normalization and visualization of single-cell RNA-seq data in R. *Bioinformatics* **33**, 1179-1186 (2017).
8. F. Hahne *et al.*, flowCore: a Bioconductor package for high throughput flow cytometry. *BMC Bioinformatics* **10**, 106 (2009).
9. A. Subramanian *et al.*, Gene set enrichment analysis: a knowledge-based approach for interpreting genome-wide expression profiles. *Proc Natl Acad Sci U S A* **102**, 15545-15550 (2005).
10. G. Yu, L. G. Wang, Y. Han, Q. Y. He, clusterProfiler: an R package for comparing biological themes among gene clusters. *OMICS* **16**, 284-287 (2012).
11. S. Li *et al.*, Molecular signatures of antibody responses derived from a systems biology study of five human vaccines. *Nature immunology* **15**, 195-204 (2014).
12. J. Zyla *et al.*, Gene set enrichment for reproducible science: comparison of CERNO and eight other algorithms. *Bioinformatics* **35**, 5146-5154 (2019).

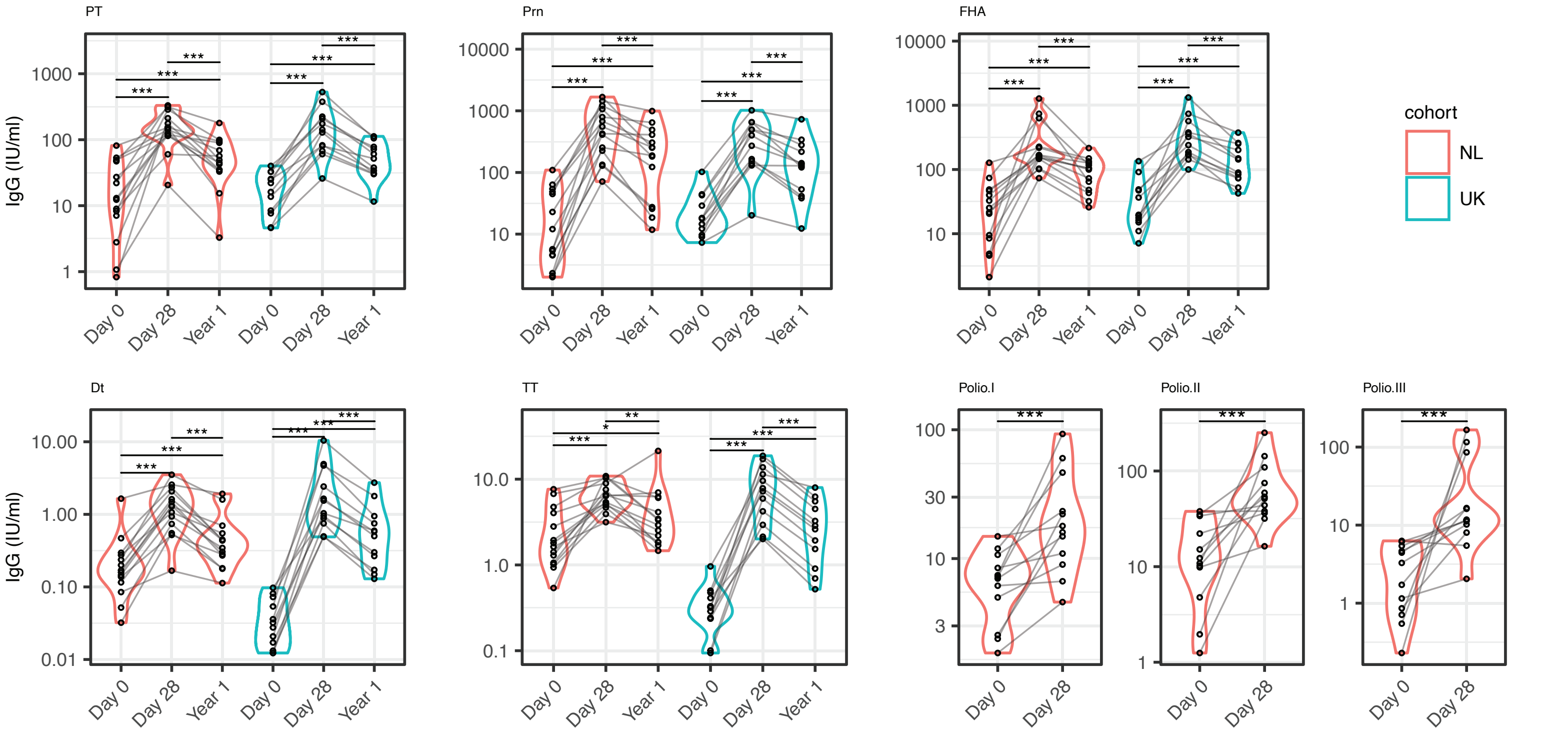

Figure S1. Serum antibody concentrations induced by Tdap-IPV vaccination and comparison of antibody responses. IgG concentrations (International Units, IU) are shown at baseline of Tdap-IPV vaccination (Day 0), 28 days. (Day 28), and one year (Year 1) post Tdap-IPV vaccination. IgG concentration values for the specified Tdap-IPV antigens are shown on the log10 scale. Data are N = 14 for the Netherlands (NL) cohort and N = 12 for the United Kingdom (UK) cohort. Significance (nominal p.value) was calculated with a two-sided paired Wilcoxon test, * p <0.05; ** p <0.01; *** p <0.001. Abbreviations: FHA, filamentous haemagglutinin; Prn, pertactin; PT, pertussis toxin; Dt, diphtheria toxoid; TT, tetanus toxin. Source data are provided in the Source Data file.

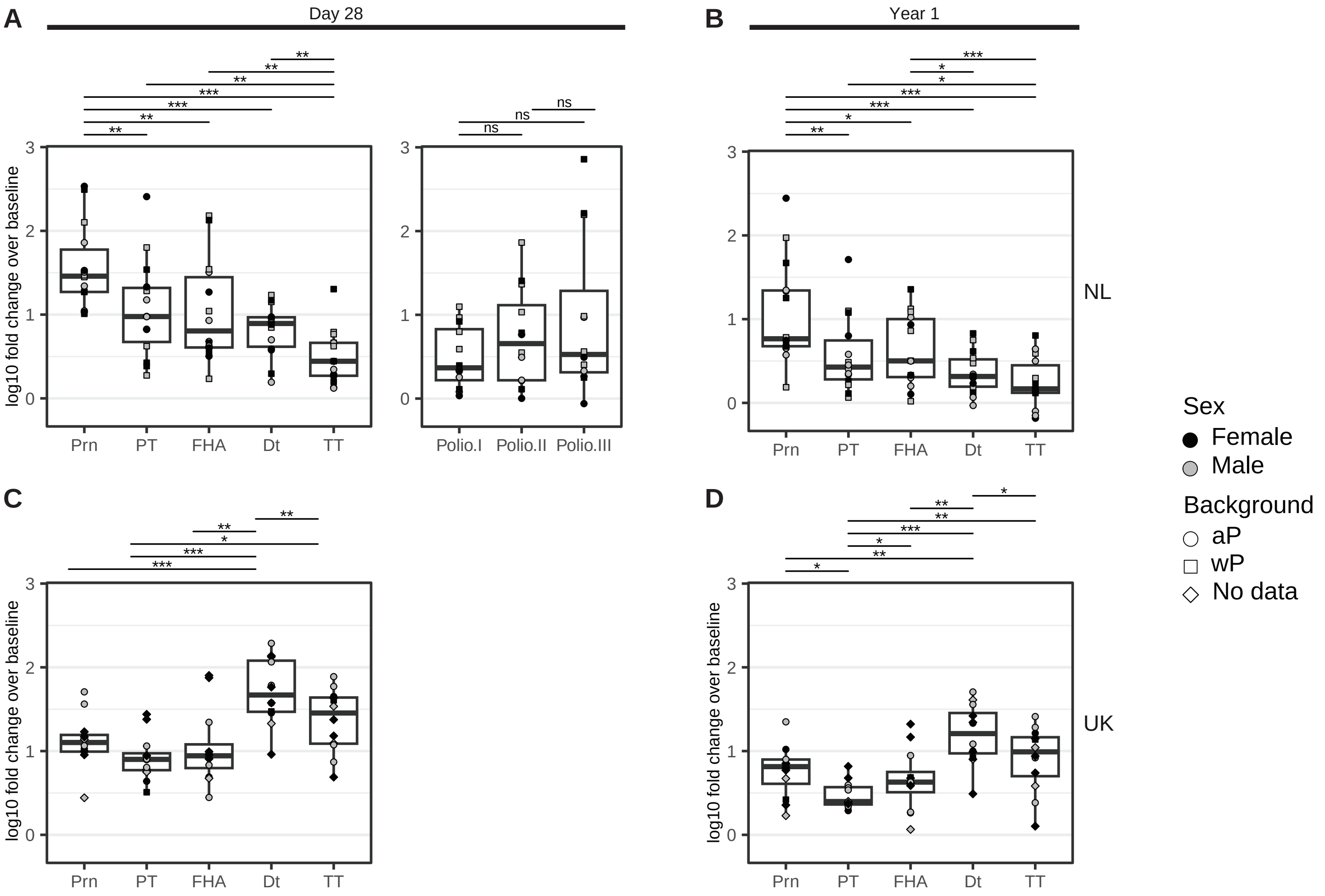

Figure S2. Post-vaccination Tdap-IPV antibody responses. Antibody responses are shown (log10 fold change of IgG concentrations over baseline) for the specified Tdap-IPV antigens at 28 days (Day 28 / Day 0) and one year (Year 1 / Day 0) post-vaccination in (**A-B**) the Netherlands (NL) and (**C-D**) United Kingdom (UK) cohorts. Data are N = 14 for the NL cohort and N = 12 in the UK cohort and are represented as a box plots, with bounds from 25th to 75th percentile, median line, and whiskers, which extend to the largest or smallest value no further than 1.5 * the inter-quartile range. Statistical significance (nominal p.value) was determined using a two-sided paired Wilcoxon test, * p <0.05; ** p <0.01; *** p <0.001. Source data are provided in the Source Data file.

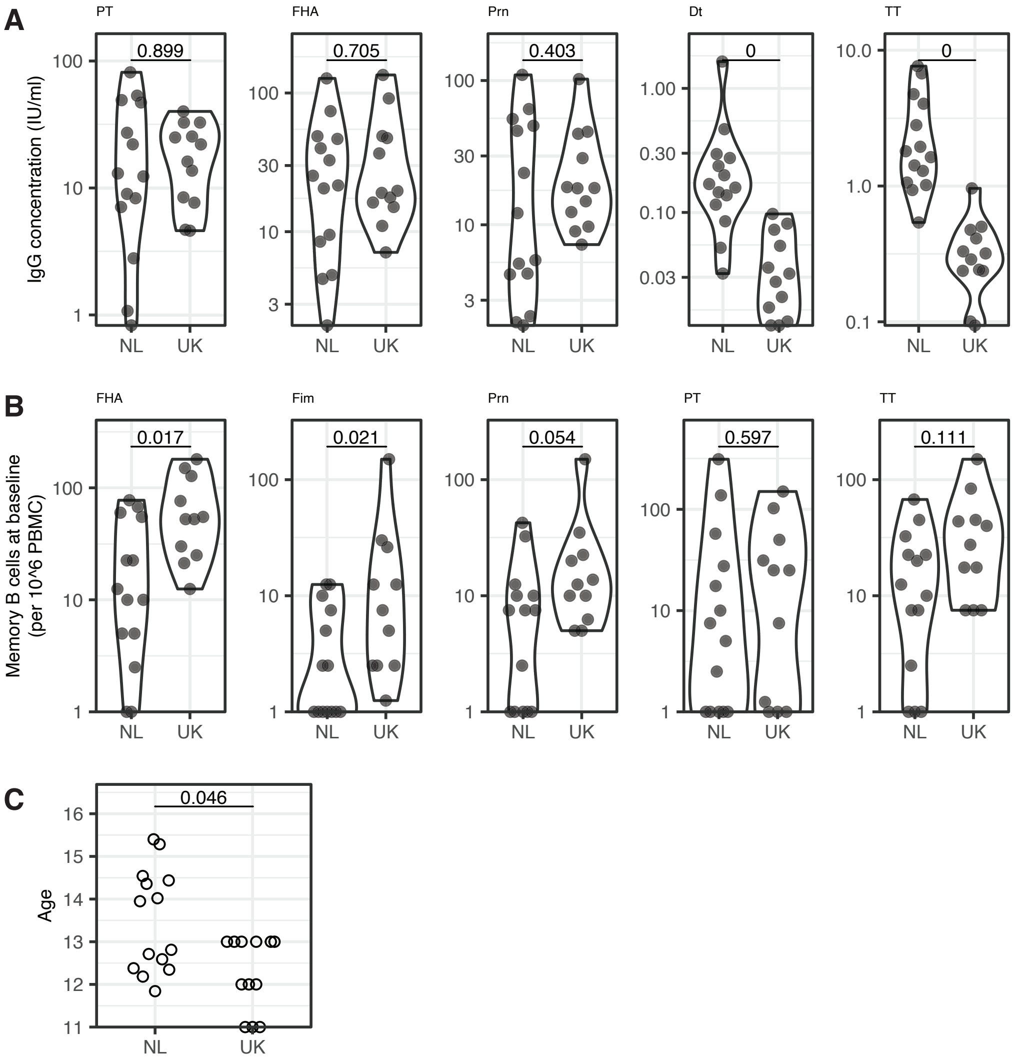

Figure S3. Baseline characteristics of the Netherlands and United Kingdom cohorts. (**A**) The number of pre-vaccination memory B cells that are specific for the specified antigens (FHA, Fim, Prn, PT, and TT) was quantified in PBMCs with Elispot. Data are N = 14 participants in the Netherlands (NL) cohort and N = 11 participants in the United Kingdom (UK) cohort. (**B**) The age of participants is shown for the NL (N = 14) and UK (N = 12) cohorts. (**C**) Baseline antibody levels are shown for the specified antigens for participants in the NL (N = 14) and UK (N = 12) cohorts. Data of (A) and (B) are shown on the log10 scale and significance (nominal p.value) was calculated with a two-sided Wilcoxon test. Abbreviations: FHA, filamentous haemagglutinin; Prn, pertactin; PT, pertussis toxin; Fim, fimbriae; Dt, diphtheria toxoid; TT, tetanus toxin. Source data are provided in the Source Data file.

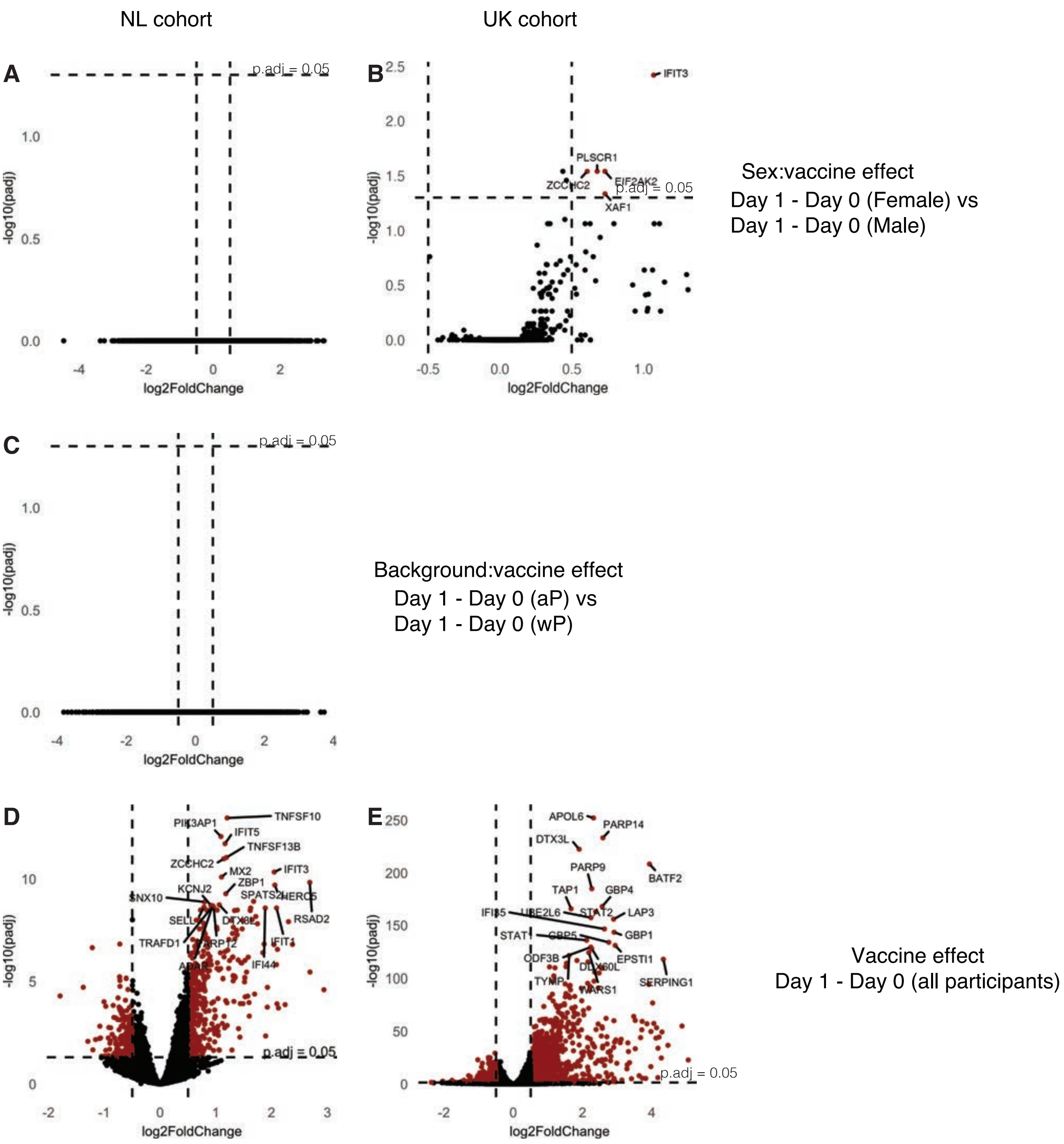

Figure S4. Differential gene expression of blood transcriptional responses one day post Tdap-IPV vaccination. Volcano plot showing statistical significance (false-discovery rate (FDR) adjusted p.value, -log2(padj)) and log2 fold change of genes after comparing Day 1 vs Day 0 transcriptional responses between male and female participants in the (**A**) Netherlands (NL) or (**B**) United Kingdom (UK) cohorts. (**C**) Volcano plot showing statistical significance and log2 fold change of genes after comparing aP and wP priming backgrounds in the NL cohort. (**D-E**) Volcano plots showing statistical significance of genes comparing Day 1 vs Day 0 across all participants in either the NL or UK cohorts. Data are 26 paired samples from N = 13 participants in the NL cohort, and 22 paired samples from N = 11 participants in the UK cohort. Statistical significance was calculated with a negative binomial linear model and false-discovery rate (FDR) adjusted two-sided p.values were calculated. The dotted lines indicated thresholds for differentially expressed genes (DEGs), which highlight 559 DEGs (FDR < 0.05 and I Log2 fold change I > 0.5) in the NL cohort and 1709 DEGs in the UK cohort. The top 20 genes ranked by FDR are labelled. Source data are provided in the Source Data file.

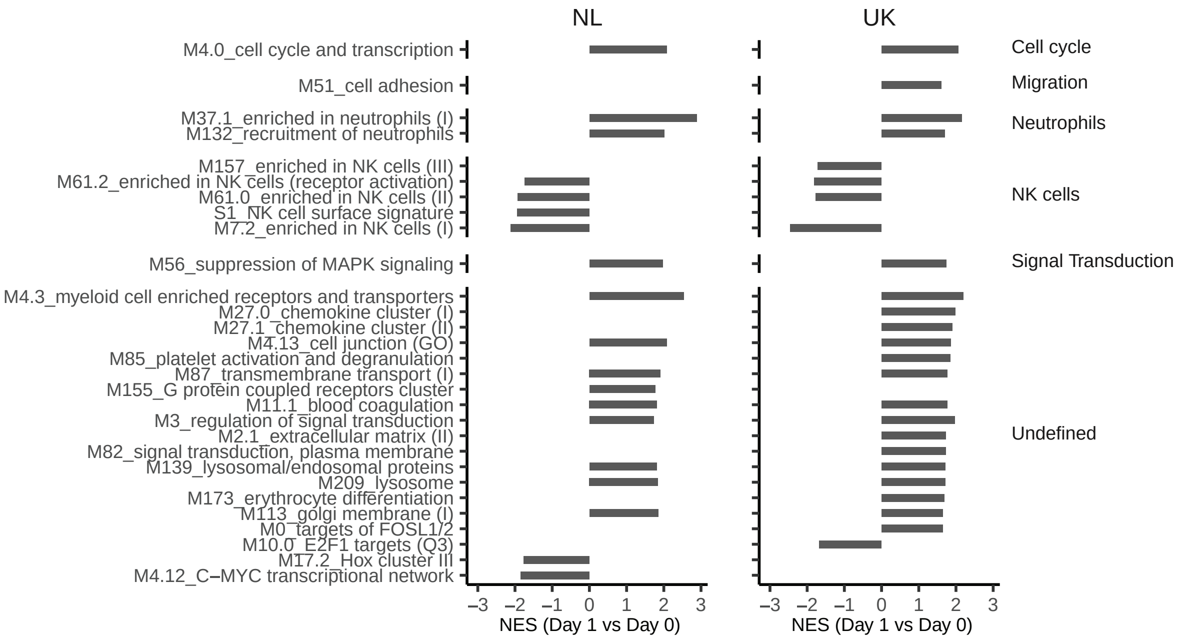

Figure S5. Molecular signatures induced by Tdap-IPV vaccination (continued from Figure 2B). Blood transcription modules (BTMs) enriched (FDR < 0.05) one day after Tdap-IPV vaccination in the Netherlands (NL, left panel) and United Kingdom (UK, right panel) cohorts. Gene set enrichment analysis (GSEA) was used to calculate the normalized enrichment score (NES) of BTMs using a gene list ranked by the log2-fold change of gene expression over baseline (D1 / D0). Statistical significance and p-values were calculated against an empirical null distribution and reflect two-sided tests. False discovery rate (FDR) adjusted p.values were calculated; enriched BTMs (FDR < 0.05) are grouped based on their biological function. Data are N = 26 paired samples from 13 participants in the NL cohort, and N = 22 paired samples from 11 participants in the UK cohort. Source data are provided in the Source Data file.

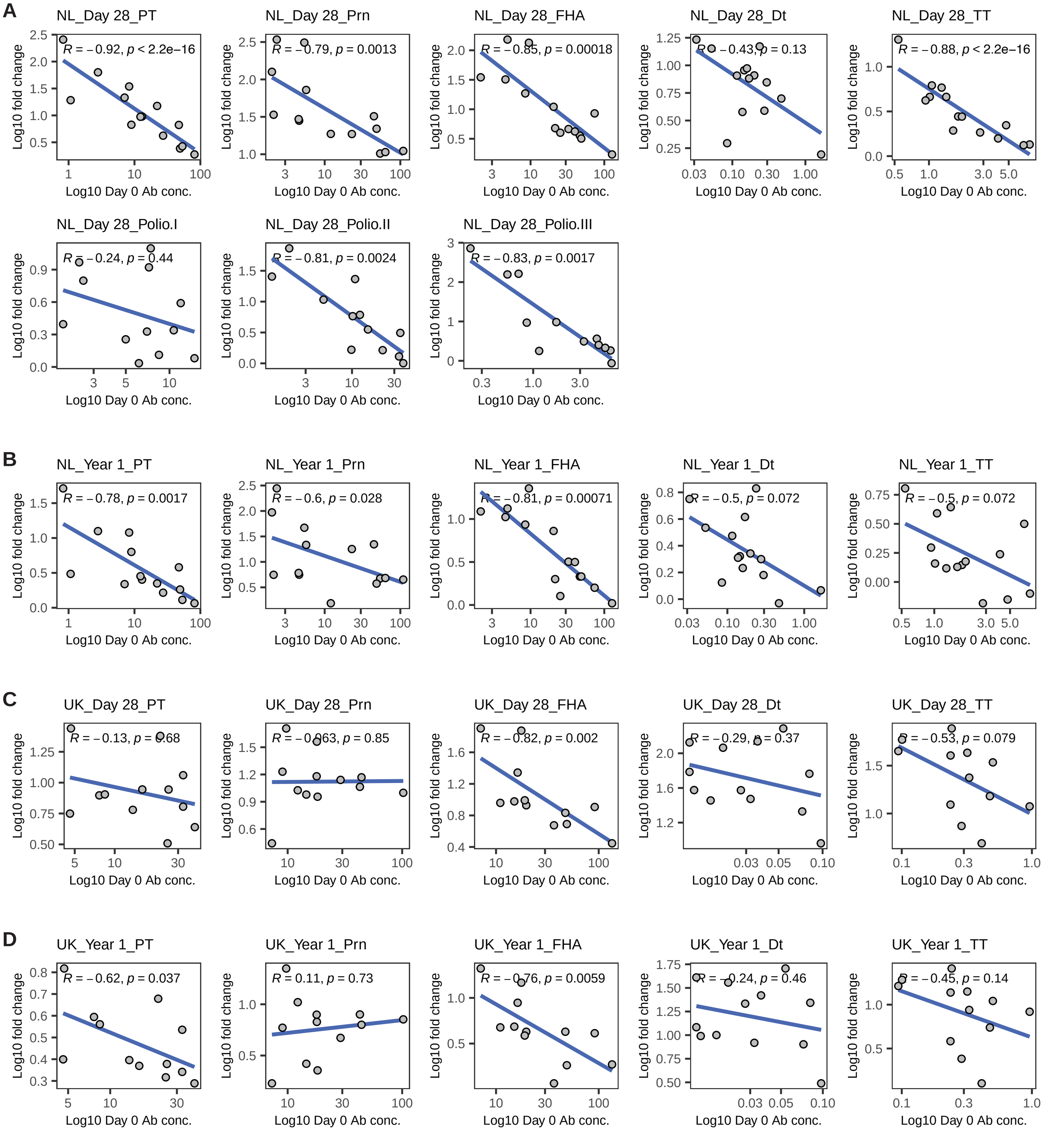

Figure S6. Post-vaccination antibody responses are negatively correlated with baseline antibody levels. For each of the specified antigens, scatterplots of pre-vaccination (Day 0) antibody levels on the x-axis with log10 fold change responses on the y-axis are shown with Spearman correlation coefficient, nominal p.value and linear regression trendline. (**A**) Antibody responses of participants in the Netherlands (NL) cohort at day 28 post-vaccination and (**B**) one year post-vaccination. (**C**) Antibody responses of participants in the United Kingdom (UK) cohort at day 28 post-vaccination and (**D**) one year post-vaccination. Data are N = 14 for the Netherlands (NL) cohort and N = 12 for the United Kingdom (UK) cohort. Abbreviations: FHA, filamentous haemagglutinin; Prn, pertactin; PT, pertussis toxin; Dt, diphtheria toxoid; TT, tetanus toxin. Source data are provided in the Source Data file.

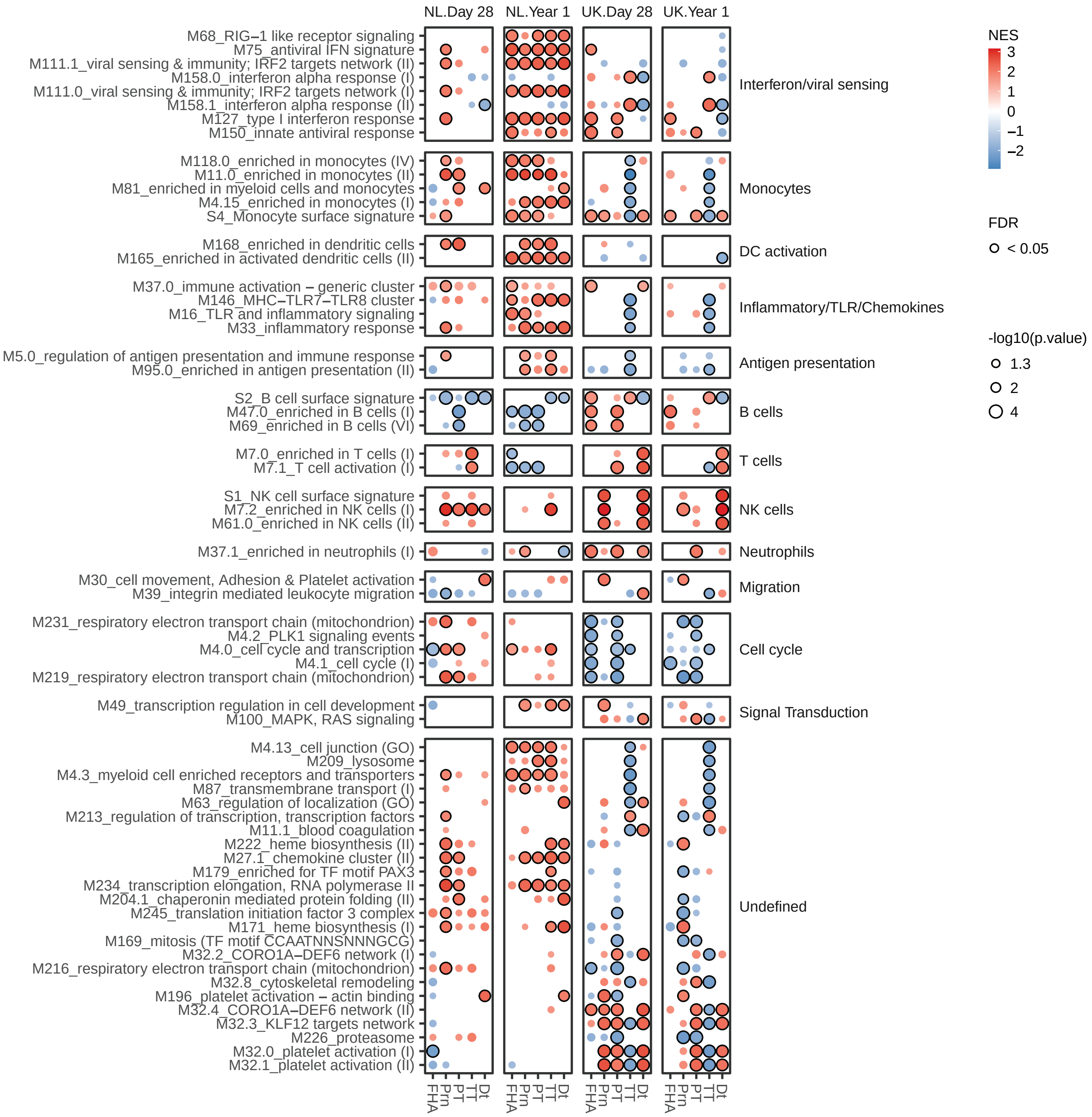

Figure S7. Molecular signatures associated with the adjusted antibody response induced by Tdap-IPV per cohort. Dot plot of blood transcription modules (BTMs, rows) whose activity one day post vaccination (Day 1 / Day 0) is associated with adjusted log10-fold change (Methods) of Tdap-IPV antigen-specific antibody responses (columns) 28 days (Day 28 / Day 0) or one year (Year 1 / Day 0) post vaccination. Data are shown for participants in the Netherlands (NL, 26 paired samples from N = 13 participants) and United Kingdom (UK, 22 paired samples from N = 11 participants) cohorts. Gene set enrichment analysis was used to identify positive (red) or negative (blue) enrichment of BTMs within pre-ranked gene lists, where genes were ordered according to their correlation between gene expression and antibody response. Statistical significance and p-values were calculated against an empirical null distribution and reflect two-sided tests. False discovery rate (FDR) adjusted p.values were calculated. BTMs shown, (nominal p.value < 0.05) have more than two FDR-adjusted significant associations with antibody responses, and enriched BTMs with FDR < 0.05 are highlighted with a black border. BTMs are annotated according to biological function on the right. Abbreviations: NES, normalized enrichment score; FDR, false discovery rate; FHA, filamentous haemagglutinin; PRN, pertactin; PT, pertussis toxin; Dt, diphtheria toxoid; TT, tetanus toxin. Source data are provided in the Source Data file.

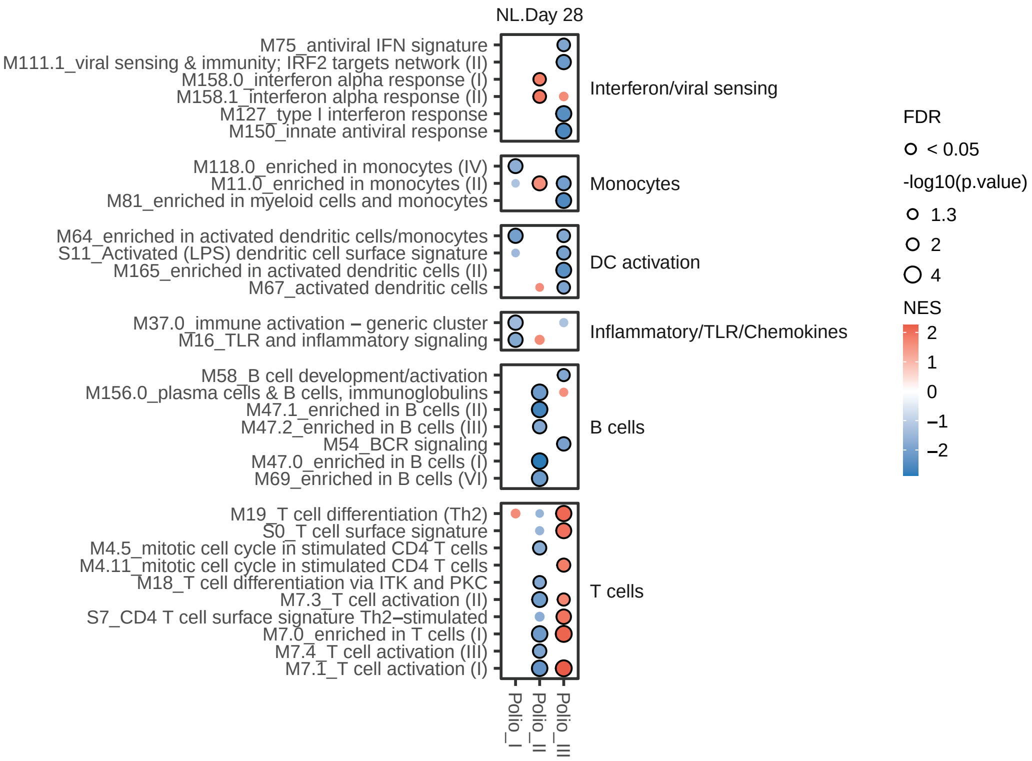

Figure S8. Molecular signatures associated with the adjusted inactivated poliovirus-specific antibody responses induced by Tdap-IPV. Dot plot of blood transcription modules (BTMs, rows) whose activity one day post vaccination (Day 1 / Day 0) is associated with adjusted log10-fold change (Methods) of antibody responses (columns) 28 days (Day 28 / Day 0) post vaccination. Data are shown for participants in the Netherlands (NL, 26 paired samples from N = 13 participants) cohort. Gene set enrichment analysis was used to identify positive (red) or negative (blue) enrichment of BTMs within pre-ranked gene lists, where genes were ordered according to their correlation between gene expression and antibody response. Statistical significance and p-values were calculated against an empirical null distribution and reflect two-sided tests. False discovery rate (FDR) adjusted p.values were calculated. BTMs shown, (nominal p.value < 0.05) have more than one FDR-adjusted significant association with antibody responses, and enriched BTMs with FDR < 0.05 are highlighted with a black border. BTMs are annotated according to biological function on the right. Abbreviations: NES, normalized enrichment score; FDR, false discovery rate. Source data are provided in the Source Data file.

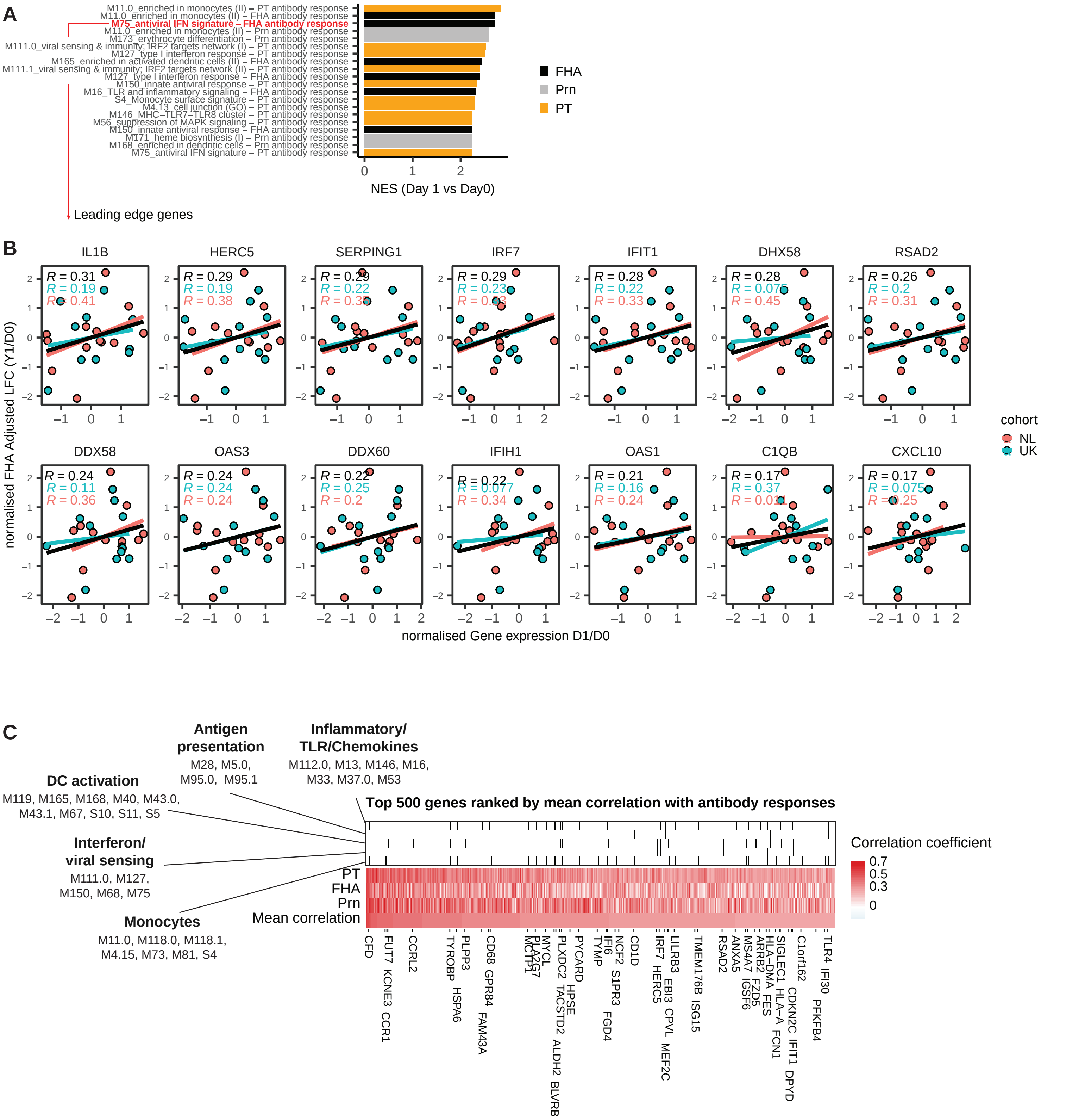

Figure S9. Top BTMs and genes correlating with adjusted one-year antibody responses. (**A**) The top 20 blood transcriptional modules (BTMs) ranked by the normalised enrichment score (NES) with one year adjusted pertussis (PT, FHA, and Prn) antibody responses are shown on the y-axis with the NES on the x-axis. Highlighted in red is the gene set enrichment result of the correlation between the M75_antiviral IFN signature BTM and the FHA antibody response. (**B**) Scatterplots are shown for leading edge genes of the of the highlighted result from (A), depicting the normalised log2-fold gene expression (D1/D0) on the x-axis and the normalised one year FHA adjusted antibody response on the y-axis. In black, the overall Pearson correlation coefficient and regression line are shown together with the Pearson correlation coefficients and regression lines of the UK (blue) and NL (red) cohorts. (**C**) The top 500 genes ranked by the mean Pearson correlation with adjusted antibody responses are shown. Top margin: black lines indicate gene membership for selected BTMs. Below, a heatmap of Pearson correlation coefficients is shown for each gene and adjusted antibody responses for each antigen (left margin) one year (Year 1 / Day 0) post-vaccination. Data are N = 13 participants in the NL and N = 11 participants in the UK cohort. Source data are provided in the Source Data file.

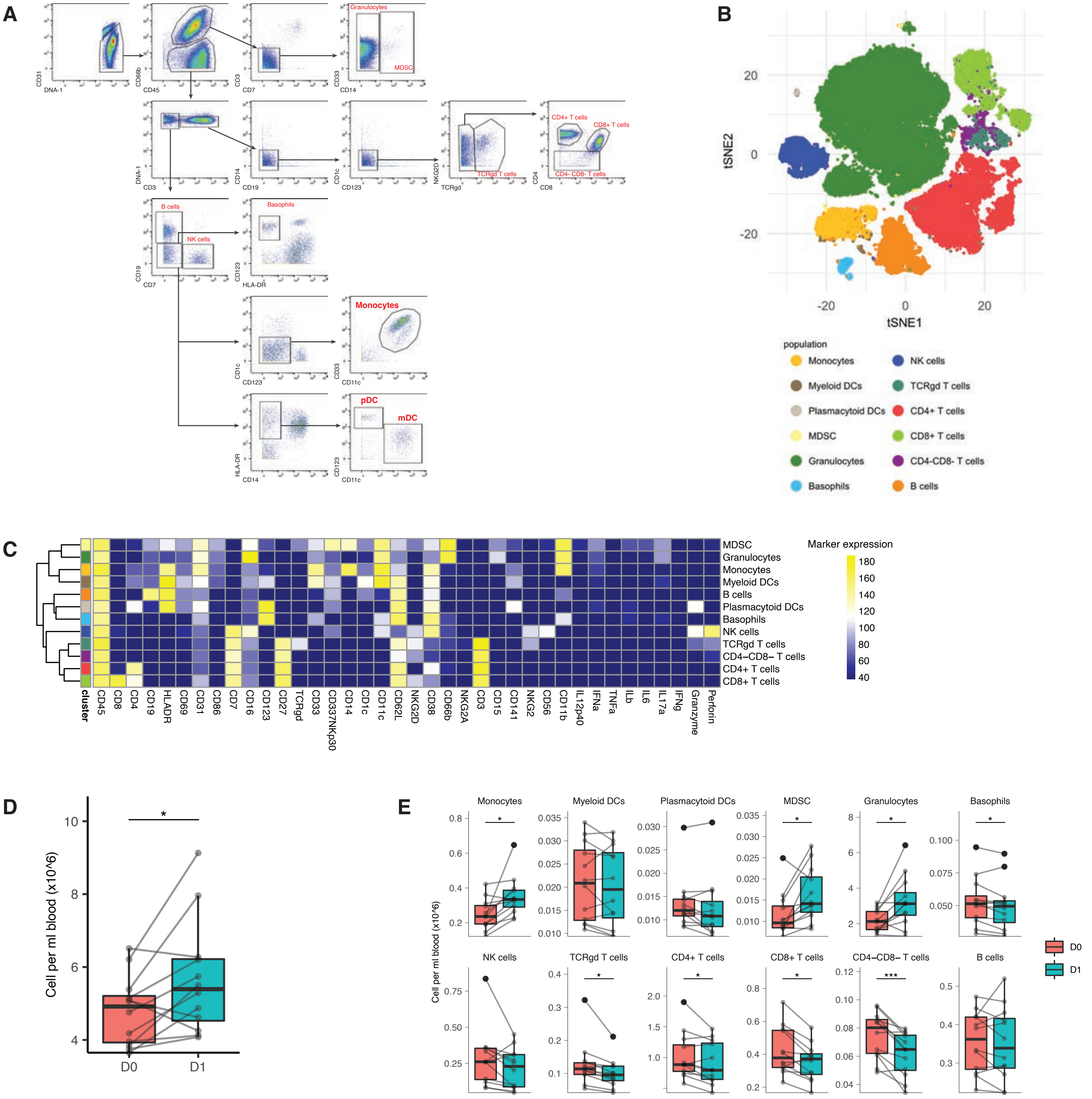

Figure S10. Mass cytometry analysis of circulating immune cells. (**A**) Manual gating of immune cell populations was performed with FlowJo software (TreeStar). Representative bivariate dot plots from a single participant are shown. In total, 12 lineages of immune cells were identified (red font), monocytes, pDC and mDC were extracted for subpopulation analysis (fig. S11). Abbreviations: MDSC, myeloid-derived suppressor cells; NK cells, natural killer cells; pDC, plasmacytoid dendritic cells; mDC, myeloid dendritic cells. (**B**) tSNE visualization of immune cells. Cells are colored according to their respective population, which were identified following manual gating based on phenotypic marker expression. (**C**) Heatmap of median arcsinh marker expression values and hierarchical clustering for the identified populations from (A). (**D**) The total number of circulating leukocytes in blood at Day 0 (D1) and Day 1 (D1). (E) For each population of cells, boxplots representing the number of cells measured in blood from 12 participants before (D0) and 24 hours after vaccination (D1) with Tdap-IPV. Data points represent values for each sample and those from the same participants are joined by a grey line. Data are 24 samples from N = 12 participants in the Netherlands cohort and are represented as a box-and-whisker plots, with bounds from 25th to 75th percentile, median line, and whiskers, which extend to the largest or smallest value no further than 1.5 * the inter-quartile range. Significance (false-discovery rate (FDR) adjusted two-sided p.value) was determined from a mixed-effects regression model for each population, where study day and participant number were specified as fixed and random effects, respectively. * FDR <0.05; ** FDR <0.01; *** FDR <0.001, ns FDR > 0.05. Source data are provided in the Source Data file.

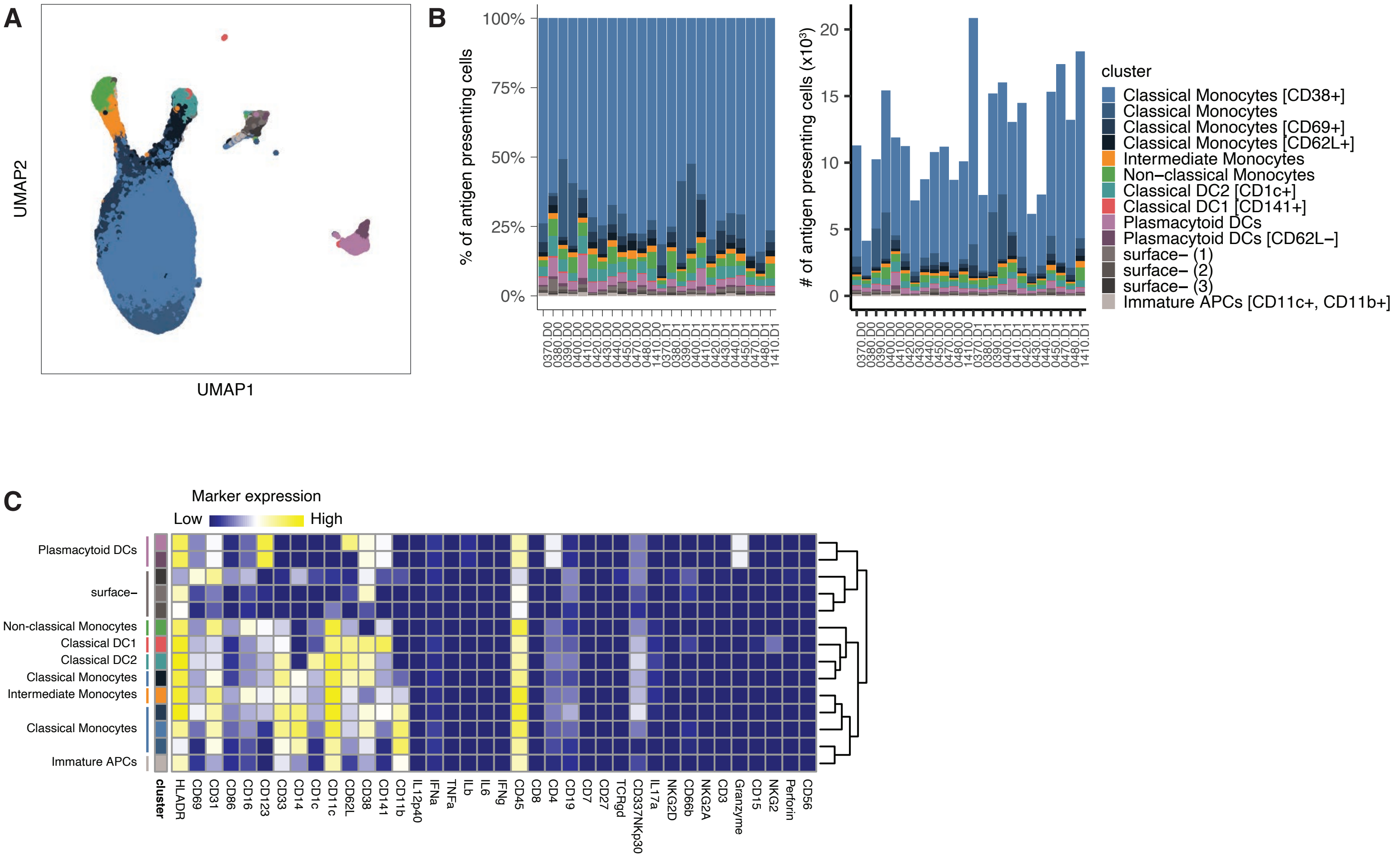

Figure S11. Subpopulation analysis of circulating antigen presenting cells. (**A**) UMAP visualization of antigen presenting cells (APCs, n = ~290,000). Cells are colored according to their cluster, which were identified following clustering with the FlowSOM and ConsensusClusterPlus algorithms. (**B**) The composition of the APC compartment from N = 12 participants of the NL cohort before and 24 hours after vaccination with Tdap-IPV (24 samples total). Each of the twenty-four samples is represented as vertical bars. The lengths of the colored segments show the relative proportion (left panel) or number of cells (right panel) of each cluster. (**C**) Heatmap of median arcsinh marker expression values and hierarchical clustering for the identified clusters indicated on the right-hand margin. Similar clusters were manually merged based on their phenotypic marker expression into subpopulations indicated on the left-hand margin. Source data are provided in the Source Data file.

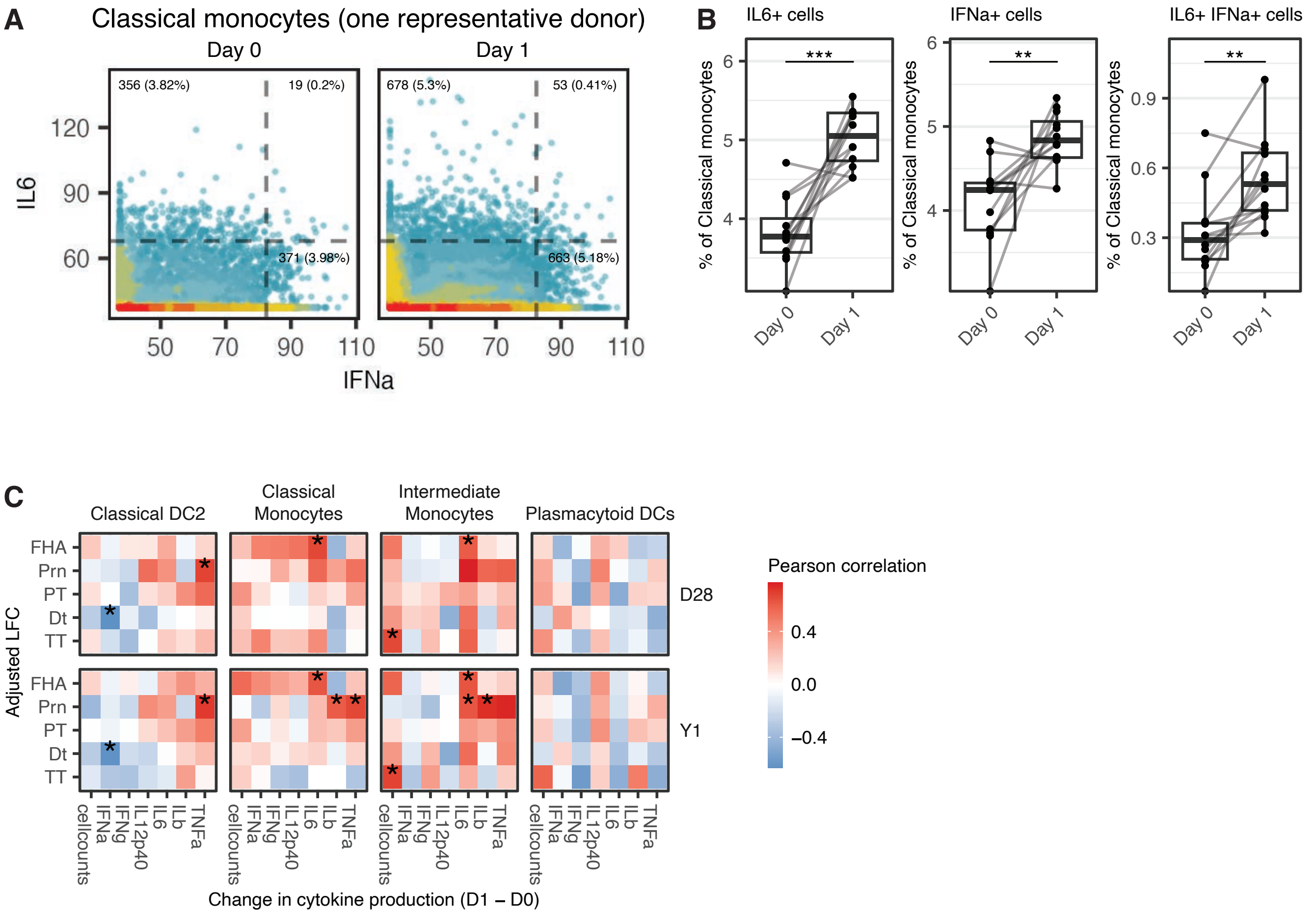

Figure S12. Enhanced interferon-alpha and IL-6 cytokine co-expression in classical monocytes and correlations with adjusted LFC responses. (**A**) Representative bivariate dot plots of IFNa and IL-6 cytokine expression of classical monocytes for one donor at baseline (Day 0) and one day post-vaccination (Day 1). Per donor, classical monocytes from Day 0 and Day 1 were pooled and the 95% quantile was calculated for each cytokine and these values are indicated as dashed lines in the plot. For each timepoint and each donor, cells in each quadrant were counted and the frequency was calculated. These values are indicated in the corners of each quadrant of the plot. (**B**) Boxplots showing the frequency of IFNa+ single positive, IL-6+ single positive, and IL6+IFNa+ double positive cells for each donor and timepoint. Data are 24 samples from N = 12 participants in the Netherlands cohort and those from the same participant are joined by a grey line. Boxplots display bounds from 25th to 75th percentile, median line, and whiskers, which extend to the largest or smallest value no further than 1.5 * the inter-quartile range. Statistical significance (nominal p.value) was calculated with a two-sided Wilcoxon test. ** p <0.01; *** p <0.001. (**C**) Correlations of adjusted log-fold change responses of vaccine antigens (y-axis, top panels: Day 28 responses; bottom panels: 1-year responses) with changes in cell count or cytokine expression of each antigen presenting cell subpopulation (x-axis). Heatmap color indicates Pearson's correlation coefficient. The degree of statistical significance is also shown * nominal two-sided p.value <0.05; data are N = 11 participants in the NL cohort. Abbreviations: FHA, filamentous haemagglutinin; PRN, pertactin; PT, pertussis toxin; Dt, diphtheria toxoid; TT, tetanus toxin. Source data are provided in the Source Data file.

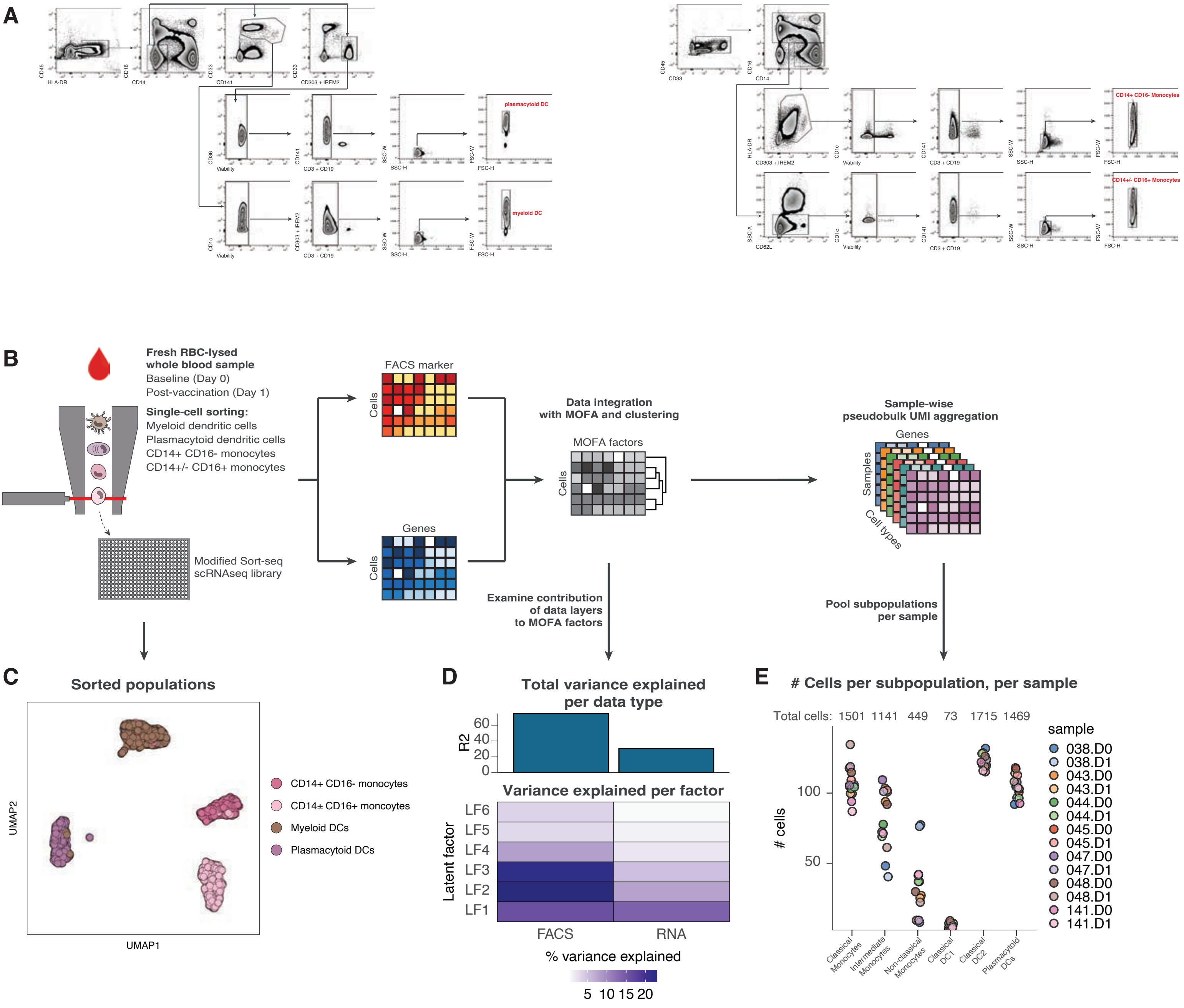

Figure S13. Multi-modal single-cell RNA sequencing analysis of antigen presenting cells. (**A**) Sorting strategy of antigen presenting cell populations for single-cell RNA sequencing. Representative bivariate dot plots from a single participant are shown. Populations marked with red font were single-cell sorted into 384-well microplates. (**B**) Analysis strategy for calculating the effects of Tdap-IPV vaccination on gene expression in antigen presenting cell subpopulations, with overview of sample processing and computational methods. Myeloid dendritic cells, plasmacytoid dendritic cells, CD14+ CD16- monocytes and CD14± CD16+ monocytes were FACS-sorted directly from fresh red blood cell-lysed whole blood samples into 384-well microwell plates for single-cell RNA sequencing. This approach enabled the parallel collection of single-cell RNA and protein (FACS marker) profiles for each cell. Multi-omic factor analysis (MOFA, Argelaguet et al. 2020) was then used to integrate RNA and protein data into a single coordinate space of factors (a cell x MOFA factor graph). Unsupervised clustering was performed on the MOFA graph to identify subpopulations (shown in Figure 5A). A “pseudobulk” sample x gene graph was obtained for each subpopulation by summing gene (unique molecular identifier, UMI) counts of subpopulations in each blood sample. (**C**) UMAP visualization using the MOFA graph of cells labelled by cell-sorting label. (**D**) MOFA analysis identified a total of six latent factors between protein and RNA data layers. Portion of variance explained (R2) for each data layer (top panel) and factor (bottom panel). (**E**) The number of cells obtained for each sample and for each subpopulation. Gene counts for each subpopulation and sample were computationally pooled to generate ‘pseudobulk samples’ for downstream gene expression analysis. For each subpopulation, data are 14 samples representing two timepoints (D1 and D0) from N = 7 participants of the Netherlands cohort. Source data are provided in the Source Data file.

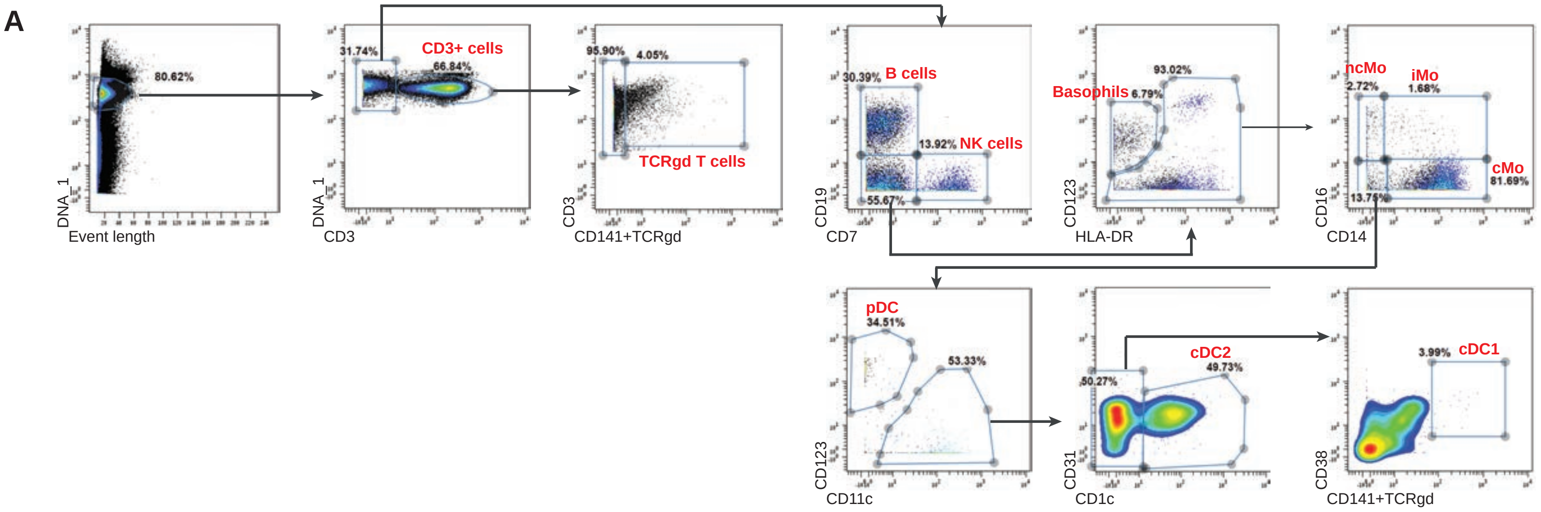

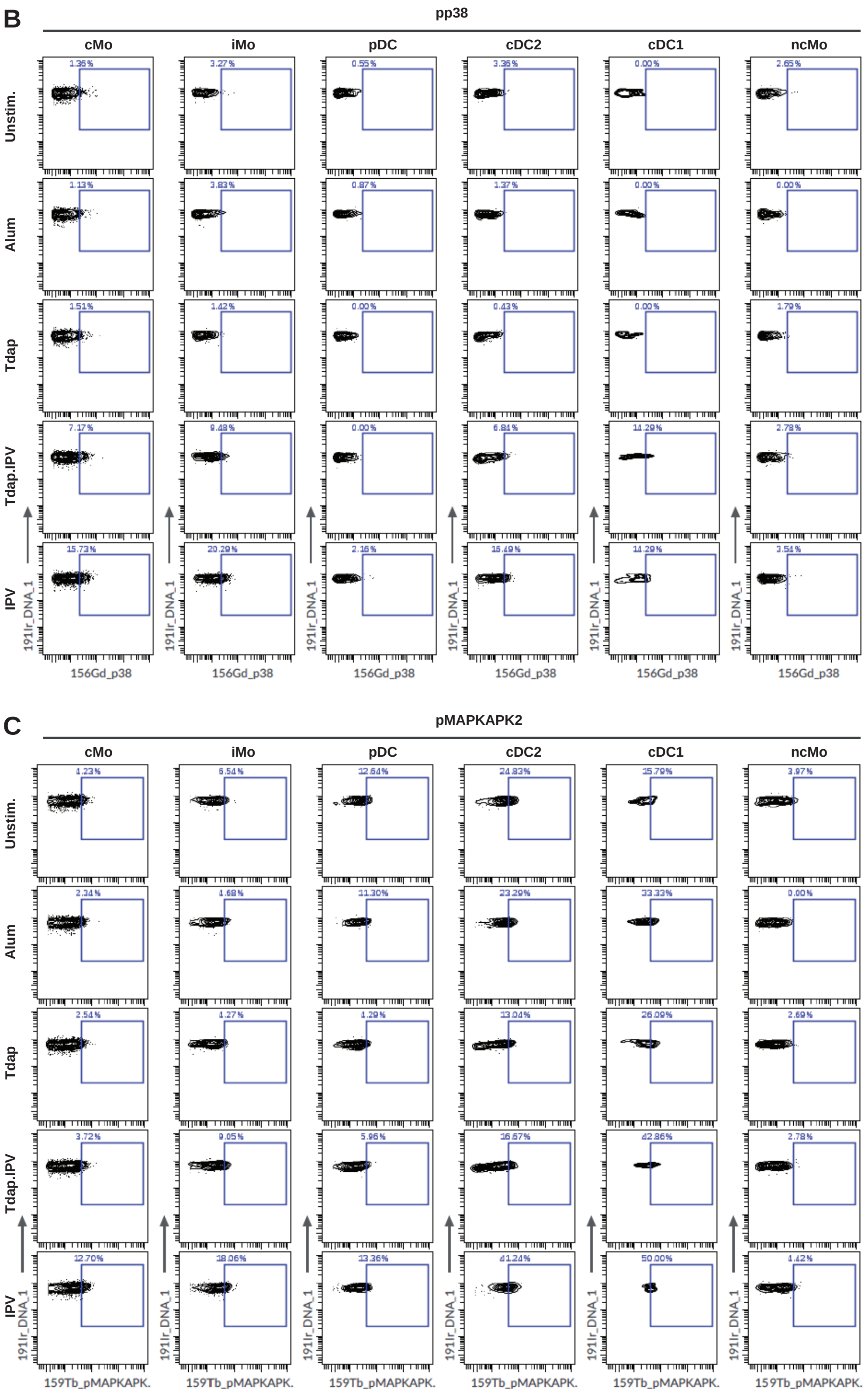

Figure S14. Gating strategy of immune cell populations for analysis of phospho-signaling responses. Representative bivariate dot plots from a single donor are shown. Manual gating was performed with Cytobank software (Beckman Coulter). (**A**) In total,11 lineages of immune cells were identified (red font). (**B**) gating of pp38-positive cells and (**C**) gating of pMAPKAPK2-positive cells in the absence of stimulation (Unstim.) or in response to Alum, Tdap, Tdap-IPV, or IPV stimulation. Data is shown for one representative donor. The following immune cell populations are presented: cMo, iMo, ncMo, pDC, cDC1, cDC2. Abbreviations: NK cells, natural killer cells; cMo, classical monocytes; iMo, intermediate monocytes; ncMo, non-classical monocytes; pDC, plasma-cytoid dendritic cells; cDC1, classical DC1 (CD141+); cDC2, classical DC2 (CD1c+).

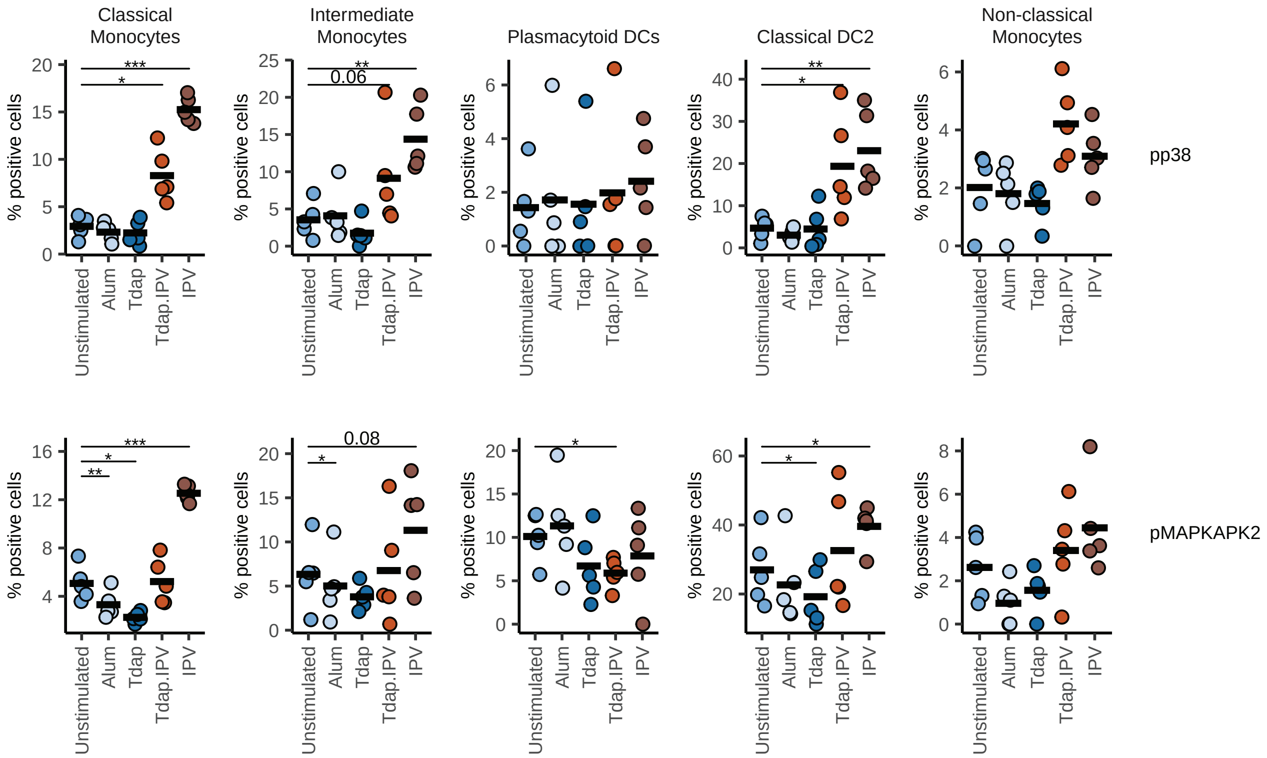

Figure S15. Phospho-signaling responses of innate immune cells in response to Tdap, Tdap-IPV, and IPV stimulation compared to unstimulated cells. The proportion of cells expressing phosphorylated p38 (pp38) or MAPKAPK2 (pMAPKAPK2) in the absence of stimulation (Unstimulated) or in response to Alum, Tdap, Tdap-IPV, or IPV stimulation. Responses of five innate immune cell populations are shown. Data are N = 5 healthy donors, * P < 0.05, ** P < 0.01, *** P < 0.001, statistical significance (nominal p.value) was determined using a two-sided paired T test. Horizontal black lines in each plot correspond to the sample mean. Source data are provided in the Source Data file.

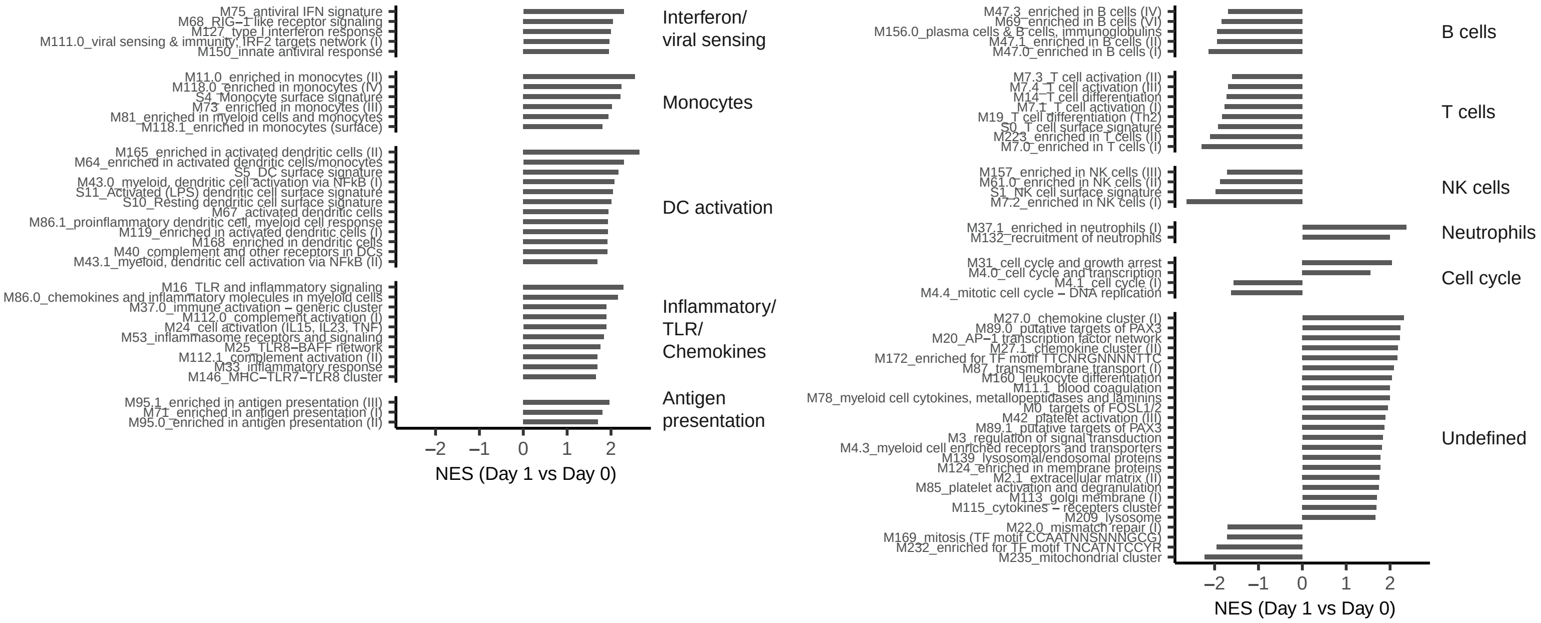

Figure S16. Molecular signatures induced by Tdap vaccination. Publicly available RNA sequencing data derived from PBMCs at baseline (Day 0) and one day Tdap post-vaccination (Day 1) were downloaded (da Silva Antunes et al. JCI Insights 2021) and analyzed. Blood transcription modules (BTMs) enriched (FDR < 0.05) one day after Tdap vaccination are shown. Gene set enrichment analysis (GSEA) was used to calculate the normalized enrichment score (NES) of BTMs using a gene list ranked by the log2-fold change of gene expression over baseline (D1 / D0). Statistical significance and p-values were calculated against an empirical null distribution and reflect two-sided tests. False discovery rate (FDR) adjusted p.values were calculated; enriched BTMs (FDR < 0.05) are grouped based on their biological function. Data are N = 36 participants. Source data are provided in the Source Data file.

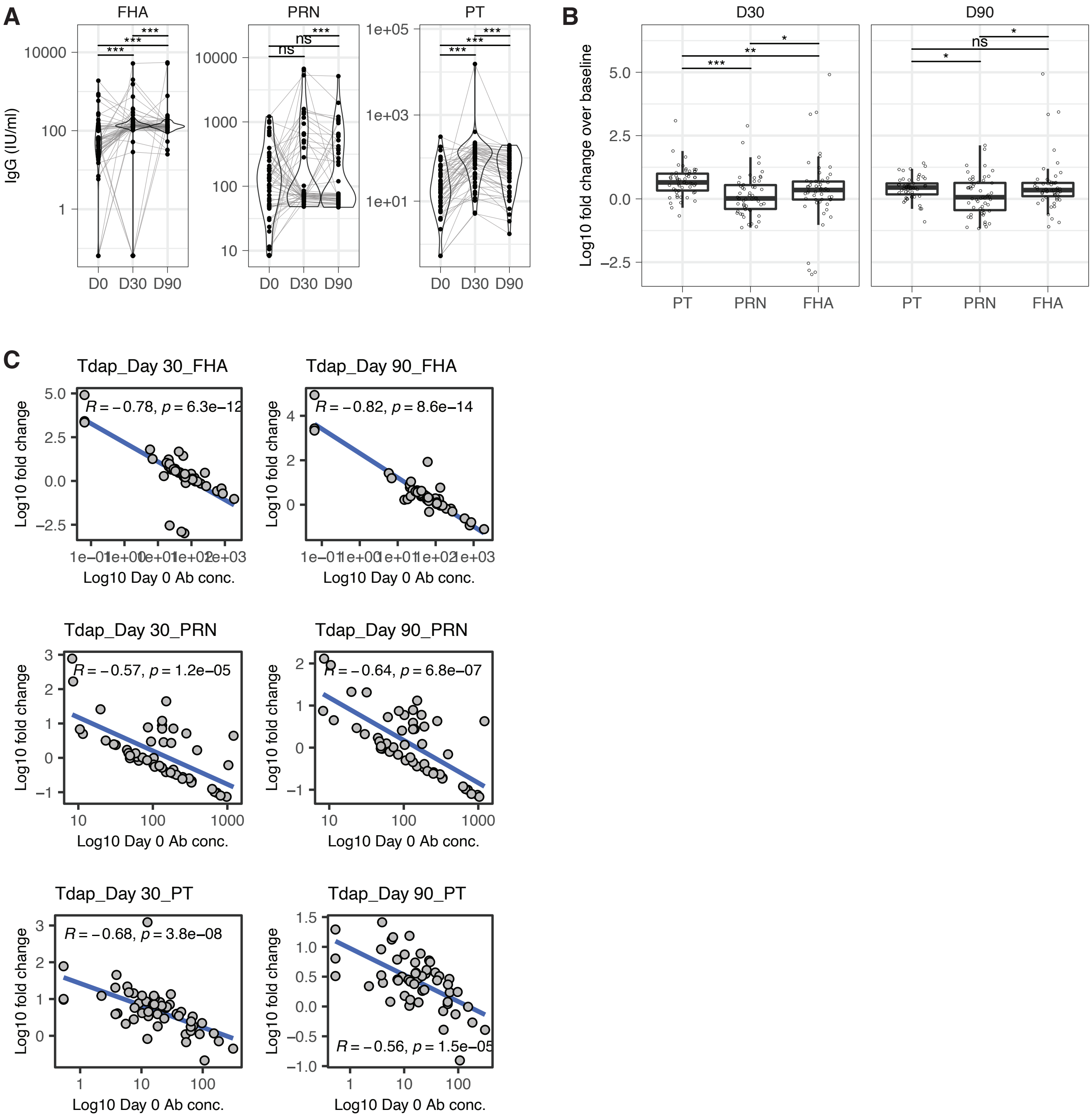

Figure S17. Pre-vaccination and post-vaccination Tdap antibody responses. Publicly available Tdap antibody data for pertussis antigens FHA, Prn, and PT were downloaded (da Silva Antunes et al. JCI Insights 2021). (**A**) IgG concentrations are shown at baseline (D0), at 30 days (D30), or 90 days (D90) post vaccination. (**B**) Box plots displaying the log10 fold change of antibody responses at 30 days (D30 / D0) or 90 days (D90 / D0) post vaccination. Data are N = 58 participants. Box plots display bounds from 25th to 75th percentile, median line, and whiskers, which extend to the largest or smallest value no further than 1.5 * the inter-quartile range. Significance in (A) and (B) was determined using a paired two-sided Wilcoxon test, * $P < 0.05$, ** $P < 0.01$, *** $P < 0.001$, ns $P > 0.05$. (**C**) For each of the specified antigens, scatterplots of pre-vaccination (Day 0) antibody levels on the x-axis with log10 fold change responses on the y-axis are shown with Spearman correlation coefficient, two-sided nominal p.value and linear regression trendline. Abbreviations: FHA, filamentous haemagglutinin; Prn, pertactin; PT, pertussis toxin. Source data are provided in the Source Data file.

Table S1. Participant characteristics and samples used for the various assays

Subject	Cohort	Serum_antbodies	Cytof	ScRNAseq	Sysmex	Blood_RNAseq	age	gender	Background
BK038	NL	D0, D28, Y1 (incl. Polio)	D0, D1	D0, D1	D0, D1		13	M	aP
BK041	NL	D0, D28, Y1 (incl. Polio)	D0, D1		D0, D1	D0, D1	12	M	aP
BK045	NL	D0, D28, Y1 (incl. Polio)	D0, D1	D0, D1	D0, D1	D0, D1	13	M	aP
BK047	NL	D0, D28, Y1 (incl. Polio)	D0, D1	D0, D1	D0, D1	D0, D1	12	F	aP
BK048	NL	D0, D28, Y1 (incl. Polio)	D0, D1	D0, D1	D0, D1	D0, D1	12	F	aP
BK142	NL	D0, D28, Y1				D0, D1	13	F	aP
BK143	NL	D0, D28, Y1				D0, D1	12	M	aP
BK037	NL	D0, D28, Y1 (incl. Polio)	D0, D1		D0, D1	D0, D1	14	M	wP
BK039	NL	D0, D28, Y1 (incl. Polio)	D0, D1		D0, D1	D0, D1	15	M	wP
BK040	NL	D0, D28, Y1 (incl. Polio)	D0, D1		D0, D1	D0, D1	14	M	wP
BK042	NL	D0, D28, Y1 (incl. Polio)	D0, D1		D0, D1	D0, D1	15	F	wP
BK043	NL	D0, D28, Y1 (incl. Polio)	D0, D1	D0, D1	D0, D1	D0, D1	14	M	wP
BK044	NL	D0, D28, Y1 (incl. Polio)	D0, D1	D0, D1	D0, D1	D0, D1	14	F	wP
BK141	NL	D0, D28, Y1	D0, D1	D0, D1	D0, D1	D0, D1	15	F	wP
DK037	UK	D0, D28, Y1				D0, D1	13	F	aP
DK038	UK	D0, D28, Y1				D0, D1	13	M	aP
DK039	UK	D0, D28, Y1				D0, D1	12	M	aP
DK040	UK	D0, D28, Y1				D0, D1	12	F	no data
DK041	UK	D0, D28, Y1				D0, D1	11	M	aP
DK042	UK	D0, D28, Y1				D0	13	F	no data
DK043	UK	D0, D28, Y1				D0, D1	11	F	aP
DK044	UK	D0, D28, Y1				D0, D1	13	M	aP
DK046	UK	D0, D28, Y1				D0, D1	13	F	no data
DK047	UK	D0, D28, Y1				D0, D1	13	M	aP
DK048	UK	D0, D28, Y1				D0, D1	12	M	aP
DK146	UK	D0, D28, Y1				D0, D1	11	F	no data

Table S2. Antibody Log-fold change summary statistics and Anova

Antibody	Time	cohort	Mean LogFold change	Stdev	CV (%)	Anova p.value (sex) [1]	Anova p.value (Background)[1]
PT	Day 28	NL	1.06	0.59	55.62	0.64	0.36
FHA	Day 28	NL	1.03	0.61	59.02	0.69	0.37
Prn	Day 28	NL	1.56	0.5	32.14	0.65	0.91
Dt	Day 28	NL	0.81	0.31	38.31	0.51	0.19
TT	Day 28	NL	0.5	0.33	64.59	0.88	0.04
Polio.I	Day 28	NL	0.49	0.37	75.36	0.15	0.01
Polio.II	Day 28	NL	0.73	0.59	79.75	0.46	0.05
Polio.III	Day 28	NL	0.95	0.94	98.88	0.56	0.1
PT	Year 1	NL	0.57	0.46	80.71	0.32	0.46
FHA	Year 1	NL	0.62	0.43	69.81	0.85	0.28
Prn	Year 1	NL	1.08	0.62	57.85	0.42	0.85
Dt	Year 1	NL	0.36	0.25	70.15	0.56	0.04
TT	Year 1	NL	0.24	0.3	124.66	0.82	0.27
PT	Day 28	UK	0.92	0.27	29.49	0.08	NA
FHA	Day 28	UK	1.04	0.45	43.11	0.93	NA
Prn	Day 28	UK	1.12	0.31	28.08	0.53	NA
Dt	Day 28	UK	1.71	0.39	22.88	0.25	NA
TT	Day 28	UK	1.36	0.38	27.76	0.44	NA
PT	Year 1	UK	0.47	0.16	34.58	0.2	NA
FHA	Year 1	UK	0.65	0.36	55.49	0.82	NA
Prn	Year 1	UK	0.76	0.31	40.53	0.74	NA
Dt	Year 1	UK	1.2	0.36	29.91	0.18	NA
TT	Year 1	UK	0.91	0.39	42.95	0.48	NA

[1]Model formula: Antibody log-fold change ~ Gender + Background.
Nominal two-sided p.values are reported.

Table S3. Mass cytometry panel for analysis of circulating immune cells

Antigen	Supplier	Isotype	Clone	Comment	Reference	Lot	Titration (ul)
CD45	Fluidigm France	89Y	HI30	Extra	3089003B	1531706	1
CD8	CHUV/Biolegend	113In	RPA-T8	Extra	301018	24082017	1
CD4	CHUV/Biolegend	115In	RPA-T4	Extra	300516	07072017	1
CD19	Fluidigm France	142Nd	HIB19	Extra	3142001B	1031708	2
HLA-DR	Fluidigm France	143Nd	L243	Extra	3143013B	3421602	2
CD69	Fluidigm France	144Nd	FN50	Extra	3144018B	0041711	1
CD31	Fluidigm France	145Nd	WM59	Extra	3145004B	3531201	2
CD86	CHUV/Biolegend	146Nd	IT2.2	Extra	305435	20022015	1
CD7	Fluidigm France	147Sm	CD7-6B7	Extra	3147006B	2741608	1
CD16	Fluidigm France	148Nd	3G8	Extra	3148004B	0831702	2
IL12p40	CHUV/Biolegend	149Sm	C11.5	Intra	501813	10072017	0.8
IFNa	CHUV/Myltenyi Biotec	150Nd	LT27.295	Intra	130-092-604	10072017	0.6
CD123	Fluidigm France	151Eu	6H6	Extra	3151001B	0521709	2
TNFa	Fluidigm France	152Sm	Mab11	Intra	3152002B	0841602	0.5
IL1b	CHUV/Lab Force	153Eu	AS10	Intra	LS-C26495-500	29052017	0.5
IL6	Fluidigm France	154Sm	MQ2-13AS	Intra	3154011B	1121511	0.8
CD27	Fluidigm France	155Gd	L128	Extra	3155001B	1031712	1
TCRg/d	CHUV/Biolegend	156Gd	B1	Extra	331204	17072017	1
CD33	Fluidigm France	158Gd	WM53	Extra	3158001B	0571502	1
NKp30	Fluidigm France	159Tb	Z25	Extra	3159017B	1481505	1
CD14	Fluidigm France	160Gd	M5E2	Extra	3160001B	1351724	2
CD1c	CHUV/Biolegend	161Dy	L161	Extra	331502	06072017	1
CD11c	Fluidigm France	162Dy	Bu15	Extra	3162005B	1561503	2
CD62L	CHUV/Biolegend	163Dy	DREG-56	Extra	304835	17072017	1
IL17a	Fluidigm France	164Dy	N49-653	Intra	3164002B	0161713	0.8
IFNg	Fluidigm France	165Ho	B27	Intra	3165002B	0911301	0.5
NKG2D	Fluidigm France	166Er	ON72	Extra	3169013B	0091703	1
CD38	Fluidigm France	167Er	HIT2	Extra	3167001B	0191508	1
CD66b	CHUV/BD Biosciences	168Er	G10F5	Extra	555723	29032017	1
NKG2A	Fluidigm France	169Tm	Z199	Extra	3169013B	0961515	1
CD3	Fluidigm France	170Er	UCHT1	Extra	3170001B	2581608	1
Granzyme B	Fluidigm France	171Yb	GB11	Intra	3171002B	1031504	0.5
CD15	Fluidigm France	172Yb	W6D3	Extra	3172021B	0791506	1
CD141	Fluidigm France	173Yb	1A4	Extra	3173002B	2351601	2
NKG2	Fluidigm France	174Yb	HP-3D9	Extra	3174015B	2181508	1
Perforin	Fluidigm France	175Lu	B-D48	Intra	3175004B	3421604	0.6
CD56	Fluidigm France	176Yb	R19-760	Extra	3176013B	1031703	2
CD11b	Fluidigm France	209Bi	ICRF44	Extra	3209003B	2241612	1

Table S4. Mass cytometry differential cytokine and abundance analysis results[1]

subpopulation	variable	contrast	Estimate	df	p.value	lower.CI	upper.CI	p.adj	threshold
Classical Monocytes	IL12p40	D1 - D0	0.5387	11	1E-05	0.39488	0.68245	2E-05	***
Classical Monocytes	IL6	D1 - D0	1.6658	11	2E-04	1.06109	2.27049	0.0003	***
Classical Monocytes	IFNa	D1 - D0	1.5258	11	3E-04	0.93373	2.11781	0.0005	***
Classical DC2	IFNa	D1 - D0	1.2548	11	6E-04	0.71902	1.79065	0.0011	**
Classical DC2	IL6	D1 - D0	0.9246	11	8E-04	0.51339	1.33589	0.0014	**
Plasmacytoid DCs	IFNa	D1 - D0	1.707	11	0.002	0.86182	2.55217	0.0031	**
Classical Monocytes	IFNg	D1 - D0	0.1757	11	0.002	0.08689	0.26441	0.0034	**
Plasmacytoid DCs	IFNg	D1 - D0	0.3549	11	0.003	0.16284	0.54703	0.0054	**
Intermediate Monocytes	IL12p40	D1 - D0	0.5477	11	0.006	0.21895	0.87653	0.0101	*
Intermediate Monocytes	IFNa	D1 - D0	1.8419	11	0.012	0.60059	3.08314	0.0192	*
Classical Monocytes	TNFa	D1 - D0	0.2443	11	0.022	0.05717	0.43147	0.0361	*
Classical DC2	TNFa	D1 - D0	0.5209	11	0.025	0.11342	0.92845	0.0392	*
Classical DC1	ILb	D1 - D0	2.8181	11	0.037	0.39856	5.23768	0.0582	ns
Non-classical Monocytes	IL12p40	D1 - D0	0.3753	11	0.047	0.03323	0.71738	0.0729	ns
Plasmacytoid DCs	ILb	D1 - D0	0.8355	11	0.053	0.05161	1.61939	0.0793	ns
Classical DC2	ILb	D1 - D0	1.1674	11	0.053	0.07098	2.2639	0.0793	ns
Non-classical Monocytes	IFNa	D1 - D0	0.7292	11	0.058	0.02652	1.43179	0.0859	ns
Classical DC1	IFNg	D1 - D0	1.3675	11	0.064	-0.0014	2.73644	0.0927	ns
surface-	IFNg	D1 - D0	0.2938	11	0.084	-0.0208	0.6085	0.1198	ns
Classical DC2	IL12p40	D1 - D0	0.337	11	0.09	-0.032	0.70606	0.1267	ns
Plasmacytoid DCs	IL12p40	D1 - D0	0.3202	11	0.101	-0.0444	0.68468	0.1407	ns
Non-classical Monocytes	IFNg	D1 - D0	0.2831	11	0.12	-0.0594	0.62566	0.1639	ns
Intermediate Monocytes	ILb	D1 - D0	-1.1445	11	0.121	-2.5329	0.24377	0.1639	ns
Plasmacytoid DCs	IL6	D1 - D0	0.3084	11	0.144	-0.0914	0.7082	0.1925	ns
Classical DC1	IL6	D1 - D0	1.07	11	0.198	-0.5205	2.66062	0.2602	ns
Classical Monocytes	ILb	D1 - D0	-1.7759	11	0.24	-4.6909	1.13909	0.3118	ns
surface-	TNFa	D1 - D0	-0.2514	11	0.265	-0.6872	0.18453	0.3391	ns
Non-classical Monocytes	ILb	D1 - D0	-0.4968	11	0.299	-1.4258	0.43215	0.3761	ns
surface-	IL12p40	D1 - D0	-0.3133	11	0.302	-0.9023	0.27562	0.3761	ns
Classical DC1	IL12p40	D1 - D0	-0.4743	11	0.356	-1.4765	0.52786	0.4377	ns
Immature APCs	ILb	D1 - D0	0.649	11	0.361	-0.739	2.03708	0.439	ns
surface-	IL6	D1 - D0	-0.4913	11	0.436	-1.7248	0.74217	0.5226	ns
surface-	ILb	D1 - D0	0.2473	11	0.448	-0.3926	0.88707	0.5307	ns
Immature APCs	IFNg	D1 - D0	0.3671	11	0.521	-0.7595	1.49371	0.6094	ns
Immature APCs	TNFa	D1 - D0	0.4624	11	0.538	-0.976	1.90086	0.6219	ns
Intermediate Monocytes	IFNg	D1 - D0	0.1094	11	0.599	-0.3018	0.52058	0.6842	ns
Non-classical Monocytes	TNFa	D1 - D0	-0.2489	11	0.658	-1.363	0.86517	0.743	ns
Immature APCs	IFNa	D1 - D0	0.6489	11	0.673	-2.3972	3.69495	0.7509	ns
Immature APCs	IL6	D1 - D0	0.1531	11	0.844	-1.3924	1.69859	0.9221	ns
Classical DC1	TNFa	D1 - D0	0.1214	11	0.845	-1.117	1.35988	0.9221	ns
Immature APCs	IL12p40	D1 - D0	0.1195	11	0.857	-1.1977	1.43677	0.9241	ns
Intermediate Monocytes	TNFa	D1 - D0	-0.1445	11	0.887	-2.1679	1.87893	0.9461	ns
Plasmacytoid DCs	TNFa	D1 - D0	0.0263	11	0.899	-0.3858	0.43848	0.9482	ns
Classical DC1	IFNa	D1 - D0	0.1196	11	0.92	-2.245	2.48418	0.9554	ns
Non-classical Monocytes	IL6	D1 - D0	-0.0329	11	0.926	-0.7326	0.66693	0.9554	ns
surface-	IFNa	D1 - D0	0.06	11	0.937	-1.4458	1.5658	0.9567	ns
Classical DC2	IFNg	D1 - D0	-0.007	11	0.964	-0.3179	0.30379	0.9741	ns
Intermediate Monocytes	IL6	D1 - D0	-0.017	11	0.982	-1.5379	1.5039	0.9823	ns
Immature APCs	abundance	D1 - D0	-0.0005	11	0.008	-0.0009	-0.0002	0.0133	*
Classical Monocytes	abundance	D1 - D0	0.1082	11	0.008	0.03951	0.17689	0.0133	*
Classical DC2	abundance	D1 - D0	-0.0017	11	0.034	-0.0031	-0.0003	0.0489	*
Classical DC1	abundance	D1 - D0	-0.0002	11	0.053	-0.0004	-1E-05	0.0712	ns
Plasmacytoid DCs	abundance	D1 - D0	-0.001	11	0.139	-0.0023	0.00028	0.1711	ns
Non-classical Monocytes	abundance	D1 - D0	0.0019	11	0.185	-0.0008	0.00457	0.2113	ns
surface-	abundance	D1 - D0	-0.0005	11	0.465	-0.0017	0.00077	0.4711	ns
Intermediate Monocytes	abundance	D1 - D0	0.0006	11	0.471	-0.001	0.00225	0.4711	ns

[1]Statistical significance (false-discovery rate (FDR) adjusted two-sided p.value) was calculated from a mixed-effects regression model for each response (abundance or cytokine) with study day and participant number specified as fixed and random effects.

Table S5. Mass cytometry panel phosphosignaling

Antigen	Supplier	Isotype	Clone	Comment	Reference	Lot	Titration (ul)
CD8	CHUV/Biolegend	113In	RPA-T8	Extra	301018	08112018	0.3
CD4	CHUV/Biolegend	115In	RPA-T4	Extra	300516	11092017	0.8
CD45	Fluidigm France	141Pr	HI30	Extra	3141009B	3331705	0.7
CD19	Fluidigm France	142Nd	HIB19	Extra	3142001B	0381907	1.5
HLA-DR	Fluidigm France	143Nd	L243	Extra	3143013B	0941808	0.75
CD69	Fluidigm France	144Nd	FN50	Extra	3144018B	3041705	0.63
CD31	Fluidigm France	145Nd	WM59	Extra	3145004B	1351719	0.6
CD86	CHUV/Biolegend	146Nd	IT2.2	Extra	305410	17072017	0.4
CD7	Fluidigm France	147Sm	CD7-6B7	Extra	3147006B	2741608	0.56
CD16	Fluidigm France	148Nd	3G8	Extra	3148004B	1571806	0.75
CD56	Fluidigm France	149Sm	NCAM16.2	Extra	3149021B	3331703	0.3
pSTAT5	Fluidigm France	150Nd	47	Intra	3150005A	0391801	1
CD123	Fluidigm France	151Eu	6H6	Extra	3151001B	2431808	0.8
pAKT	Fluidigm France	152Sm	D9E	Intra	3152005A	1201812	2
pSTAT1	Fluidigm France	153Eu	58D6	Intra	3153003A	2001805	2
CD3	Fluidigm France	154Sm	UCHT1	Extra	3154003B	3391723	0.25
CD27	Fluidigm France	155Gd	L128	Extra	3155001B	1731805	0.5
pp38	CHUV/Biolegend	156Gd	D3F9	Intra	3156002A	3441603	1
pSTAT3	Fluidigm France	158Gd	4/P-STAT3	Intra	3158005A	1501710	2.6
pMAPKAPK2	Fluidigm France	159Tb	27B7	Intra	3159010A	1111705	2
CD14	Fluidigm France	160Gd	M5E2	Extra	3160001B	0241814	1.2
CD1c	CHUV/Biolegend	161Dy	L161	Extra	331502	17072017	0.6
CD11c	Fluidigm France	162Dy	Bu15	Extra	3162005B	0041909	1.2
CD62L	CHUV/Biolegend	163Dy	DREG-56	Extra	304835	17072017	0.6
CD45RA	Fluidigm France	164Dy	HI100	Extra	555486	16082018	0.5
pNFKb	Fluidigm France	165Ho	K10-895.12.50	Intra	558393	08112018	1.4
NKG2D	Fluidigm France	166Er	ON72	Extra	3169013B	2511702	0.63
CD38	Fluidigm France	167Er	HIT2	Extra	3167001B	1031705	0.38
CD66b	CHUV/BD Biosciences	168Er	G10F5	Extra	555723	02072018	0.3
NKG2A	Fluidigm France	169Tm	Z199	Extra	3169013B	0961515	0.63
pERK 1/2	Fluidigm France	170Er	MILAN8R	Intra	14-9109-82	00072019	1
CD20	Fluidigm France	171Yb	2H7	Extra	3171012B	0251702	0.5
CX3CR1	Fluidigm France	172Yb	2A9-1	Extra	3172017B	1351727	0.5
CD141	Fluidigm France	173Yb	1A4	Extra	331204	2351601	0.5
TCRgd	CHUV/Biolegend	173Yb	B1	Extra	3173002B	05092018	0.75
pSTAT4	Fluidigm France	174Yb	38/p-Stat4	Intra	3174005A	3271607	2
pSTAT6	Fluidigm France	175Lu	18	Intra	3175009A	0641501	2
pCREB	Fluidigm France	176Yb	87G3	Intra	3176005A	2371710	1
CD11b	Fluidigm France	209Bi	ICRF44	Extra	3209003B	2241612	0.8
CD45	CHUV/Biolegend	194Pt	HI30	Extra	304002	07122018	0.5
CD45	CHUV/Biolegend	195Pt	HI31	Extra	304002	11092018	0.5
CD45	CHUV/Biolegend	198Pt	HI30	Extra	304002	11042019	0.5
CD45	Fluidigm France	89Y	HI30	Extra	3089003B	3321821	0.6

Table S6. Flow cytometry panel for single-cell sorting of innate immune cells

Antigen	Supplier	Fluorochrome	Clone	Catelog No.	Lot	Titration (ul)	Comment
CD141	BD Biosciences	BV421	1A4	565321	6291827	2.5	Phenotypic marker
CD45	Cytognos	OC515	GA90	CYT-45OC	520371	10	Phenotypic marker
CD62L	Biolegend	BV605	DREG-56	304834	B213455	5	Phenotypic marker
HLA-DR	BD Biosciences	BV711	G46-6	563696	7068615	2.5	Phenotypic marker
CD16	BD Biosciences	BV786	3G8	563690	7139586	5	Phenotypic marker
CD1c	BD Biosciences	BB515	F10/21A3	565054	6083777	5	Phenotypic marker
CD36	Immunostep	PerCP Cy5.5	CLB-IVC7	36PP5-100T	590015	10	Phenotypic marker
Slan	Miltenyi Biotech	PE	DD.1	130-093-029	5161227022	10	Combined with FCeR1, phenotypic marker
FCeR1	eBioscience	PE	AER-37	12-5899-42	4273945	5	Combined with Slan, phenotypic marker
CD14	BD Biosciences	PE-CF594	MOP9	562335	7174983	5	Phenotypic marker
CD33	BD Biosciences	PE-Cy7	P67.6	333952	6279949	5	Phenotypic marker
IREM2	Immunostep	APC	UP-H2	IREM2A-100T	123541	10	Combined with CD303, phenotypic marker
CD303	Miltenyi Biotech	APC	AC144	130-090-905	5161221268	10	Combined with IREM2, phenotypic marker
CD56	Biolegend	APC-Cy7	HCD56	318332	B233132	5	Phenotypic marker
CD3	BD Biosciences	BUV395	SK7	564001	6294654	5	Combined with CD19, exlcusion marker
CD19	BD Biosciences	BUV395	SJ25C1	563549	6273604	5	Combined with CD3, exlcusion marker
FVDBlue	Molecular Probes	NA	NA	L34962		10	Viability marker

Immunological effects of an acellular pertussis booster vaccination in children, young adults and elderly with different immunisation background.

An international study in Finland, the Netherlands and the United Kingdom

Booster against pertussis (Bert) study

PERISCOPE

The resurgence of pertussis, as well as the persistent low level of vaccination coverage and high infant mortality caused by pertussis in low income countries, urged for a concerted effort to improve current prophylactic vaccines and vaccination strategies for pertussis. This concerted effort resulted in the establishment of a European consortium, called **PERISCOPE** (PERtussIS COrrelates of Protection Europe). PERISCOPE is a unique public-private partnership between a large group of pertussis experts from 15 universities across Europe, 3 national public health institutes, 2 small enterprises and two partners from the European federation of pharmaceutical industries and associations (EFPIA), Sanofi Pasteur and GlaxoSmithKline. The PERISCOPE consortium aims to generate knowledge on immune responses to pertussis. Better understanding of human biomarkers of protective immune responses to *Bordetella pertussis*, and its waning immunity is needed to accelerate the design and testing of new pertussis vaccines with a longer duration of protection. In the figure below, we present an overview of the interactions between the 7 work packages (WPs) in PERISCOPE and the associated tasks. The current protocol is part of WP 3, which also contains a European maternal-infant vaccination study in the Gambia and in Europe (NL, UK, Finland). Through a number of carefully harmonised preclinical (WP1) and clinical (WP2&3) studies, we will be able to systematically compare immune responses to existing pertussis vaccines (acellular pertussis vaccine versus whole cell pertussis vaccine) as well as infection induced immunity (WP2).These studies will provide samples and clinical parameters for analysis in WP5, in the biomarker discovery platform. In order to facilitate a streamlined regulatory approval process for these biomarkers we will engage regulatory authorities from the onset of the project (WP4).To ensure adequate knowledge transfer, training of consortium partners, and optimal visibility of the consortium activities to external stakeholders, including the general public, we have designed a clear communication strategy (WP7). Finally, all activities will be carefully managed so that the partners will successfully deliver on the tasks and milestones set out in the project (WP6).

Overview of the interrelated work packages (01 to 07) and their studies that constitute the PERISCOPE consortium

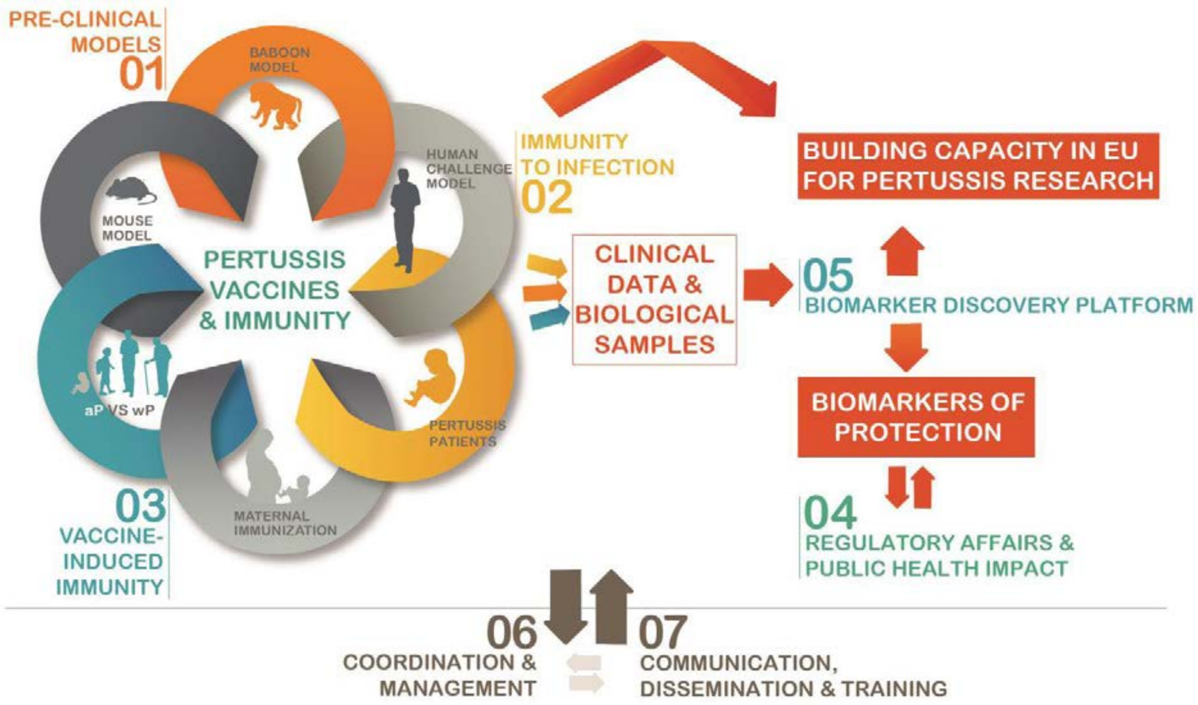

Title: Immunological effects of an acellular pertussis booster vaccination in children, young adults and elderly. An international study in Finland, the Netherlands and the United Kingdom

Protocol ID	NL60807.100.17/ R17.039/ IIV-316
EudraCT	2016-003678-42
Short title	Booster against pertussis (Bert) study
Version	3.0
Date	14-08-2017
Coordinating investigator / project leader	xxx (Centre for infectious disease control, RIVM)
Principal investigator for the Netherlands (in Dutch: hoofdonderzoeker / uitvoerder)<br><br>Principal investigator for UK<br><br>Principal investigator for Finland	xxx (Spaarne Hospital, Hoofddorp, the Netherlands)<br><br>xxx (Oxford Vaccine Group, Oxford, United Kingdom)<br><br>xxx (Turku University Hospital, Turku, Finland)
Research site the Netherlands<br><br>Research site UK<br><br>Research site Finland	National Institute for Public Health and the Environment – RIVM, Centre for Infectious Disease Control, Antonie van Leeuwenhoeklaan 9, 3721 MA, Bilthoven, The Netherlands<br><br>Centre for Clinical Vaccinology & Tropical Medicine (CCVTM), Churchill Hospital, Oxford OX3 7LE, United Kingdom<br><br>Department of Medical Microbiology and Immunology, Turku University and Department of Paediatrics and Adolescent Medicine, Turku University Hospital Kiinamyllynkatu 13, 20520 Turku, Finland

Sponsor the Netherlands (in Dutch: verrichters/ opdrachtgevers)	Rijksinstituut voor Volksgezondheid en Milieu National Institute for Public Health and the Environment – RIVM, Antonie van Leeuwenhoeklaan 9, 3721 MA, Bilthoven, The Netherlands
Sponsor UK	Clinical Trials and Research Governance Joint Research Office, Block 60, Churchill Hospital, Old Road, Headington, Oxford OX3 7LE, United Kingdom
Sponsor Finland	Turun Yliopisto (Turku University) (UTU), 20500 Turku, Finland
Independent physician the Netherlands (NL only)	xxx
Laboratory sites in the Netherlands	1. RIVM; Centre for Infectious Diseases (Cib); Centre for Immunology of Infectious diseases and Vaccines (IIV)<br>2. Leiden University Medical Center (LUMC); Department of Immunohematology and Blood Transfusion (IHB)<br>3. Radboud University Medical Center (RUMC); Laboratory of Pediatric Infectious Diseases, Department of Pediatrics, Radboud Institute for Molecular Life Sciences
Laboratory site in the UK	Centre for Clinical Vaccinology & Tropical Medicine (CCVTM), Churchill Hospital, Oxford OX3 7LE, United Kingdom
Laboratory site in Finland	Department of Medical Microbiology and Immunology, Turku University, Kiinamyllynkatu 13, 20520 Turku, Finland

Monitor for the Netherlands	Rijksinstituut voor Volksgezondheid en Milieu<br>National Institute for Public Health and the Environment (RIVM)
Monitor for the UK	Clinical Trials and Research Governance (CTRG)
Monitor for Finland	SMERUD Medical Research

PROTOCOL SIGNATURE SHEET Netherlands

Name	Signature	Date
**Sponsor or legal representative:**<br>**National institute for public health and the environment (RIVM), centre of Immunology of Infectious Diseases and Vaccines, department head**<br>**xxx**		
**Coordinating investigator/project leader:**<br>**xxx**		
**Principal investigator:**<br>**xxx**		
**Study coordinator on behalf of sponsor: xxx**		

TABLE OF CONTENTS

PERISCOPE 2
PROTOCOL SIGNATURE SHEET 7
TABLE OF CONTENTS 8
LIST OF ABBREVIATIONS AND RELEVANT DEFINITIONS 11
SUMMARY 13
1. INTRODUCTION AND RATIONALE 19
2. OBJECTIVES 24
  2.1 Primary objective 24
  2.2 Secondary objectives 24
  2.3 Exploratory objectives 24
3. STUDY DESIGN 26
  3.1 Vaccination and collection of blood samples 26
  3.2 Study sites 29
4. STUDY POPULATION 30
  4.1 Population 30
  4.2 Inclusion criteria 30
  4.3 Exclusion criteria 30
  4.4 Delay criteria 31
  4.5 Sample size calculation 32
5. TREATMENT OF SUBJECTS 33
  5.1 Investigational product/treatment 33
  5.2 Use of co-intervention 33
  5.3 Escape medication 33
6. INVESTIGATIONAL PRODUCT 34
  6.1 Name and description of investigational product 34
  6.2 Summary of findings from non-clinical studies 34
  6.3 Summary of findings from clinical studies 34
  6.4 Summary of known and potential risks and benefits 34
  6.5 Description and justification of route of administration and dosage 34
  6.6 Dosages, dosage modifications and method of administration 35
  6.7 Preparation and labelling of Investigational Medicinal Product 35
  6.8 Drug accountability 35
7. NON-INVESTIGATIONAL PRODUCT 36
8. METHODS 37

8.1 Study endpoints 37
8.1.1 Main study endpoint 37
8.1.1.1 Pertussis toxin specific IgG antibody concentration and avidity in serum 37
8.1.2 Secondary study endpoints 37
8.1.2.1 Specific IgG levels in serum..37
8.1.2.2 Other serological endpoints 37
8.1.2.3 Cellular endpoints..38
8.1.2.4 Logistic endpoints..38
8.1.3 Exploratory study endpoints 38
8.2 Randomisation, blinding and treatment allocation 39
8.3 Study procedures...39
8.3.1 Detailed description of study visits..39
8.3.1.1 Visit T0 39
8.3.1.2 *Visit T1* 40
8.3.1.3 *Visit T2* 40
8.3.1.4 *Visit T3* 40
8.3.1.5 *Visit T4* 40
8.3.1.6 *Visit T5* 41
8.3.2 Procedures for sample collection, labelling, processing and storage 41
8.3.3 Laboratory tests (primary objective) 42
8.3.4 Other laboratory tests..42
8.4 Withdrawal of individual subjects..44
8.5 Replacement of individual subjects after withdrawal.................................45
8.6 Follow-up of subjects withdrawn from treatment..45
8.7 Premature termination of the study..45
9. SAFETY REPORTING 46
9.1 Premature termination or suspension of a trial 46
9.2 Adverse events, serious adverse events and suspected unexpected serious adverse reactions 46
9.2.1 Adverse events 46
9.2.2 Serious adverse events...46
9.2.3 Suspected unexpected serious adverse reactions.......................................48
9.3 Annual safety report 49
9.4 Follow-up of adverse events...49
9.5 Data safety monitoring committee 49
10. STATISTICAL ANALYSIS...50
10.1 Primary study parameter(s) 50

10.1.1 Serum specific pertussis toxin IgG antibodies 50
10.2 Interim analysis 51
10.3 Comparative analyses....................51
10.4 Data management....................51
11. ETHICAL CONSIDERATIONS....................53
11.1 Regulation statement 53
11.2 Recruitment and consent....................53
11.3 Objection by minors or incapacitated subjects....................56
11.4 Benefits and risks assessment, group relatedness....................56
11.5 Compensation for injury 56
11.6 Incentives....................57
12. ADMINISTRATIVE ASPECTS, MONITORING AND PUBLICATION 58
12.1 Handling and storage of data and documents 58
12.2 Handling and storage of samples 58
12.3 Monitoring and Quality Assurance....................59
12.4 Amendments....................59
12.5 Annual progress report....................60
12.6 End of study report....................60
12.7 Public disclosure and publication policy....................60
13. STRUCTURED RISK ANALYSIS....................61
13.1 Potential issues of concern....................61
13.2 Synthesis 61
14. REFERENCES 62
APPENDIX A: Laboratories involved in analysis of samples from trial**Error! Bookmark not defined.**
APPENDIX B: List of institutions and abbreviations.................... **Error! Bookmark not defined.**
APPENDIX C: Sample plan.................... **Error! Bookmark not defined.**

LIST OF ABBREVIATIONS AND RELEVANT DEFINITIONS

**AE**	Adverse event
**aP**	Acellular pertussis
**AR**	Adverse reaction
**BAI**	Bacterial adherence inhibition assay
**Bert**	Booster against pertussis
**Bp**	*Bordetella pertussis*
**CHCD**	Child health computer department
**CI**	Confidence interval
**CPMP**	Committee for proprietary medical products
**CTRG**	Clinical trials and research governance
**CyTOF**	Cytometry by time of flight
**DSMC**	Data safety monitoring committee
**ELISpot**	Enzyme-linked immunospot assay
**EU**	European Union
**FHA**	Filamentous hemagglutinin
**FI**	Finland
**Fim**	Fimbriae
**GCP**	Good clinical practice
**GP**	General practitioner
**GSK**	GlaxoSmithKline
**ICF**	Informed consent form
**ICH**	International committee on harmonisation
**ITT**	Intention-to-treat
**IU**	International units
**MLF**	Mucosal lining fluid
**MREC**	Medical research ethics committee, in Dutch: medisch ethische toetsing commissie (METC)
**MIA**	Multiplex immunoassay
**NIP**	National immunisation programme
**NL**	The Netherlands
**OPA**	Opsonophagocytosis assay
**PBMC**	Peripheral blood mononuclear cells
**PI**	Principal investigator
**PIF**	Participants information
**Prn**	Pertactin

**PT**	Pertussis toxin
**PTNA**	Pertussis toxin neutralisation assay
**RNA-seq**	RNA-sequencing
**SAE**	Serious adverse event
**SAM**	Synthetic absorptive matrices
**SAR**	Serious adverse reaction
**SBA**	Serum bactericidal activity assay
**scRNA**	Single cell RNA
**SOP**	Standard operating procedure
**SmPC**	Summary of product characteristics (in Dutch: officiële productinformatie IB1-tekst)
**Sponsor**	The sponsor is the party that commissions the organisation or performance of the research, for example a pharmaceutical company, academic hospital, scientific organisation or investigator. A party that provides funding for a study but does not commission it, is not regarded as the sponsor, but referred to as a subsidising party
**SUSAR**	Suspected unexpected serious adverse reaction
**UK**	United Kingdom
**WHO**	World Health Organisation
**WMA**	World Medical Association
**WMO**	Medical research involving human subjects act (in Dutch: wet medisch-wetenschappelijk onderzoek met mensen
**wP**	Whole-cell pertussis

Abbreviations for vaccines in the study protocol

**dT**	Reduced diphtheria toxoid and tetanus toxoid vaccine
**DTaP**	Diphtheria and tetanus toxoid, acellular pertussis vaccine
**DTap**	Diphtheria and tetanus toxoid, reduced acellular pertussis vaccine
**dTaP**	Reduced diphtheria toxoid, tetanus toxoid and acellular pertussis vaccine
**dTap**	Reduced diphtheria toxoid and tetanus toxoid, reduced acellular pertussis vaccine
**DTwP**	Diphtheria and tetanus toxoid, whole-cell pertussis vaccine
**IPV**	Inactivated poliovirus vaccine
**OPV**	Orally administered, live attenuated poliovirus vaccine
**MMR**	Mumps, measles and rubella vaccine
**Hib**	Haemophilus influenzae Type B vaccine
**HepB**	Hepatitis B vaccine

SUMMARY

Rationale: pertussis, or whooping cough, is caused by the bacterium *Bordetella pertussis* (*B. pertussis*) and is an acute and serious respiratory infection, in particular for young and unvaccinated children. However, despite high vaccination coverage (95%), pertussis is re-emerging in the Netherlands since 1996. This phenomenon is also observed in most other western countries. The most recent epidemic in 2012 in the United Kingdom (UK) and the Netherlands highlighted the vulnerability of infants for a pertussis infection, causing 15 deaths all together. The pertussis infection rate in adolescents and adults has increased as well in the past years. This elevated incidence in adults is a risk factor for young babies, since infants are most often infected with *B. pertussis* through their mother.

The main purpose of this study is to investigate the dynamics and longitudinal effects of an acellular pertussis (aP) booster vaccination in children, young adults and elderly, on long-term humoral and cellular memory immunity against *B. pertussis*. The study will be performed in three European countries (UK, Finland and the Netherlands) with a different epidemiological background for pertussis incidence. In addition, different age groups had different primary schedules with whole cell pertussis (wP) or aP vaccines in their first year of life. Long-term memory responses will be analysed following aP booster vaccination to provide a detailed understanding of immunity to *B. pertussis* and to assess novel biomarkers as potential surrogates of long-lived protective immunity. This will include a detailed assessment of antigen-specific B and T cell responses and serology assays for pertussis antigens. In addition, the effect of booster vaccination on dynamic changes in immune cell subsets and gene transcription will be investigated.

Objective: to investigate the effects of aP booster vaccination in children, young adults and elderly on the (long-term) immune response to *B. pertussis* in three European countries with a different epidemiological background and primary vaccination schedule for pertussis.

Study design: longitudinal intervention study

Study population: this study includes only healthy participants. Eligible to participate in this study are children born between 2007 and 2010 (7 to 10 years: cohort A), children born between 2002 and 2006 (11 to 15 years: cohort B), young adults born between 1983 and 1997 (20 to 34 years of age: cohort C) and elderly born between 1947 and 1957 (60 to 70 years of age: cohort D).

In the Netherlands, participants in cohort A received exclusively a diphtheria toxoid, tetanus toxoid, acellular pertussis vaccine combined with inactivated poliovirus vaccine (DTaP-IPV). Cohort B is a mixture of participants exclusively primed with DTaP-IPV and participants exclusively primed with a whole-cell pertussis combination vaccine (DTwP-IPV). Participants in the Netherlands in cohorts C and D generally received exclusively DTwP-IPV. Some participants of cohort D might not have been vaccinated at all. Vaccinations were administered at 2, 3, 4 and 11 months of age (cohorts A and B), or at 3, 4, 5, and 11 months of age (cohorts C and D) together with all other regular vaccinations according to the Dutch national immunisation programme (NIP). Cohort B allows the opportunity to compare the effects of an aP booster vaccination in children who were primed with wP versus primed with aP in their first year of life (Figure 1).

In the UK, similar to the Netherlands, the participants from cohorts C and D have only received DTwP, with IPV (cohort D) or orally administered, live attenuated poliovirus vaccine (OPV) (cohort C), and some participants from cohort D might not have received a pertussis vaccine as part of their primary immunisations. Participants from cohort D would receive the vaccine according to the UK extended schedule at 3, 4.5-5 and 8.5-11 months of age, and participants from cohort C would have a mixture of the extended schedule and the accelerated schedule (2,3 and 4 months of age). Cohort B would have a mixture of completely wP or completely aP priming. Participants from cohort A would have a complete aP priming schedule. The priming vaccination schedules for both of the cohorts A and B in the UK, was the accelerated schedule at 2, 3 and 4 months of age (Figure 1).

In Finland all participants from cohort A have received DTaP-IPV-Haemophilus influenzae Type B vaccine (Hib) at 3, 5, 12 months and a booster of diphtheria and tetanus toxoid, reduced acellular pertussis vaccine (DTap)-IPV at 4 years of age. Children born 2007-8 (age 9-10 years) had received a 2-component aP vaccine (pertussis toxin (PT) and filamentous hemagglutinin (FHA), Pentavac) as primary immunisation and another 2-component (Tetravac) booster at 4 years of age, whereas children born 2009 and afterwards had received a 3-component aP vaccine (PT, FHA and pertactin (Prn)) (Infanrix®-IPV-Hib) as primary immunisation. Participants in cohort B received either DTwP at 3, 4, 5 and 24 months of age and a booster of dTap (Boostrix®) at 6 years of age (those of 13-15 years of age), or DTaP-IPV-Hib at 3, 5 and 12 months and a booster of DTaP-IPV at 4 years of age (those of 11-12 years of age). Adults from cohort C have received DTwP at 3, 4, 5 and 24 months of age. From cohort D, those who are born before 1952 are unvaccinated whereas those born 1952 and afterwards have very likely received a wP.

Age	FI	UK	NL
2 months		DTaP-IPV	DTaP-IPV
3 months	DTaP-IPV	DTaP-IPV	DTaP-IPV
4 months		DTaP-IPV	DTaP-IPV
5 months	DTaP-IPV		
11 months			DTaP-IPV
12 months	DTaP-IPV		
3 years		DTaP-IPV	
4 years	DTaP-IPV		DTaP-IPV
9 years			dT-IPV
14 years	dTap	dT-IPV	
15 years			
adults	dTap (military)	dTap (maternal vaccination)	

Figure 1: comparison of the regular DTaP-IPV vaccination schedule in the three countries

Intervention: participants will receive one injection of reduced diphtheria toxoid, tetanus toxoid and reduced acellular pertussis vaccine (dTap)-IPV, (Boostrix®-IPV, GlaxoSmithKline (GSK)) combination vaccine intramuscularly in the upper arm. MLF samples will be taken before (T0) and 28 days (T4) and 1 year (T5) year after vaccination Collection of MLF samples will be performed only in the Netherlands. Venous blood samples will be drawn before (T0) and at 1 (T1), 7 (T2), 14 (T3) and 28 (T4) days post-intervention and again 1 year (T5) after vaccination. Adults (cohorts C and D) will donate blood samples at T0, T2, T3, T4 and T5. Children will donate only at selected time points. All children in cohorts A and B will donate blood samples at T0, T4 and T5. Additionally, the children will be further divided in subcohorts for additional blood draws at ***one*** of the following time points: cohorts A at T2 or T3, cohort B at T1, T2 or T3. Summarising, children will be asked to donate blood 4 times, and young adults and elderly will be asked to donate blood 5 times in total over the entire study duration of 12 months (Table 1).

Main study endpoints: PT specific IgG antibodies at day 28 (T4). Comparisons of pertussis vaccine antigen-specific antibody, as well as B and T cell responses before, and at several

time points after vaccination are secondary endpoints. Table 2 shows an overview of all study objectives and endpoints.

Table 1: study schedule

Visit number	Actions Adults	Actions Children
T0: first visit	- Signing of informed consent<br>- Draw first blood sample<br>- Collect first mucosal lining fluid (MLF) sample (NL only)<br>- dTap vaccination	- Signing of informed consent by parents and children<br>- Draw first blood sample<br>- Collect first MLF sample (NL only)<br>- dTap vaccination
T1: visit 1 day after T0 (± 4 hours)	-	- Draw second blood sample for subcohort B1<br>-
T2:visit 7 days after T0 (± 1 day)	- Draw second blood sample	- Draw second blood sample for subcohort B2 and A1<br>-
T3: visit 14 days after T0 (± 2 days)	- Draw third blood sample	- Draw second blood sample for subcohort B3 and A2
T4: visit 28 days after T0 (± 4 days)	- Draw fourth blood sample<br>- Collect second MLF sample (NL only)	- Draw third blood sample<br>- Collect second MLF sample (NL only)
T5: visit 1 year after T0 (± 4 weeks)	- Draw fifth and final blood sample<br>- Collect third MLF sample (NL only)	- Draw fourth and final blood sample<br>- Collect third MLF sample (NL only)<br>- Mumps, measles and rubella vaccine (MMR) vaccination according NIP for cohort A (NL only)

Nature and extent of the burden and risks associated with participation, benefit and group relatedness: participants will benefit from participating in this study by receiving an additional Boostrix®-IPV vaccination. From the worldwide public health perspective, participation in this study will contribute to insight in pertussis immunity. Although vaccination and especially venepunctures may be unpleasant, they are considered low risk invasive procedures. These risks will be mitigated by the performance of all procedures by experienced personnel. The sampling frequency is intensive for the young adults and elderly. To minimise sampling frequency for children, cohorts A and B will be divided into one of the subcohorts (i.e. the three 'fixed' time points plus one additional time point). This will also minimise the total blood volume drawn from children in the first two weeks post-intervention.

Boostrix®-IPV is a registered vaccine. Adverse reactions (ARs) to the vaccine may occur but they are expected to be mainly local and transient. Severe allergic reactions to one of the

vaccine components are unlikely to occur. Boostrix®-IPV booster vaccination in children and adults is a common procedure in a large number of countries already.

Table 2: summary of study objectives and endpoints

	Objectives	Endpoints
Primary	To assess magnitude and changes in pertussis toxin (PT) specific IgG antibody levels just at T0 and T4 in 2 cohorts of children, 1 cohort of young adults and 1 cohort of elderly in three different countries.	The main study parameter is the specific IgG antibody level against PT at T4 using the isolated serum fraction.
Secondary	To assess the PT specific IgG antibody levels at T5.<br><br>To determine pertussis-specific IgG-subclasses and -avidity at T0, T4 and T5.	Anti-PT specific IgG-antibodies in serum and their avidity will be determined for all samples in a PERISCOPE serological core assay, comparing T0, T4 and T5.
	To assess the specific IgG antibody levels against other pertussis vaccine antigens, as well as non-pertussis vaccine antigens at T0, T4 and T5.	Specific IgG-levels to other pertussis vaccine antigens, as well as to non-pertussis antigens will be determined at T0, T4 and T5, using a PERISCOPE serological multiplex immunoassay.
	To determine functional pertussis-specific antibody levels at T0, T4 and T5.	Functional pertussis-specific antibody levels will be determined in serum samples at T0, T4 and T5, using PERISCOPE core assays. Differences in levels of functional antibodies will be measured before and after vaccination. Functional antibody assays include bacterial adherence inhibition (BAI), PT neutralisation (PTNA), serum bactericidal activity (SBA), and bacterial opsonophagocytosis assay (OPA) Other serological parameters such as avidity, subclass distribution (IgA, IgM) are optional in serum samples.
	To assess magnitude and longevity of memory B cell responses against the various *B. pertussis* vaccine proteins at T0, T2, T4 and T5, to determine the effects of an aP booster vaccination in children, young adults and elderly on memory and effector cell populations.	Antigen-specific memory B cell responses at time points T0, T2, T4 and T5 will be measured to determine the effects of aP booster vaccination in children, young adults and elderly, using PERISCOPE B cell core assay.
	To compare pertussis antigen-specific T helper responses and their ratio's at T0, T3, T4 and T5.	Characterisation of the effect of an aP booster on the specific T cell immune response, both early after the boost and later on, in different age groups, vaccinated initially either with a whole cell or an acellular vaccine.

	Collection and biobanking of biological samples for identifying biomarkers of lasting memory or waning of immunity to Pertussis.	Collection and biobanking of biological samples to be used for testing in novel exploratory immunoassays and for possible bridging to other pertussis vaccine studies.
Exploratory	To assess peripheral blood cellular immune-phenotype and soluble mediators, in particular the innate cellular immune responses at T1 (only in children between 11 and 15 years old) and the adaptive cellular immune responses at T2 or T3.	Differences in T cell programming will be measured on fresh blood samples or frozen PBMC samples in an exploratory PERISCOPE T cell assay. This assay includes but is not limited to multi-colour flow cytometry and/or mass cytometry (CyTOF), combined with supernatant cytokine analysis.
	Assessment of genetic and epigenetic determinants of the immune response to aP booster immunisation.	To assess differences in epigenetic imprinting of immune cells by primary vaccination on dTap-IPV booster vaccination, material for epigenetic markers will be isolated before and after vaccination. If differences in (functional) immunological memory are observed, epigenetic markers related to immune function will be analysed.
	Exploration of the relationship of immunogenicity and gene-expression	Gene expression differences in response to dTap-IPV booster vaccination will be measured in immune cells, from before and after vaccination.
	To compare participants within and between the different cohorts in the different countries on different time points before and after vaccination in terms of cellular dynamics, cytokine production, gene transcription, epigenetic changes, B cell (receptor) repertoire and more deeply explored characteristics of the Bp-specific responding T cells.	Intra-individual changes in cell subsets in response to vaccination will be measured on fresh blood samples by flow cytometry (EuroFlow) and/or mass cytometry (CyTOF). Changes in the B cell receptor repertoire will be examined, as this will provide information on the impact of vaccination in different priming backgrounds and ages.<br><br>Cytokine production will be evaluated after *ex vivo* restimulation of PBMCs/whole blood, and potentially also directly in serum, taken before and after vaccination.
	To obtain mucosal lining fluid (MLF) using synthetic absorptive matrices (SAM) in children, young adults and elderly and determine the induction and persistence of mucosal antibodies following a booster vaccination. By measuring mucosal antibodies at T0, T4 and T5.(NL only)	The soluble factors in the eluate from nasal absorption (including cytokines/chemokines) will be analysed by Luminex MIA, BAI and SBA.

1. INTRODUCTION AND RATIONALE

Pertussis, also called whooping cough, is an acute and serious respiratory infection in humans, caused by the Gram-negative bacterium *Bordetella pertussis* (*B. pertussis*). This infection can lead to serious disease and even death, in particular in babies. Since the introduction of whole-cell pertussis (wP) vaccines in the 1940s, widespread vaccination of young children reduced the incidence of pertussis. However, the reactogenicity of the wP vaccines caused public anxiety and consequently withdrawal of these vaccines from the national immunisation programme (NIP) in several European countries, such as Sweden and the United Kingdom (UK). In response to the numerous ARs induced by wP vaccinations, acellular pertussis (aP) vaccines were developed in the late 1970s and 1980s. In vaccine efficacy trials, conducted in the 1980s and the 1990s in Sweden, Germany, Italy and Japan, the aP vaccines were found to be immunogenic, less reactogenic than the wP vaccines and depending on the number of components (2-5) they contained, almost as protective as the wP vaccines. The current aP vaccines contain either 1 component (pertussis toxin (PT)), 2 components (PT and filamentous hemagglutinin (FHA)), 3 components (PT, FHA and pertactin (Prn)) or 5 components (PT, FHA, Prn and fimbriae (Fim2,3)) as purified proteins from *B. pertussis*. According to the World Health Organisation (WHO) guidelines, an aP with at least 3 components should be used. Diphtheria and tetanus toxoid, acellular pertussis vaccines (DTaPs) were licensed alone [1-5], and in combination with inactivated poliovirus vaccine (IPV), Haemophilus influenzae Type B vaccine (Hib) or Hepatitis B vaccine (HepB) resulting in pentavalent or hexavalent vaccines. These aP combination vaccines have been implemented in NIPs, starting in Japan (1982) and subsequently in the United States of America (USA), Canada, Australia and the European countries during the 1990s up to 2005. However, despite the high vaccination coverage in high income countries, pertussis has re-emerged in Europe [6-9], the USA and Canada [10, 11], Australia [12] and Japan [13].

A rise in notifications of pertussis in the Netherlands has been seen since 1996 (Figure 2), with regular epidemic peaks observed every 2 to 3 years [14, 15]. In 2012, the largest epidemic peak occurred since pertussis became a notifiable disease in 1976 in the Netherlands, with more than 13,000 reported cases, including three deaths in unvaccinated neonates between 0-2 months of age [RIVM.nl]. Similarly, in 2012 the UK suffered the highest mortality rate registered since 1982, with 12 deaths, all in children under 12 months of age [16, 17]. However, this was the first severe pertussis epidemic in the UK since the switch to aP vaccines in 2005. In contrast, in Finland there have been very few notifications of pertussis cases in the last ten years since the implementation of aP vaccinations in the primary schedule and booster vaccinations at 4 and 14 years of age. Laboratory confirmed pertussis cases are reported to the national infectious disease register since 1995. Since then outbreaks have occurred in 1999 (918 cases), 2004 (1631 cases) and 2011 (555 cases). From 80 to 90% of the diagnoses

are based on serology. In 2015, the incidence of pertussis in Finland was 3.02 /100,000 and only 11 patients less than one year of age were diagnosed in the whole country. Consequently, the epidemiological situation for pertussis is quite different for these three European countries.

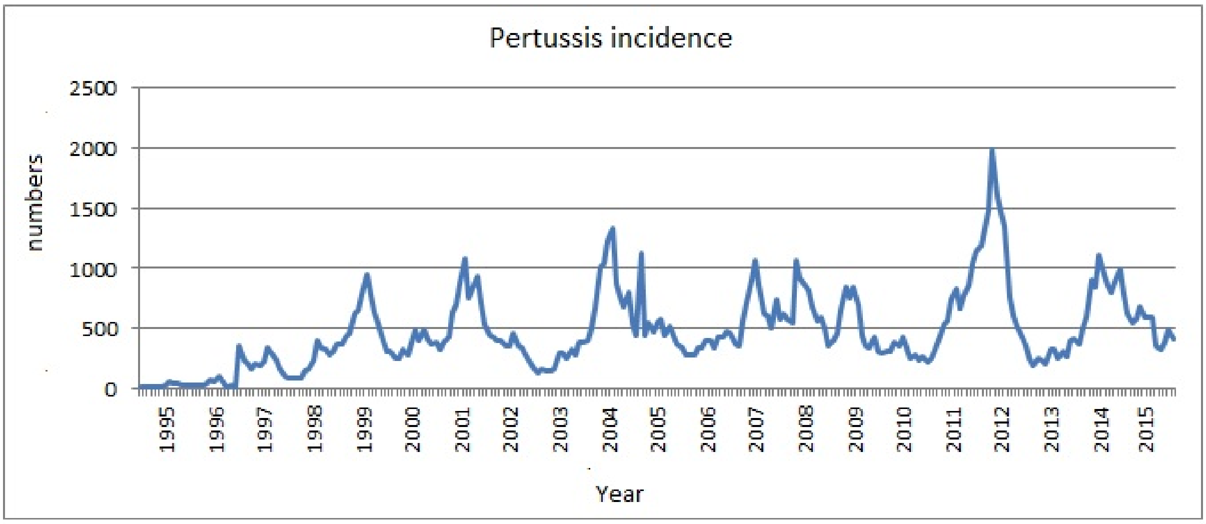

Figure 2: monthly number of pertussis cases in the Netherlands, from 1995 up to 2015

In response to the repeated pertussis epidemics from 1996 onwards in the Netherlands, many changes in the vaccination policy have taken place in the Dutch NIP. First, the vaccination schedule was accelerated from 3, 4 and 5 months to 2, 3 and 4 months of age in 1999 to reduce the period after birth during which neonates are not protected. Next, an aP booster vaccine in children 4 years of age was introduced in 2001, since peak-incidence was highest in this age group at that time and serological studies showed prevalence of very low pertussis antibody levels in 4 years old children [18, 19]. Finally, also under influence of public concern about the ARs associated with wP, from January 1st 2005 the aP vaccine was implemented in the first year of life in the Netherlands instead of wP, a few number of participants (born between May and July 2004), might have a mixed primary schedule with wP and aP vaccines. In the next years, the routinely used aP vaccines changed regularly, containing either 3 components (PT, FHA and Prn) or 5 components (PT, FHA, Prn and Fim2,3). Nowadays, children are vaccinated against pertussis at the age of 2, 3, 4, and 11 months with hexavalent DTaP-IPV-Hib-HepB (Infanrix® hexa) and at 4 years of age with DTaP-IPV (Infanrix®-IPV), both containing 3 pertussis vaccine components (Figure 1).

In the UK, the diphtheria and tetanus toxoid, whole-cell pertussis vaccine (DTwP) was introduced as part of the routine schedule in 1957 [20]. The extended schedule of 3, 4.5-5 and 8.5-11 months of age that was initially recommended, was changed for an accelerated schedule at 2, 3 and 4 months of age, after advice from the Joint Committee on Vaccination and Immunisation in 1990. This recommendation was based on different assumptions: the schedule provided adequate immunogenicity; would allow a quicker protection against the

disease; and seemed to be associated with fewer cases of febrile convulsions [20]. Although the wP was part of the routine schedule in the UK, at the end of 1999, due to shortage of supply of the combined wP vaccine (DTwP-Hib), a 3-component combined aP vaccine (DTaP3-Hib) was used in part of the population during 2000 and 2002 [21-23]. It is estimated that half of the UK population received the DTaP3-Hib during this period, with no specific regions in the country being more affected than others [21]. In October 2001, a booster dose of a 3 or 5-component aP (DTaP3 / DTaP5) was introduced in the schedule for pre-school children, aged between 3.5 and 5 years [23]. In October 2004, the wP was definitively replaced in the UK routine schedule by a 5-component combined aP vaccine [24]. The current UK schedule includes vaccination against pertussis at 2, 3 and 4 months of age (DTaP-IPV-Hib, Pediacel® or Infanrix®-IPV-Hib) with a booster dose at 3 years and four months of age (DTaP-IPV, Infanrix® or Repevax®) [25]. Due to the pertussis outbreak in 2012, the department of health also recommended that all pregnant women should be immunised against pertussis at 28-32 weeks of gestational age. From April 2016, the recommendation was extended to 16-32 weeks. The vaccine was introduced in October 2012, and the recommendation will continue at least until 2019 [24, 25] (Figure 1).

In Finland, the pertussis vaccinations were started in 1952. The primary immunisations were given at 3, 4 and 5 months of age. In 1957, the wP contained $15x10^9$ killed bacteria/ml and a lower dose booster vaccine ($2x10^9$ killed bacteria/ml) was administered at 3-4 and 6-7 years of age. The schedule was changed to 3, 4, 5 and 24 months of age in 1977. In 1962-1976, the vaccines contained only one serotype (18530, Fim 3) but in 1976, half of the vaccine was replaced by serotype Fim 2,3. All wP vaccines have been produced at the National Public Health Institute in Finland since 1970s. In 2003 an aP booster dose was started for children at six years of age and in 2005 the primary schedule was changed to 3, 5, 12 months of age with aP vaccine. Booster vaccinations were given at 4 years and 14 years of age. In 2005-9, the aP vaccine was a 2-component vaccine (PT and FHA) (Pentavac) In 2009, it was replaced by a 3-component vaccine (PT, FHA and Prn) (Infanrix®-IPV-Hib). The booster at 4 years of age is still a 2-component vaccine (PT and FHA) (Tetravac) and at 14 years of age a 3-component vaccine (Boostrix®). During 2012, an adult booster (Boostrix®) was started in army recruits (almost all men and some women between 18 to 30 years of age). Very quickly, pertussis almost disappeared in that adult population.

Evidence is accumulating that the pertussis incidence in adolescents, adults and elderly in the last decennium has increased. In large population-based serosurveillance studies conducted by the RIVM in the Netherlands in 1995/6 and 2006/7, the percentage of individuals above 9 years of age with an IgG-PT concentration above 62.5 ELISA units/ml, indicative for a recent pertussis infection, more than doubled (4.0 and 9.3%, respectively). The vast majority of these pertussis cases are not notified, indicating that adults infected with pertussis do not generally

visit the general practitioner (GP) for counselling, or that the course of infection is mostly subclinical [26, 27].

In 2015, in England, 4190 cases of laboratory confirmed pertussis were reported, 55% lower than during the outbreak in 2012, but 24% higher than in 2014. In 2015, the number of confirmed cases in children between 5-9 years of age was 218, the highest number since 1994, when an enhanced surveillance for this disease was introduced in the UK. For children aged 10-14 years, 437 cases were confirmed, the highest number, since the outbreak of 2012. The serology confirmed cases (IgG anti-PT) during this year were 90% of the total of laboratory confirmed cases. From all confirmed cases, 94.7% were at least ten years of age [28].

Estimates from the WHO in 2008 suggest that about 16 million cases of pertussis occurred worldwide, with about 195,000 children dying each year from the disease, mostly in low-income countries. To reduce the pertussis burden, several countries have recommended adult pertussis booster vaccination: in the USA, Canada, France and Germany one of the reduced diphtheria toxoid and tetanus toxoid vaccines (dT) administered every 10 years has been replaced by reduced diphtheria toxoid, tetanus toxoid and reduced acellular pertussis vaccine (dTap). In Austria, a reduced diphtheria toxoid, tetanus toxoid and acellular pertussis vaccine (dTap)-IPV should be given every 10 years to adults [29-32]. However, the coverage of these adult vaccinations is poor (2-10%).

Several possible explanations for the re-emergence of pertussis in highly vaccinated countries have been proposed, such as improved diagnostics, increased reporting, *B. pertussis* strain variation, changes in vaccine composition and waning immunity in a community where only infants and children are vaccinated [15, 33, 34]. The major change in the pertussis vaccination schedules in the last 20 years is the switch from wP vaccines to aP vaccines. Although antibody levels are higher after aP vaccination than with wP [18, 35], they wane relatively quickly in the first years after vaccination [35-37] resulting in a reduced immunity against pertussis. The prevalence of specific antibodies against PT, Prn and Fim could be related to protection against whooping cough [38-40], but the presence of antibodies does not always confer protection against infection or disease [36, 37, 41-43]. From the epidemics in the USA in 2010 and 2012, it became clear that, although the aP vaccines provide good protection in the first years of life, the protection is not long lasting after the fifth booster vaccination at 4-6 years of age. Children 8-12 years of age and primed with wP vaccines seemed to be better protected than aP primed children [44]. In addition, in the baboon model aP vaccines protected against disease but could not prevent transmission [45]. In contrast, both vaccination with wP or previous infection with *B. pertussis* conferred protection to infection. Evidence is accumulating that the aP vaccines induce a more Th2 skewed cellular response, resulting in a shorter duration of immunity compared to wP vaccines and natural infection that induce a more Th1/Th17-skewed cellular response in young children [43].

Waning immunity to wP and especially to aP following primary immunisation and boosting is thought to contribute to sustained transmission of *B. pertussis* in the population. Strategies for controlling disease include additional booster immunisation in older children and adults. aP booster vaccination seemed to induce a better persistence of pertussis specific antibodies in wP-primed adults in the APERT-study than those primed by aP [27, 46]. Results from booster studies in young children (aP and wP primed) and adults (wP primed) in the Netherlands corroborate this finding [42], personal communication Van der Lee et al,] resulting in higher antibody levels and memory B cells after 1 year. These prolonged antibody levels may indirectly help protect vulnerable infants because vaccinated parents are less likely to transmit the disease to their infants [47].

In this clinical study, we aim to characterise the immunological response to a booster vaccination in different age groups and in different priming backgrounds more thoroughly. We aim to generate insight on the effect of aP boosting on the duration of immunity conferred following primary schedules using aP (7-10 and 11-15 year olds) and wP vaccines (11-15, 20-34 and 60-70 year olds). We will analyse innate and humoral/cellular immune responses following immunisation to provide a detailed understanding of immunity to aP and to assess biomarkers that can be used to detect early signs of the waning of immunological memory to pertussis and biomarkers that can be used to assess long lasting immunological memory to pertussis.

Participants will be recruited across highly experienced trial sites in the UK, Finland and the Netherlands, allowing comparison between different epidemiological and priming backgrounds for pertussis in the three countries.

In conclusion, this study aims to investigate the effects of an aP booster vaccination in children, young adults and elderly with different epidemiological and priming backgrounds for pertussis on the magnitude, quality and persistence of immunological memory against *B. pertussis*.

2. OBJECTIVES

The main objective of this study is to investigate the effects of an aP booster vaccination in children, young adults and elderly, who have been primed with different pertussis vaccines (aP vs. wP), on the magnitude, quality and persistence of immunological (long-term) memory against *B. pertussis*. Intra-individual changes in the immune response will be assessed both early after vaccination, as well as at later time points to measure the persistence of specific memory against *B. pertussis*.

2.1 Primary objective

To assess magnitude and changes in pertussis toxin (PT) specific IgG antibody levels just before (T0) and 28 days (T4) after the booster vaccination in 2 cohorts of children (7-10 and 11-15 years of age), 1 cohort of young adults (20-34 years of age) and 1 cohort of elderly (60-70 years of age) in three different countries.

2.2 Secondary objectives

- To assess the PT specific IgG antibody levels 1 year (T5) after vaccination.
- To assess the specific IgG antibody levels against other pertussis vaccine antigens (e.g. FHA, Prn and Fim2,3), as well as non-pertussis vaccine antigens (e.g. diphtheria toxoid, tetanus toxoid) at T0, T4 and T5.
- To determine pertussis-specific IgG-subclasses and -avidity at T0, T4 and T5.
- To determine functional pertussis-specific antibody levels at T0, T4 and T5.
- To assess magnitude and longevity of memory B cell responses against the various *B. pertussis* vaccine proteins at T0, as well as 7 days post vaccination (T2), T4 and T5, to determine the effects of an aP booster vaccination in children, young adults and elderly on memory and effector cell populations.
- To compare pertussis antigen-specific T helper responses and their ratio's at T0, as well as 14 days post vaccination (T3), T4 and T5.
- Collection and biobanking of biological samples for identifying biomarkers of lasting memory or waning of immunity to Pertussis.

2.3 Exploratory objectives

- To assess peripheral blood cellular immune-phenotype and soluble mediators, in particular the innate cellular immune responses at day 1 (T1) (only in children between 11 and 15 years old) and the adaptive cellular immune responses at T2 or T3.

- Assessment of genetic and epigenetic determinants of the immune response to aP booster immunisation.
- Exploration of the relationship of immunogenicity and gene-expression
- To compare participants within and between the different cohorts in the different countries on different time points before and after vaccination in terms of cellular dynamics, cytokine production, gene transcription, epigenetic changes, B cell (receptor) repertoire and more deeply explored characteristics of the Bp-specific responding T cells.
- To obtain mucosal lining fluid (MLF) using synthetic absorptive matrices (SAM) in children, young adults and elderly and determine the induction and persistence of mucosal antibodies following a booster vaccination. By measuring mucosal antibodies at T0, T4 and T5.

3. STUDY DESIGN

This is an interventional, longitudinal, open-label study in four different age cohorts across three European Union (EU) countries. For this purpose, cohorts of children (cohort A: from 7 up to 10 and cohort B: from 11 up to 15 years old), young adults (cohort C: from 20 up to 34 years old) and elderly (cohort D: from 60 up to 70 years old) will be recruited.

3.1 Vaccination and collection of blood samples

The total duration of this study is one year and comprises five visits (for adults) or four visits (for children) at a study location in the vicinity of the participant's home, or visits from the investigator's team at the participant's home address. From all participants, blood samples will be obtained at the first visit before (T0), as well as at 28 days (T4) and 1 year (T5) after vaccination. After the blood draw at T0, a single dose of the Boostrix®-IPV combination vaccine (GSK) will be administered.

For the adult cohorts (C and D), additional blood samples will be drawn from all participants at each of the following time points after vaccination: 7 days (T2) and 14 days (T3). Thus, adults will be asked for five blood draws with a cumulative volume of maximum 180 mL blood over one year. To reduce the burden for the children and minimise the total amount of blood drawn over the first 14 days of the study, cohorts A and B will be divided into subcohorts, each of which will have one additional blood draw respectively at T2 or T3 and at T1, T2 or T3. Therefore, children will be asked to donate blood 4 times with a cumulative volume for cohort A of maximum 62 mL, and cohort B of maximum 102 mL. For all cohorts MLF samples will be taken at T0, T4 and T5. In the PERISCOPE consortium, the different assays are divided in three different categories: core assays, pre-core assays and exploratory assays. Core assays are assays focussing on the primary and secondary endpoint. Pre-core assays are highly standardised assays focused on the exploratory endpoint, and exploratory assays are experimental and focused on the exploratory endpoints. The serum Core assays will be performed on the samples from all participants. Cellular CORE assays will be performed on a selection of the participants. Pre-core and exploratory assays will also be performed only on a selection of the biological samples. Some assays need different blood volumes, causing the differences in blood volumes within one time point. See for an overview of the maximum amounts of blood taken at each visit Table 3 and for a detailed sample plan APPENDIX C: Detailed sample plan.

The time window for sampling at T1 will be 4 hours, at T2 will be 1 day, at T3 will be ± 2 days and at T4 will be ± 4 days, but is to be kept as short as possible. The time window

for sampling at T5 will be ± 4 weeks. See Table 1 and Figure 3 for an overview of the total study schedule.

Table 3: Sample plan

S: serologic core assay; **B^{core}**: B cell core assay; **T^{core}**: Rapid T cell core assay; **EF**: EuroFlow pre-core assay; **Gx**: RNA sequencing exploratory assay; **Cyt**: innate cytometry by time of flight (CyTOF) exploratory assay; **scG**: single cell RNA gene expression exploratory assay; **B^{exp}**: exploratory B cell assays; **T^{exp}**: exploratory in depth T cell assay; **MLF**: exploratory assay - mucosal lining fluid, *: only performed in the Netherlands.

**Time points**		**T0**	**T1**	**T2**	**T3**	**T4**	**T5**
Age	**cohort**	Baseline	1 day post vaccination	7 days post vaccination	14 days post vaccination	28 days post vaccination	1 year post vaccination
7-10 y	**A1**	**16 mL**<br>**S, B^{core}, EF, B^{exp}, MLF***		**14 mL**<br>**B^{core}, EF, B^{exp}**		**16 mL**<br>**S, B^{core}, EF, B^{exp}, MLF***	**16 mL**<br>**S, B^{core}, B^{exp}, MLF***
	**A2**	**16 mL**<br>**S, T^{core}, EF, T^{exp}, MLF***			**14 mL**<br>**T^{core}, EF, T^{exp}**	**16 mL**<br>**S, T^{core}, EF, T^{exp}, MLF***	**16 mL**<br>**S, T^{core}, T^{exp}, MLF***
11-15 y	**B1**	**26 mL**<br>**S, B^{core}, T^{core}, EF, Gx, Cyt, scG, MLF***	**24 mL**<br>**EF, Gx, Cyt, scG**			**26 mL**<br>**S, B^{core}, EF, T^{core}, MLF***	**26 mL**<br>**S, B^{core}, T^{core}, MLF***
	**B2**	**26 mL**<br>**S, B^{core}, EF, B^{exp}, MLF***		**24 mL**<br>**B^{core}, EF, B^{exp}**		**26 mL**<br>**S, B^{core}, EF, B^{exp}, MLF***	**26 mL**<br>**S, B^{core}, B^{exp}, MLF***
	**B3**	**26 mL**<br>**S, T^{core}, EF, T^{exp}, MLF***			**24 mL**<br>**T^{core}, EF, T^{exp}**	**26 mL**<br>**S, T^{core}, EF, T^{exp}, MLF***	**26 mL**<br>**S, T^{core}, T^{exp}, MLF***
20-34 y	**C**	**40 mL**<br>**S, B^{core}, T^{core}, EF, B^{exp}, T^{exp}, MLF***		**20 mL**<br>**B^{core}, T^{core}, EF, B^{exp}**	**40 mL**<br>**B^{core}, T^{core}, EF, B^{exp}, T^{exp}**	**40 mL**<br>**S, B^{core}, T^{core}, EF, B^{exp}, T^{exp}, MLF***	**40 mL**<br>**S, B^{core}, T^{core}, B^{exp}, T^{exp}, MLF***
60-70 y	**D**	**40 mL**<br>**S, B^{core}, T^{core}, EF, B^{exp}, T^{exp}, MLF***		**20 mL**<br>**B^{core}, T^{core}, EF, B^{exp}**	**40 mL**<br>**B^{core}, T^{core}, EF, B^{exp}, T^{exp}**	**40 mL**<br>**S, B^{core}, T^{core}, EF, B^{exp}, T^{exp}, MLF***	**40 mL**<br>**S, B^{core}, T^{core}, B^{exp}, T^{exp}, MLF***

3.2 Study sites

In the Netherlands, Spaarne Hospital will be the study site. To prevent participants from traveling a long distance and due to the high frequency of samplings in the first week, participants will be visited at home. The time of vaccination during the day will be synchronised as much as possible.

In the UK, potentially eligible children whose parents are interested in the study will be visited at their home by the paediatric study team. If local schools from Thames Valley agree, the study visits could be done in school facilities, during school time, after school activities. For the adult population, the study visits will be performed at the study site by the adult team, at the Oxford Vaccine Group (OVG) – Centre for Clinical Vaccinology and Tropical Medicine, University of Oxford, based at the Churchill Hospital.

In Finland the study site is Turku University Hospital. The participants will be seen there and no home visits are planned. The clinical study site and the study laboratory are very close to each other at the university campus.

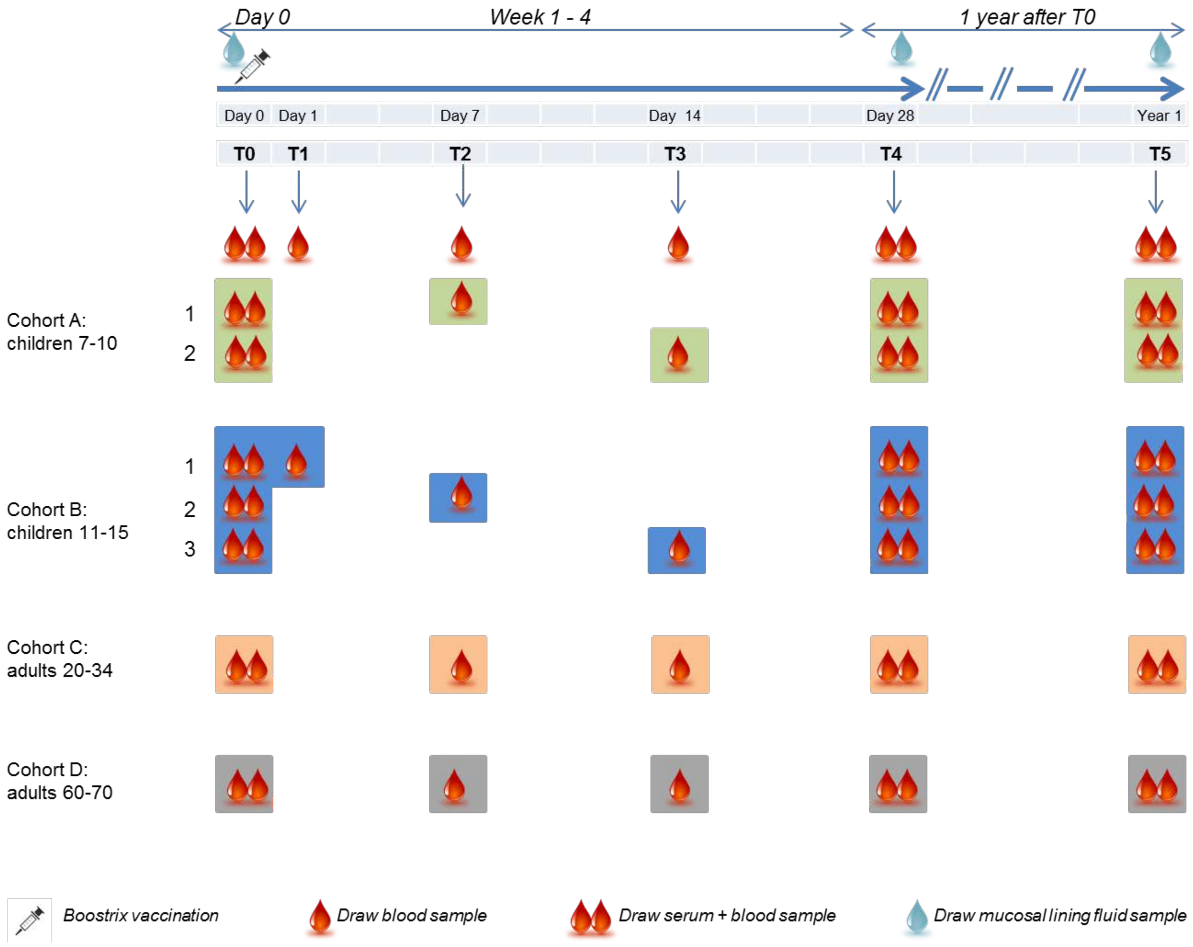

Figure 3: study schedule

4. STUDY POPULATION

4.1 Population

Participants for the four cohorts will be recruited for this study:

- cohort A: children born between 2007 and 2010, i.e. from 7 up to 10 years of age. Male + female, approximately equally distributed, n = 36 in each country;
- cohort B: children born between 2002 and 2006, i.e. from 11 up to 15 years of age Male + female, approximately equally distributed, n = 36 in each country aiming for comparable numbers of participants with aP vs wP vaccination background;
- cohort C: young adults born between 1983 and 1997, i.e. from 20 up to 34 years of age. Male + female, approximately equally distributed, n = 25 in each country;
- cohort D: elderly born between 1947 and 1957, i.e. from 60 up to 70 years of age. Male + female, approximately equally distributed, n = 25 in each country.

4.2 Inclusion criteria

In order to be eligible to participate in this study, participants must meet all of the following criteria:

- normal general health;
- within the right age group for the cohort;
- received all regular vaccines for their age group according to the Dutch NIP, UK NIP or Finnish NIP; a copy of the vaccination booklet will be included in the participant's documents. If booklet is not available for cohorts A, B and C, vaccination status will be checked with regulatory agencies / GP. For cohort D this booklet might not be available due to their age;
- provision of written informed consent (see section 11.2 for details);
- willing to adhere to the protocol and be available during the study period.

4.3 Exclusion criteria

Any of the following criteria will exclude a participant from this study:

- present evidence of serious disease(s) within the last 3 months before inclusion requiring immunosuppressive or immune modulating medical treatment, such as systemic corticosteroids, that might interfere with the results of the study ;
- chronic infection
- known or suspected immune deficiency;
- history of any neurologic disorder, including epilepsy;

- previous administration of serum products (including immunoglobulins) within 6 months before vaccination and blood sampling;
- known or suspected allergy to any of the vaccine components (by medical history);
- occurrence of serious adverse events (SAEs) after primary DTwP-IPV vaccination, DTaP-IPV vaccination or any other vaccination (by medical history);
- vaccination with any pertussis vaccine other than those described in the inclusion criteria (i.e. only according to NIP);
- vaccination with any other DT-IPV vaccine in the last 5 years, a DT-IPV vaccination according to the NIP in cohort B is no exclusion criterion;
- children between 8 and 10 years of age eligible for cohort A in the Netherlands who have already received the diphtheria and tetanus toxoid vaccine (DT)-IPV booster vaccination according to the Dutch NIP around 9 years of age;
- mixed wP and aP priming within a participant, cohort B;
- Pregnancy. Detailed considerations for this exclusion criterion in section 4.6.

4.4 Delay criteria

- If a participant has a severe acute (infectious) illness or fever (>38°C) within 14 days prior to T0, participation will be postponed or cancelled. In case the participant has fever within 2 days before sampling at T4 or T5, the appointment will be postponed for 4 days, if possible.
- Antibiotic use within 14 days of enrolment.
- Any vaccination within a month before enrolment.

4.5 Collection of information on immunisation status

NL: during telephone screening eligible participants/parents/legal guardians will be asked if they/their child was vaccinated according the NIP and if they/their child has had other vaccination outside NIP.

participants/parents/legal guardians are asked to keep their vaccination booklets at hand for the first visit. If participants/parents/legal guardians do not have a vaccination booklet anymore permission is asked to contact DVP to check vaccination status according to NIP.

4.6 Pregnancy

The study vaccine (Boostrix®-IPV) is recommended routinely from 16 weeks gestation in pregnancy as described in the Green Book: immunisation against infectious disease (chapter 24). Pregnancy is an exclusion criterion in the current study in order to reduce

heterogeneity in the immune response within a group. The possibility of pregnancy is absent in group A (7-10 years of age) and low in groups B and D (11-15 year of age and 60-70 years of age respectively).Therefore for groups B and D medical history alone will be used to establish the absence of this criterion. For group C in view of the higher probability of pregnancy, a urine pregnancy test will be performed prior to any vaccination, that is, the day of vaccination (T0).

4.7 Sample size calculation

Across our trial sites in the UK, Finland and the Netherlands participants will be recruited as follows: 108 individuals in each of the 7-10y (aP primed in early childhood) and 11-15y cohorts (approximately half primed with aP and half wP); and 75 in each of 20-34y and 60-70y cohorts (both wP primed). In total 366 participants will be enrolled together in the three countries, per child cohort in each country 36 participants and per adult cohort 25 participants.

A sample size of 108 per child cohort provides 80% power to detect a standardised difference in log-anti-PT IgG at one month post booster of 0.42 between age cohorts, allowing 15 % loss to follow up/sample loss. For the smaller adult cohorts, analyses will focus on novel and exploratory assays. The data obtained from the participants in each country will be combined and analysed per cohort.

5. TREATMENT OF PARTICIPANTS

5.1 Investigational product/treatment

All participants will receive one dose of aP booster vaccine consisting of 3 pertussis vaccine components together with dT and IPV (Boostrix®-IPV) (Dutch authorisation number RVG 35123). Blood will be drawn 3-4 times in the first 4 weeks after vaccination, and once 1 year after vaccination.

5.2 Use of co-intervention

Not applicable.

5.3 Escape medication

Epinephrine autoinjector (EpiPen) in case of acute anaphylactic reaction.

6. INVESTIGATIONAL PRODUCT

6.1 Name and description of investigational product

Boostrix®-IPV (dTap-IPV, GSK, Dutch authorisation number RVG 35123) is a combination vaccine. It contains at least 2 international units (IU) of diphtheria toxoid[1], at least 20 IU of tetanus toxoid[1] 8 micrograms (µg) of pertussis toxoid[1], 8 µg FHA[1] and 2.5 µg Prn[1]. It also contains D-antigen units of different IPV strains: 40 D-antigen units of type 1 (Mahoney strain)[2], 8 D-antigen units of type 2 (MEF-1 strain)[2], and 32 D-antigen units of type 3 (Saukett strain)[2]. In addition, the vaccine contains sodium chloride and water.

[1] adsorbed on hydrated aluminium hydroxide ($Al(OH)_3$) (0.3 mg Al^{3+}) and aluminium phosphate ($AlPO_4$) (0.2 milligram Al^{3+})

[2] propagated in VERO cells

Boostrix®-IPV is authorised in the Netherlands under authorisation number RVG 35123 since September 1992.

UK: Boostrix®-IPV under authorisation number PL 10592/0214 since December 2008.

Finland: Boostrix®-IPV under authorisation number 19479 since March 2009.

6.2 Summary of findings from non-clinical studies

Summary of findings from non-clinical studies can be found in section 5.3 of the Boostrix®-IPV summary of product characteristics (SmPC).

6.3 Summary of findings from clinical studies

Summary of findings from clinical studies can be found in section 5.1 of the Boostrix®-IPV SmPC.

6.4 Summary of known and potential risks and benefits

Summary of known and potential risks and benefits can be found in section 4.3, 4.4, 4.5, 4.6, 4.7, 4.8, 4.9 and 5.1, 5.2 and 5.3 of the Boostrix®-IPV SmPC.

Boostrix®-IPV is used as authorised in this study and therefore poses no additional risk. Participants may benefit from boosting of their immune response.

6.5 Description and justification of route of administration and dosage

Boostrix®-IPV vaccine (0.5 ml) is administered by intramuscular injection in the deltoid muscle of the upper arm. See section 4.2 of the Boostrix®-IPV SmPC.

6.6 Dosages, dosage modifications and method of administration

Participants will receive one intramuscular injection with a single dose (0.5 ml) of the Boostrix®-IPV vaccine.

6.7 Preparation and labelling of Investigational Medicinal Product

The vaccine will be provided in single-dose prefilled syringes containing 0.5-ml suspension for injection. The vaccine should be stored refrigerated between 2ºC and 8ºC. If the vaccine has been frozen, it should be discarded.

Prior to use, the vaccine should be at room temperature, and well shaken in order to obtain a homogeneous turbid white suspension. Prior to administration, the vaccine should be visually inspected for any foreign particulate matter and/or variation of physical aspect. In the event of either being observed, the vaccine should be discarded.

See section 6.4 and 6.6 of the Boostrix®-IPV SmPC.

6.8 Drug accountability

The Boostrix®-IPV vaccine will be received from GSK and distributed to the participating clinical study sites on behalf of the PERISCOPE project by OTC Direct, Clinical Trial Services (Surrey, UK). One vaccine batch will be used for all sites if possible. The vaccines will be distributed to the study sites while maintaining the cold chain 2°C to 8°C. The investigator is responsible for adequate storage and drug accountability at the study site.

Each vaccine delivery will be documented by a dated and signed receipt form, containing information about the quantity, expiry date and batch numbers of the supplied vaccines.

Records will be maintained to document that the vaccines are administered to study participants as specified by the protocol. At all times the number of used doses must be reconciled with the number of vaccines received.

If transport of the vaccine is required from the study site depository to a location where the vaccine is administered, this will be done in insulated containers under continuous monitoring of the temperature.

All unused vaccines will be either returned to the distributor or destroyed, either of which will be properly documented.

7. NON-INVESTIGATIONAL PRODUCT

Children in group A and B will be offered a local anaesthetic patch as a local anaesthetic of the skin before venepuncture.

7.1 Name and description of non-investigational product(s)

Rapydan (Eurocept International BV, Dutch authorisation number RVG 100315 since 17 December 2007) is a local anaesthetic patch containing 70 mg lidocaine and 70 mg tetracaine and can be used as a local anaesthetic of the skin.

UK: Rapydan under authorisation number PL 35068/0001 since 26 January 2012

Finland: Velocaine under authorisation number

7.4 Summary of known and potential risks and benefits

Summary of known and potential risks and benefits can be found in section 4 of Rapydan SmPC.

7.5 Description and justification of route of administration and dosage

Rapydan patches are applied at dry skin, one at each elbow at the beginning of the visit for 30 minutes.

7.6 Dosages, dosage modifications and method of administration

On request of the participant 2 patches will be used.

8. METHODS

8.1 Study endpoints

Blood samples (all sites) and MLF samples (NL only) will be collected from all participants prior to vaccination (T0), and after vaccination at T4 and T5. Blood samples will be collected at T1, T2 and T3, according to the sampling schedule described in Table 1 and Figure 3. After collection, blood samples will be kept at room temperature and will be processed either for direct analyses requiring fresh material or for storage prior to further use and analysis (serum at -80 °C, peripheral blood mononuclear cells (PBMCs) at -135 °C, respectively) according to the study's standard operating procedures (SOPs). Frozen samples will be temporarily stored at the central PERISCOPE biobank location at Radboud University Medical Center in Nijmegen, the Netherlands for later distribution, use and analysis (see biobank information in section 12.2). Within PERISCOPE, a number of core assays will be developed in a standardised manner to allow comparison of immune responses to *B. pertussis* across the different clinical studies. These include antibody-based assays, but also core T/B cell assays. In addition, a number of exploratory analyses will be performed to obtain more in-depth knowledge about the immune response to vaccination.

8.1.1 Main study endpoint

The main study parameter is the specific IgG antibody level against PT at T4 using the isolated serum fraction.

8.1.1.1 Pertussis toxin specific IgG antibody concentration and avidity in serum

Anti-PT specific IgG-antibodies in serum and their avidity will be determined for all samples in a PERISCOPE serological core assay, comparing T0, T4 and T5.

8.1.2 Secondary study endpoints

8.1.2.1 Specific IgG levels in serum

Specific IgG-levels to other pertussis vaccine antigens (FHA; Prn and Fim2,3), as well as to non-pertussis antigens (diphtheria toxoid and tetanus toxoid) will be determined at T0, T4 and T5, using a PERISCOPE serological multiplex immunoassay.

8.1.2.2 Other serological endpoints

Functional pertussis-specific antibody levels will be determined in serum samples at T0, T4 and T5, using PERISCOPE core assays as described in section 8.3.4.

Differences in levels of functional antibodies will be measured before and after vaccination. Functional antibody assays include bacterial adherence inhibition (BAI), PT neutralisation (PTNA), serum bactericidal activity (SBA), and bacterial opsonophagocytosis assay (OPA) (Table 3, APPENDIX C: Detailed sample plan). Other serological parameters such as avidity, subclass distribution (IgA, IgM) are optional in serum samples [48, 49].

8.1.2.3 Cellular endpoints

- Antigen-specific memory B cell responses at time points T0, T2, T4 and T5 will be measured to determine the effects of aP booster vaccination in children, young adults and elderly, using PERISCOPE B cell core assay.
- Characterisation of the effect of an aP booster on the specific T cell immune response, both early after the boost and later on, in different age groups, vaccinated initially either with a whole cell or an acellular vaccine.

8.1.2.4 Logistic endpoints

- Collection and biobanking of biological samples to be used for testing in novel exploratory immunoassays and for possible bridging to other pertussis vaccine studies.

8.1.3 Exploratory study endpoints

- Intra-individual changes in cell subsets in response to vaccination will be measured on fresh blood samples by flow cytometry (EuroFlow) and/or mass cytometry (CyTOF).
- Cytokine production will be evaluated after *ex vivo* restimulation of PBMCs/whole blood, and potentially also directly in serum, taken before and after vaccination.
- Gene expression differences in response to dTap-IPV booster vaccination will be measured in immune cells, from before and after vaccination.
- Differences in T cell programming will be measured on fresh blood samples or frozen PBMC samples in an exploratory PERISCOPE T cell assay. This assay includes but is not limited to multi-colour flow cytometry and/or mass cytometry (CyTOF), combined with supernatant cytokine analysis.
- Changes in the B cell receptor repertoire will be examined, as this will provide information on the impact of vaccination in different priming backgrounds and ages.
- The soluble factors in the eluate from nasosorption (including cytokines/chemokines) will be analysed by Luminex multiplex immunoassay

- To assess differences in epigenetic imprinting of immune cells by primary vaccination on dTap-IPV booster vaccination, material for epigenetic markers will be isolated before and after vaccination. If differences in (functional) immunological memory are observed, epigenetic markers related to immune function will be analysed.

8.2 Randomisation, blinding and treatment allocation

In general, this is not applicable since all participants receive the same, open-label vaccination. All children (cohorts A and B) will be sampled at time points T0, T4 and T5. Participants in cohorts A and B will be non-randomised allocated into subcohorts; inclusion in the different subcohorts will be parallel to ensure equal amounts of participants per subcohort, taking into account individual availability and capacity of the laboratories. This will be managed and recorded using a visit calculator. In cohort B, each subcohort will exist half of aP primed, and half of wP primed participants. Each subcohort will have one additional blood sampling time point, cohort A at T2 or T3, and cohort B at T1, T2 or T3. All adults will be sampled at time points T0, T2, T3, T4 and T5.

8.3 Study procedures

See study schedule in section 3.

8.3.1 Detailed description of study visits

Invitation and enrolment.

See section 11.2 for detailed information.

8.3.1.1 Visit T0

Baseline, all participants

- Check if participants information (PIF) was received and is understood
- Answer questions of the participants/parents/guardians regarding any subject of the study
- Obtain informed consent
- Check in-/exclusion criteria
- Record demographic information
- Record medical/vaccination history.
- Record co-medication
- Collect blood by venepuncture
- Collect MLF by nasal absorption (NL only)

- Vaccination with Boostrix®-IPV
- Observe participant for 15 minutes immediately after vaccination
- Record observed AE's
- Verify date and time next visit or make an appointment for a next visit

8.3.1.2 *Visit T1*

1 day after vaccination children cohort B1

- Answer questions of the participants/parents/guardians regarding any subject of the study
- Ask for/record adverse events (AEs)
- Record co-medication
- Collect blood by venepuncture
- Verify date and time next visit or make an appointment for a next visit

8.3.1.3 *Visit T2*

7 days after vaccination all adults, children cohort A1 and B2

- Answer questions of the participants/parents/guardians regarding any subject of the study
- Ask for/record AEs
- Record co-medication
- Collect blood by venepuncture
- Verify date and time next visit or make an appointment for a next visit

8.3.1.4 *Visit T3*

14 days after vaccination all adults, children cohort A2 and B3

- Answer questions of the participants/parents/guardians regarding any subject of the study
- Ask for/record AEs
- Record co-medication
- Collect blood by venepuncture
- Verify date and time next visit or make an appointment for a next visit

8.3.1.5 *Visit T4*

28 days after vaccination all participants

- Answer questions of the participants/parents/guardians regarding any subject of the study
- Ask for/record AEs

- Record changes in medical/vaccination history.
- Record changes in co-medication
- Collect blood by venepuncture
- Collect MLF by nasal absorption (NL only)
- Verify date and time next visit or make an appointment for a next visit
- Hand over reimbursement

8.3.1.6 *Visit T5*

1 year after vaccination all participants

- Answer questions of the participants/parents/guardians regarding any subject of the study
- Record changes in medical/vaccination history.
- Record changes in co-medication
- Collect blood by venepuncture
- Collect MLF by nasal absorption (NL only)
- Vaccination with mumps, measles and rubella vaccine (MMR) (cohort A, only in NL, according to the Dutch NIP)
- Observe participant for 15 minutes immediately after vaccination (cohort A, only in NL)
- Hand over reimbursement

8.3.2 Procedures for sample collection, labelling, processing and storage

All samples collected and processed during the trial will be individually labelled with a unique participant number and a bar code that is linked to the participant, country and study visit.

Blood samples

Freshly collected blood samples will be used directly for immune analysis, or processed in serum and PBMCs, and stored in freezers at -80°C and -135°C respectively, until further testing.

Mucosal lining fluid

Nasal absorption is performed by maneuvering a strip of synthetic absorptive matrices (SAM) up the lumen of the nostril, avoiding rubbing against the nasal mucosa. The outside of the nose is then pressed with a finger to cause apposition of the SAM against the mucosa. The procedure may tickle slightly but is painless, and MLF can be obtained even from non-inflamed noses at frequent intervals, without the need for

local anaesthetic. Imperial College London has performed successful studies with nasal strips in adults after nasal allergen challenge (NAC), in babies and in young children. There is minimal protein binding to the SAM strip, and fluid can be eluted by spin filtration. High levels of mediators of inflammation can then be measured in the MLF: higher than detectable by nasal lavage.

Freshly collected MLF samples will be used directly for immune analysis, or processed and stored in freezers at -80°C until further testing.

8.3.3 Laboratory tests (primary objective)

Fluorescent-bead-based multiplex immunoassay for IgG levels, subclasses, and avidity

In the fluorescent-bead-based multiplex immunoassay (MIA), each antigen of interest is coupled to beads with a distinct fluorescence. Combination of these beads enables the detection of antibodies directed against multiple antigens in one single serum sample, using Luminex technology. IgG-PT antibody concentrations as primary outcome will be measured in duplicate in two dilutions using an in-house standard, calibrated on the WHO reference serum for pertussis as standard expressed in IU/ml, to determine the effects of an adult aP booster vaccination.

Using different conjugates, IgG-total, IgG-subclasses and -avidity, IgA-specific antibodies concentrations can be measured in serum with the MIA. Besides anti-pertussis specific antibody concentrations, the other vaccine-specific IgG-antibody concentrations of diphtheria and tetanus will be measured with MIA in two dilutions using the national reference serum (IU/ml) as standard, which has been calibrated against the WHO standard [50-52].

8.3.4 Other laboratory tests

Functional antibody responses (secondary objectives)

Vaccination-induced antibodies play an important role in the protection against infection with *B. pertussis* (Bp). Antibodies can provide protection in many distinct ways. These include opsonising Bp followed by complement-mediated killing, but also by neutralising the biological activity of e.g. PT. In this study, we intend to assess the functionality of vaccination-induced antibodies in various biological assays. These functional assays are an essential part of the PERISCOPE consortium and will be established as core standardised immunoassays. These *in vitro* assays include, but are not limited to, measuring the ability of antibodies to inhibit adherence of Bp to respiratory epithelial cells, the ability of antibodies to induce bacterial aggregation, and the ability of antibodies to neutralise specific virulence factors of Bp (including

PT). In addition, we will assess the ability of the vaccination-induced antibodies to bind to Bp and induce complement-mediated killing (serum bactericidal assay), or to lead to uptake and killing by phagocytic cells.

Memory B and T cell responses (secondary and exploratory objectives)

Serum antibodies have been the most studied potential correlate of protection. However, various studies have also pointed to an important role for memory B and T cell responses and there is some evidence that differences in priming (e.g. wP versus aP) can affect the T and B cell response to booster vaccination [42,43]. To gain more in-depth insight into how antigen-specific B and T cells are induced and maintained following pertussis vaccination, as well as how these cells may respond to (re)exposure to Bp, we will perform a number of analyses.

- Memory B cell responses to Bp will be analysed by polyclonal stimulation of B cells by using frozen PBMCs in combination with pertussis (protein)-specific enzyme-linked immunospot (ELISpot) assays. The ELISpot is a sensitive immunoassay to enumerate antigen specific antibody-secreting cells (ASCs) [43]. All samples of the same participant will be measured in the same assay to prevent inter-assay variation.
- In parallel, we will characterise the overall and antigen-specific B cell response to vaccination in detail by flow cytometry and/or mass cytometry.
- B cells will also be further analysed by sequencing the IGH-IGL genes, by mass spectrometry analysis of produced antibodies and/or by gene expression. Immunoglobulin genes of interest will be cloned and expressed recombinantly for further analysis, including detailed mapping of epitopes and/or analysis of function in the antibody assays as previously mentioned. Stored B and T cells may also be used in co-culture experiments with other immune cells, to investigate the effects of antigen-specific memory in response to Bp.

T cell responses to Bp will be analysed to determine the quantity, quality and persistence of antigen-specific T cell responses in whole blood, allowing a comparison of cellular immunity in different groups of participants. We will perform a number of analyses.

- For the core T cell assay, whole blood will be in vitro stimulated with antigens and the T cell response will then be analysed by flow cytometry and eventually by measuring cytokine concentrations in the supernatants.
- Innovative single cell-based assays will be developed that can simultaneously survey up to 40 features on single T cells, to explore novel markers of distinct Bp-

antigen specific T cell subtypes, differentiation, memory and tissue residence. We will evaluate: a) in vitro restimulation conditions; b) panels of reagents for analysis of: i) T cell subsets (CD3, CD4 and CD8), ii) function, iii) memory and homing, iv) fitness; and c) use of EuroFlow and mass cytometry (CyTOF) platforms. Single cell gene expression profiling and in-depth RNA-seq, T cell repertoire to various antigens and epigenetic analysis of sorted T cell subsets, will provide information on T cell commitment, allowing a deeper understanding of the global markers and transcriptome of protective Bp-specific T cells.

Dynamics of the immune response to pertussis vaccination

Blood contains a number of different (migrating) immune cell types, which reflect perturbations induced by e.g. systemic vaccination or local infection. Previous vaccination studies have shown that distinct gene expression and functional profiles in peripheral blood, taken soon after vaccination can predict the functionality, magnitude and persistence of the memory response.

In a selected number of samples, we will use RNA-sequencing (RNA-seq) to identify the key signalling pathways that are induced upon aP booster vaccination. At baseline and at different time points shortly after vaccination, whole blood and/or single immune cells will be isolated and stored in RNA-stabilising agents, or used in functional assays. RNA will be isolated and analysed by RNA-seq. Reads will be mapped to the reference genomes and dynamic changes in gene(s) transcription patterns from baseline, will be identified.

In parallel to RNAseq, we will phenotype innate immune cells at the single cell level in peripheral blood, using mass cytometry (CyTOF) and EuroFlow. Measurement of the kinetics of multiple (>60) blood leukocyte subsets at consecutive time points will contribute to the understanding of how different subsets of B and T cells (and their memory and effector functions), as well as innate cells are related to the building of anti-Bp immune responses. Finally, we will examine differences in the major metabolic pathways involved in immune function, including glycolysis, the TCA cycle, etc.

8.4 Withdrawal of individual participants

Participants can leave the study at any time for any reason if they wish to do so, without consequences. The investigator can decide to withdraw a participant from the study for urgent medical reasons, if vaccination status can’t be confirmed (cohort A, B and C) or in case of a failed blood drawing at T0, T1, T2, T3 or T4. In case of a failed blood draw, the appointment can be rescheduled within time window as described in section 3.1 .

8.5 Replacement of individual participants after withdrawal

Participants withdrawn before T4 may be replaced as long as the site is still actively recruiting. Replacement can take place until there are 108 children and 75 adults (36 and 25 per study site) included with all blood samples up to T4 successfully collected.

8.6 Follow-up of participants withdrawn from treatment

Not applicable.

8.7 Premature termination of the study

Boostrix®-IPV is authorised in all the participating countries i.e. Finland, the Netherlands and the U K. The product is routinely used in several other EU countries in the same age groups and considered safe [53]. It is therefore unlikely that serious side effects will occur that can lead to premature termination of the study.

Nevertheless, the sponsor is entitled to terminate the study prematurely if this is beneficial to the health or welfare of the participants. The medical research ethics committee (MREC) will be informed about such a decision.

In case of premature study termination, there will be no consequences for a participant other than that further blood sampling is suspended.

9. SAFETY REPORTING

9.1 Premature termination or suspension of a trial

In accordance to sections 5.16 and 5.21 of the international committee on harmonisation (ICH) guidance on good clinical practice (GCP), the sponsor will suspend the study if there are sufficient grounds to believe that continuation of the study will jeopardise participants' health or safety. The sponsor will notify the accredited MREC without undue delay of a temporary halt including the reason for such an action. The study will be suspended pending a further positive decision by the accredited MREC. The investigator will take care that all participants are kept informed.

9.2 Adverse events, serious adverse events and suspected unexpected serious adverse reactions

9.2.1 Adverse events

AEs are defined as any undesirable experience occurring to a participant during the study, whether or not considered related to the vaccine, the vaccination or the venepuncture. All AEs reported spontaneously by the participant or observed by the investigator or his staff will be recorded and reported to the responsible authority AEs will not be reported to the ethics committee.

9.2.2 Serious adverse events

A SAE is any untoward medical occurrence or affect that at any dose:
-results in death;
-is life threatening (at the time of the event);
-requires hospitalisation or prolongation of existing inpatients' hospitalisation;
-results in persistent or significant disability or incapacity;
-is a congenital anomaly or birth defect; or
-any other important medical event that did not result in any of the outcomes listed above due to medical or surgical intervention but could have been, based upon appropriate judgement by the investigator.
A hospital admission for an elective procedure will not be considered as a SAE.

Procedure in the Netherlands

The investigator(s) will report all SAEs that occur during the study to the sponsor within 24 hours after obtaining knowledge of the events. The sponsor will perform an initial check of the report, request any additional information and ensure it is followed up to completion. All SAE information must be recorded on an SAE form and faxed or

e-mailed to the sponsor. Additional information received for a case (follow-up or corrections to the original case) need to be detailed on a new SAE form and faxed or e-mailed to the sponsor.
The sponsor will report all IMP related SAE's through the web portal ToetsingOnline within 15 days after the sponsor has first knowledge of the serious adverse reactions. SAEs that result in death or are life threatening should be reported expedited. The expedited reporting will occur not later than 7 days after the responsible investigator has first knowledge of the adverse reaction. This is for a preliminary report with another 8 days for completion of the report.
Unrelated SAE's and SAE's related to blood draw will be reported once a year to the MERC
All SAEs with a suspected (possible, probable or definite) relationship to the vaccine (as indicated by the responsible investigator) will be reported by the investigator to Lareb, the national authority for side effects at the end of the study.

Procedure in the UK

All SAEs occurring from taking informed consent until the end of the study will be reported. All SAEs must be reported on the SAE reporting form to the clinical trials and research governance (CTRG) within 24 hours of the Site Study Team becoming aware of the event. The CTRG will perform an initial check of the report, request any additional information, and ensure it is reviewed by the Medical Monitor on a weekly basis. It will also be reviewed at the next Trial Safety Group meeting. All SAE information must be recorded on an SAE form and faxed, or scanned and emailed, to the CTRG. Additional and further requested information (follow-up or corrections to the original case) will be detailed on a new SAE Report Form and faxed/emailed to the CTRG.

Procedure in Finland

The investigator(s) will report all SAEs to the sponsor without undue delay after obtaining knowledge of the events. All SAEs occurring during the study period will be reported within 7 days of first knowledge for SAEs that result in death or are life threatening followed by a period to complete the initial preliminary report.
All SAEs with a suspected (possible, probable or definite) relationship to the vaccine (as indicated by the responsible investigator) will be reported by the investigator to the National Institute for Health and Welfare, Helsinki, Finland, the national authority for side effects.

9.2.3 Suspected unexpected serious adverse reactions

ARs are all untoward and unintended responses to an investigational product related to any dose administered.

Unexpected ARs are suspected unexpected serious adverse reactions (SUSARs) if the following three conditions are met:

- the event must be serious (see chapter 9.2.2);
- there must be a certain degree of probability that the event is a harmful and an undesirable reaction to the medicinal product under investigation, regardless of the administered dose;
- the AR must be unexpected, that is to say, the nature and severity of the AR are not in agreement with the product information as recorded in SmPC for an authorised medicinal product.

Procedure in the Netherlands

The sponsor will report expedite the following SUSARs through the web portal *ToetsingOnline* to the MREC:

- SUSARs that have arisen in the clinical trial that was assessed by the MREC;
- SUSARs that have arisen in other clinical trials of the same sponsor and with the same medicinal product, and that could have consequences for the safety of the participants involved in the clinical trial that was assessed by the MREC.

The remaining SUSARs are recorded in an overview list (line-listing) that will be submitted once every half year to the MREC. This line-listing provides an overview of all SUSARs from the study medicine, accompanied by a brief report highlighting the main points of concern.

The expedited reporting of SUSARs through the web portal *ToetsingOnline* is sufficient as notification to the competent authority.

The sponsor will report expedite all SUSARs to the competent authorities in other Member States, i.e. MHRA in the UK and Fimea in Finland, according to the requirements of the Member States.

The expedited reporting will occur within 15 days after the sponsor has first knowledge of the ARs. For fatal or life threatening cases the term will be maximal 7 days for a preliminary report with another 8 days for completion of the report.

Procedure in the UK

All SUSARs will be reported by the principal investigator to the relevant competent authority and the MREC. Fatal and life-threatening SUSARS will be reported no later than 7 calendar days after the Sponsor or delegate is first aware of the reaction. Any additional relevant information will be reported within 8 calendar days of the initial report. All other SUSARs will be reported within 15 calendar days.

Procedure in Finland

All SUSARs will be reported to the National Institute for Health and Welfare, Helsinki, Finland. An electronic reporting system will be used and reports will be delivered within 7 days of identification of a possible SUSAR.

9.3 Annual safety report

In addition to the expedited reporting of SUSARs, the sponsor will submit, once a year throughout the clinical trial, a list of all suspected (unexpected or expected) SARs to the accredited MREC competent authority, and competent authorities of the concerned member states.

9.4 Follow-up of adverse events

All AEs will be followed until they have abated, or until a stable situation has been reached. Depending on the event, follow up may require additional tests or medical procedures as indicated, and/or referral to the general physician or a medical specialist. SAEs need to be reported until end of study, as defined in the protocol.

9.5 Data safety monitoring committee

For neonatal/maternal clinical trial also performed by the PERISCOPE consortium, a DSMC is installed. The aims of the committee are to safeguard the interests of the trial participants and monitor the safety outcomes of the trials including SAEs. The DMSC for the maternal-neonatal studies is fully independent and will also act in the same composition as a safety committee for the Booster study by reviewing the safety data.

10. STATISTICAL ANALYSIS

As described in section 4.5, 366 participants will be enrolled in the three countries in total, per child cohort in each country 36 participants and per adult cohort 25 participants. A sample size of 105 per child cohort provides 80% power to detect a standardised difference in log-anti-PT IgG at one month post booster of 0.42 between age cohorts allowing for 15% loss to follow up/sample loss. The data obtained from the participants in each country will be combined and analysed per age cohort.

The statistical analysis will be performed on the ITT cohorts of the children. We will endeavour to obtain full follow-up on every participant to allow full ITT analysis, but we will experience the problem of missing data due to withdrawal and loss to follow-up. The results from the study will be presented in comparative summary statistics (difference in means) with 95% confidence intervals (CIs). All the tests will be done at a 5% two-sided significance level. The study results will be reported in accordance with the CONSORT (consolidated standards of reporting trials) 2010 statements. A full detailed analysis plan will be prepared and finalised by an independent statistician within the PERISCOPE consortium.

A per-protocol analysis will also be carried out, allowing the comparison between participants and cohorts who completed the study.

An exploratory analysis will be carried out on the paediatric cohorts and the smaller adult cohorts that will focus on novel and exploratory assays using descriptive statistics.

10.1 Primary study parameter(s)

10.1.1 Serum specific pertussis toxin IgG antibodies

The primary outcome of the booster vaccination studies is the PT specific antibody level at T4 (28 days post booster). Results will be expressed as geometric mean concentrations with confidence intervals (95%-CI) and as reverse cumulative distribution curves. To compare the results between pre- and post-booster vaccination a paired t-test will be used, and in addition, a non-parametric test (Mann-Whitney) to assess the robustness of the results. Also, the cumulative antibody distributions could be compared using the Kolmogorov-Smirnov test. A p-value ≤ 0.05 is considered statistically significant. Results from the different age cohorts in the three countries can be compiled due to the similar vaccination background. For the age cohort B of 11-15 year old children, the vaccination background will consist of either a completely wP or completely aP priming in the first year of life. For the age cohort D participants might not have been vaccinated at all. It is the intention to enrol comparable numbers

of participants with different vaccination background in both cohort B and D, which would allow comparing the effects of an aP booster vaccination in aP versus wP primed children for cohort B and vaccinations versus no vaccinations for cohort D.
Results of the three countries can also be analysed separately per country but the sample size will probably be too small to obtain a normal distribution of antibody concentrations.

10.2 Interim analysis

Not applicable.

10.3 Comparative analyses

The Booster study is part of the PERISCOPE project in which also number of other pertussis studies will be performed, in humans and in baboons. In the comparative analyses, we will capitalise on those parallel studies by determining consistent trends among the parameters: do we observe the same correlations between parameters of protection (e.g. antibody levels) on the one hand and RNA and cell concentrations on the other hand in Bert study as in the other vaccination studies. This will provide support for the detection of system-independent biomarkers of protection that we aim to identify from the PERISCOPE human challenge model data and from the comparison of aP and wP vaccinated children. We will specifically capitalise on the availability of immunological parameters at T5 to determine which transcriptional modules measured at T1 show the highest correlation with them, allowing us to detect short-term biomarkers of long-term protection. We will do those analyses using gene set enrichment analysis with a false discovery rate cut-off of 0.01, using the transcriptional modules [54]. Furthermore, we will compare the primary, secondary, and exploratory readouts from the aP vaccination in the Bert study with those readouts from the aP vaccination in infants and the aP vaccination in infant baboons to determine to what extent the reaction to the aP booster is not only quantitatively but also qualitatively different from primary vaccinations. Comparative analyses will be coordinated by xxx from the Radboud University Medical Centre, a co-investigator on the PERISCOPE application.

10.4 Data management

Data will be managed using OpenClinica. OpenClinica is clinical trial software for electronic data capture (EDC) and clinical data management (CDM), which enables compliance, with regulatory guidelines such as 21 CFR Part 11. This system will allow the managment of the data specific for the BERT study, and will be hosted in the UK site.

Anonymised, processed data of the core, pre-core and exploratory assays that will be performed on the samples from the participants, will also be made available to the PERISCOPE consortium members by the consortium's data management team. PERISCOPE for this propose will use the tranSMART database system.
Using the system will enforce universal annotation of the data to allow sharing, comparison and dissemination. The data in tranSMART system will also contain anonymised patient IDs, including relevant clinical information imported from the CRFs that are entered in OpenClinica (e.g. gender, BMI and date of birth). Via the tranSMART system, those data will remain available after the conclusion of the PERISCOPE project. Universal annotation of the data allows the comparison of samples in assays undertaken in the laboratories of the consortium and the integrative analysis of data from a range of studies using similar laboratory assays across the consortium. The data management team is based at Radboud University Medical Centre, Geert Grooteplein-Zuid 10, 6525 GA Nijmegen Netherlands. It is under the direction of xxx, a co-investigator on the current application.

11. ETHICAL CONSIDERATIONS

11.1 Regulation statement

This clinical study will be performed according to the current rules for good clinical practice (GCP), as described by the committee for proprietary medical products (CPMP) of the EU and the ICH in "Note for guidance on GCP", document CPMP/ICH/135/95", effective since January 17th 1997; and guideline clinical investigation of medicinal products in the paediatric population, document CPMP/ICH/2711/99, effective since July 2000. This is also in accordance to the rules of the Dutch medical research involving human subjects act (WMO), under the general ruling of the clinical trial directive of the EU (2001/20/EU). These rules include the ethical guidelines described in the "Declaration of Helsinki" by the World Medical Association (WMA): 'ethical principles for medical research involving human subjects', adopted by the 18th WMA general assembly, Helsinki, Finland, June 1964; and last amended by the 64th WMA general assembly, Fortaleza, Brazil, October 2013.

11.2 Recruitment and consent

In the Netherlands, home addresses of eligible participants for cohort B, C and D will be attained via the Municipal Administration (gemeentelijke basis administratie in the Netherlands). Eligible participants for cohort A will be approached through the Dutch NIP (dienst vaccinvoorziening en preventieprogramma's (DVP)). The attained addresses will be used only once for the mailing of the folder. The mailing will be taken care of via a mail-order company and after that, the address list will be destroyed. Invitation letters will be sent to the potential participants. This invitation letter includes brief information about the study and a reply card. On the reply card, information about home address, telephone number, email address and preferred contact moment is asked. The reply card can be sent by post to the investigator. After receiving the reply card, the investigator will contact the potential participant, or if applicable his or her parents or legal guardians, to give more information and to check whether the potential participant is eligible for inclusion in the study based on the inclusion and exclusion criteria. Afterwards, an age appropriate extensive PIF together with an informed consent form (ICF) will be sent to the potential participant. Approximately one week after sending the information, the investigator will contact the potential participant a second time to answer additional questions. If the potential participant is still willing to participate, an appointment will be made for the first and second visit at home or at a study site close to where the participant lives. During the first visit (T0), the investigator and the participant will sign the ICF, prior to the first blood

draw and vaccination. Potential participants may also be identified by the study website, and distribution of the study information sheets.

In the UK, potential participants will be approached by mailing out information sheets to age appropriate children via the child health computer department (CHCD), Open Exeter or any other National Health Service equivalent database. The potential study participants will receive the full study information with reply slip inviting them to take part in the study. This information is sent directly from the CHCD. Research staff will not have access to the contact details of potential participants, until the parents themselves provide that information to the study team. If the reply slip is not received within 2 weeks, a study postcard will be sent as a reminder of the study and the information previously sent. Potential participants may also be identified by website-based advertising, poster advertisements, distribution of the study information sheets, GP practices and social media, all of which have been submitted as separate documents to the various governance approval processes. For the paediatric population, it is anticipated that information sessions about the study will be held in schools. The poster, website/social media advertisements and information sheets will display the contact details of the research site and website via which potential participants may indicate their interest in participating in the study. Either potential participants will contact the research team directly, having seen a poster or information sheet; or by email, website, telephone, or by postal reply slips. Participants, parents/guardians interested in participating in the study will be encouraged to complete an online form available at the OVG website, with study information and eligibility criteria, in order to allow possible exclusion of participants based on the inclusion/exclusion criteria, before a formal and detailed contact from the study team. Study staff will contact all participants and families that express an interest in participating in the study: 1) after filling out the online form and considered eligible, or 2) if requested by the participant/family for clarification of the study design and study eligibility criteria before point 1. The study team would contact the families via telephone to provide further details about the study.

In Finland, the potential participants will be informed in local schools in Turku after permission is received from the school authorities. Older participants will be recruited by information in the newspapers and Turku University and Turku University Hospital web pages. The basic contacts and information of the possible participants will be done as in the Netherlands. Those who are interested in participating are invited to contact the study clinic where they will receive a more detailed information package of the design of the study. Those who are willing to participate are asked to contact the study clinic.

Informed consent for minor participants

The Netherlands: for participants under 16, both parents/legal representatives have to sign a parent-legal guardian ICF, unless the child only has one legal parent/representative, e.g. one of the parents is deceased, the father didn't acknowledged the child or the mother is consciously single-parent. In that case, only that legal parent/representative has to sign. In addition, if the minor participant is 12 years or older, he/she will have to sign a separate ICF as well. Children between 11 and 12 years old in cohort B will be asked to sign a separate ICF as well.

UK: for participants under 16, one parent needs to sign, if the minor participant is 10 years or older, he/she will have to co-sign the ICF.

Finland: for participants under 16 one parent or legal guardian needs to sign the ICF The other parent can co-sign if they wish. Written separate consent of the child is required from children 10 years or above.

Personal data

Personal (identity) data are defined as the set of data that together might disclose the identity of a participant: name, date of birth, and full address. The investigator will guarantee that personal data from participants are treated anonymously, unless medical conditions of the participants require release of anonymity.

Study data

Study data are defined as the set of all participant-related variables such as, vaccination status, age, health, data on possible AEs, sex and laboratory data. The storage of data is such that third parties have no access to the data.

Direct access will be granted to authorised representatives from the sponsor or host institution for monitoring and/or audit of the study to ensure compliance with regulations. Anonymised data will be made available to the PERISCOPE consortium members through the consortium's data management team. This is in order to allow the use of samples in assays undertaken in the laboratories of the consortium and to facilitate the integrate analysis of data from a range of studies using similar laboratory assays across the consortium. This team is based at Radboud university medical centre, Geert Grooteplein-Zuid 10, 6525 GA Nijmegen, Netherlands. This data management team at Radboud university Nijmegen are under the direction of xxx.

11.3 Objection by minors or incapacitated participants

Minor participants

The Code of Conduct for minors will be respected.

11.4 Benefits and risks assessment, group relatedness

Participants will benefit from participating in this study by receiving an additional dTap vaccination, which will provide increased protection against pertussis, diphtheria, tetanus and polio. This vaccination is currently not routinely administered to these age groups. From the public health perspective, participation in this study will provide insight into the immune response to a pertussis booster dose in different age groups with different priming backgrounds, as well as in different epidemiological backgrounds. This is expected to contribute to the improvement of the NIP. For this reason, participation of the younger age groups is highly relevant.

Vaccination might be slightly painful or uncomfortable at the time of injection. Boostrix®-IPV is an authorised vaccine. ARs to the vaccine may occur, but are expected to be mild, mainly local and transient. Severe allergic reactions to one of the vaccine components are unlikely to occur; the chance of such an event to occur will reasonably not be larger than found after injection of other vaccines.

Furthermore, slight local discomfort may occur because of the venepuncture. Nonetheless, this is a generally accepted procedure with minimal risk.

11.5 Compensation for injury

RIVM site

Liability insurance as laid down in article 7, subsection 9 of the WMO, is not applicable for the RIVM since the RIVM is a governmental institute and its staff is therefore covered by the institution's liability.

By law, the RIVM is exempted from the compulsory participants' insurance as laid down in article 7, subsection 10 of the WMO. Participants can claim damage resulting from the clinical trial directly at the RIVM. The participants are informed of this arrangement in the Participant Information Form. The coverage for damage to research participants through injury or death caused by the study is listed below:

1. € 650,000 (i.e. six hundred and fifty thousand euros) for death or injury, for each participant who participates in the Research;
2. € 5,000,000 (i.e. five million euros) for death or injury, for all participants who participate in the Research;

3. € 7,500,000 (i.e. seven million five hundred thousand euros) for the total damage incurred by the organisation, for all damage disclosed by scientific research for the Sponsor as 'verrichter' in the meaning of said Act, in each year of insurance coverage. The coverage applies to the damage that becomes apparent during the study or within 4 years after the end of the study.

Oxford Site

The University has a specialist insurance policy in place which would operate in the event of any participant suffering harm as a result of their involvement in the research (Newline Underwriting Management Ltd, at Lloyd's of London, policy numbered: WD1200463).

Turku site

The University Hospital has a specialist insurance policy in place, which would operate in the event of any participant suffering harm because of their involvement in the research.

11.6 Incentives

In the Netherlands, as a compensation for the vaccination and the venepunctures, all participants will receive a total of €45 (children) or €55 (adults) in vouchers after completion of the study. Participants will receive an initial voucher of €30 (children, cohorts A and B) or €40 (adults, cohorts C and D), at T4, 28 days post-vaccination) and an additional voucher of €15 (all cohorts) after the final blood sampling (T5, one year post-vaccination). The difference in compensation between the two paediatric cohorts A and B and the two adult cohorts C and D, reflects the difference in burden for sampling intensity.

In UK, as a compensation for the vaccination and the venepunctures, all adult participants (cohorts C and D) will receive £45 per visit, in a total of £225 for all study visits. The paediatric cohorts (A and B) will not receive any reimbursement.

In Finland, the same procedure will be followed as in the Netherlands.

12. ADMINISTRATIVE ASPECTS, MONITORING AND PUBLICATION

12.1 Handling and storage of data and documents

In this study, the case report form (CRF) entries will be considered source data, as these will be at the site of the original recording for all assessments and measurements made during visits. All study data will be registered in a collection of files (the source documents) and handled confidentially. The information will be entered into a web based electronic CRF (eCRF, OpenClinica™ database stored on a secure University of Oxford server), hosted by University of Oxford – Oxford Vaccine Group . OpenClinica is clinical trial software for electronic data capture (EDC) and clinical data management (CDM), which enables compliance, with regulatory guidelines such as 21 CFR Part 11. The same online database system will be also used across sites. Personal identifiers will not be recorded on study materials. A unique participant number will be used for labelling study materials and for identifying recorded study material. The local investigator or a designated representative will keep the key to the code. The handling of personal data will comply with the personal data protection act for the participating countries (Wet bescherming persoonsgegevens in the Netherlands). All protocol required information collected during the study will be recorded by the investigator or a designated representative. Study findings stored on a computer will be stored in accordance with local data protection laws. The study materials are the property of the consortium as described in the consortium agreement.

Essential documents, according to GCP, source documents will be retained at the study site for a period of 10 years after the last participant has completed the study.

The investigator grants permission to personnel from the sponsor, its representatives and appropriate regulatory authorities for on-site trial-related monitoring, as well as on-site audits, EC review and regulatory inspection(s), providing direct access to source data/documents.

A pseudonymised dataset on each participant will also be entered on a secure electronic database designed and managed by the PERISCOPE consortium. This will include participant's gender, age at inclusion, ethnicity, name of study, vaccination dates and sample dates.

12.2 Handling and storage of samples

To ensure uniform handling and processing of samples the ones that can be frozen without loss of quality will be stored in the PERISCOPE biobank, which is located at the Radboud University Medical Centre, Nijmegen, the Netherlands. This includes storage for short periods of samples that will be analysed at other labs than where the samples were

obtained and material left over after analyses are done. The labs where the analyses will be done during the PERISCOPE project are all part of the PERISCOPE consortium. The PERISCOPE biobank also includes storage of left over samples for maximally 10 years after the end of the PERISCOPE project. Any samples from the Biobank will only be used for the study purposes and objectives of the PERISCOPE project. This includes samples that are left after the duration of the project, as we anticipate analysis of samples will continue after the project has finished. The types of analyses that will be done on the samples during the project are described in the research proposal. Samples that are left over after the project will only be used to answer the research questions of the PERISCOPE project. To ensure this, we will install a group of senior PI's of PERISCOPE to oversee and decide on the use of the samples after PERISCOPE has ended.

The Radboud university medical center is listed in the Commercial Register of the Chamber of Commerce under file number 41055629.

12.3 Monitoring and Quality Assurance

A representative of the sponsor may monitor this study throughout various stages of the trial. A monitoring plan (risk based) detailing the monitoring activities will be finalised by the sponsor before the study is initiated and may include (but not be limited to) the following activities:

- presence of written approval by MREC;
- adherence to protocol and GCP;
- facilities and procedures at the study site;
- presence of personally signed and dated ICFs;
- appropriate training and authorisation of study personnel;
- accuracy and completeness of trial data;
- presence of an up-to-date trial site file.

For the purpose of compliance with GCP, it may be necessary to conduct a site audit performed by authorised representatives of the sponsor and/or a Regulatory Authority and/or the Ethics Committee. This may occur at any time from start to after conclusion of the study.

12.4 Amendments

A 'substantial amendment' is defined as an amendment to the terms of the MREC application, or to the protocol or any other supporting documentation, that is likely to affect to a significant degree:

- the safety or physical or mental integrity of the participants of the trial;

- the scientific value of the trial;
- the conduct or management of the trial; or
- the quality or safety of any intervention used in the trial.

All substantial amendments will be notified to the MREC and to the competent authority. Non-substantial amendments will not be notified to the accredited MREC and the competent authority, but will be recorded and filed by the sponsor.

12.5 Annual progress report

The sponsor/investigator will submit a summary of the progress of the trial to the accredited MREC once a year. Information will be provided on the date of inclusion of the first participant, numbers of participants included and numbers of participants that have completed the trial, SAEs/ SARs, other problems, and amendments.

12.6 End of study report

The investigator will notify the accredited MREC of the end of the study within a period of 90 days. The end of the study is defined as the last participant's last visit.

12.7 Public disclosure and publication policy

The study results will be reported in an internal report and submitted for publication in peer-reviewed journals. The steering committee of the PERISCOPE consortium will develop a detailed publication policy to ensure proper and fair representation and conduct according to Vancouver international guidelines for publication. The investigators of the three participating countries will jointly write the clinical manuscript(s) for publication. For the Bert study in this protocol, the Netherlands will take the lead in making a first draft of the principal clinical paper. The Investigators will all be involved in reviewing additional drafts of additional manuscripts, abstracts, press releases and other publications arising from the study. Authors will acknowledge that the study was funded by the Innovative Medicines Initiative (European Union funding for research and innovation) and by the Bill and Melinda Gates foundation. Authorship will be determined in accordance with the ICMJE guidelines and other contributors will be acknowledged. Prior to the start of the study, the study will be registered in a public clinical trial registry.

13. STRUCTURED RISK ANALYSIS

13.1 Potential issues of concern

Not applicable.

13.2 Synthesis

Section 13.1 is not applicable because Boostrix®-IPV is an authorised vaccine in the participating countries and used within the indication.

14. REFERENCES

1. Cherry JD: **Comparative efficacy of acellular pertussis vaccines: an analysis of recent trials**. *Pediatr Infect Dis J* 1997, **16**(4 Suppl):S90-96.
2. Aoyama T, Murase Y, Gonda T, Iwata T: **Type-specific efficacy of acellular pertussis vaccine**. *American journal of diseases of children (1960)* 1988, **142**(1):40-42.
3. Podda A, De Luca EC, Contu B, Furlan R, Maida A, Moiraghi A, Stramare D, Titone L, Uxa F, Di Pisa F *et al*: **Comparative study of a whole-cell pertussis vaccine and a recombinant acellular pertussis vaccine. The Italian Multicenter Group for the Study of Recombinant Acellular Pertussis Vaccine**. *The Journal of pediatrics* 1994, **124**(6):921-926.
4. Schmitt HJ, Schuind A, Knuf M, Zepp F, Beutel K, Wirsing von Konig CH, Neiss A, Bock HL, Bogaerts H, Clemens R: **Acellular pertussis vaccines: the rationale for an efficacy trial in Germany**. *The Journal of infectious diseases* 1996, **174 Suppl 3**:S287-290.
5. **Placebo-controlled trial of two acellular pertussis vaccines in Sweden--protective efficacy and adverse events. Ad Hoc Group for the Study of Pertussis Vaccines**. *Lancet* 1988, **1**(8592):955-960.
6. de Melker HE, Conyn-van Spaendonck MA, Rümke HC, van Wijngaarden JK, Mooi FR, Schellekens JF: **Pertussis in The Netherlands: an outbreak despite high levels of immunization with whole-cell vaccine**. *Emerg Infect Dis* 1997, **3**(2):175-178.
7. Mooi FR, van Loo IH, van Gent M, He Q, Bart MJ, Heuvelman KJ, de Greeff SC, Diavatopoulos D, Teunis P, Nagelkerke N *et al*: **Bordetella pertussis strains with increased toxin production associated with pertussis resurgence**. *Emerging infectious diseases* 2009, **15**(8):1206-1213.
8. van Hoek A, Campbell H, Amirthalingam G, Andrews N, Miller E: **The number of deaths among infants under one year of age in England with pertussis: results of a capture/recapture analysis for the period 2001 to 2011**. *Euro surveillance : bulletin europeen sur les maladies transmissibles = European communicable disease bulletin* 2013, **18**(9).
9. Elomaa A, He Q, Minh NN, Mertsola J: **Pertussis before and after the introduction of acellular pertussis vaccines in Finland**. *Vaccine* 2009, **27**(40):5443-5449.
10. Roehr B: **Whooping cough outbreak hits several US states**. *BMJ* 2010, **341**:c4627.
11. Halperin BA, Halperin SA: **The reemergence of pertussis and infant deaths: is it time to immunize pregnant women?** *Future microbiology* 2011, **6**(4):367-369.
12. Octavia S, Sintchenko V, Gilbert GL, Lawrence A, Keil AD, Hogg G, Lan R: **Newly emerging clones of Bordetella pertussis carrying prn2 and ptxP3 alleles implicated**

in Australian pertussis epidemic in 2008-2010. *The Journal of infectious diseases* 2012, **205**(8):1220-1224.

13. Kamano H, Mori T, Maeta H, Taminato T, Ishida T, Kishimoto N, Katami T, Sato M, Kamachi K, Mochida Y: **Analysis of Bordetella pertussis agglutinin titers during an outbreak of pertussis at a university in Japan**. *Japanese journal of infectious diseases* 2010, **63**(2):108-112.
14. de Greeff SC, Mooi FR, Schellekens JF, de Melker HE: **Impact of acellular pertussis preschool booster vaccination on disease burden of pertussis in The Netherlands**. *Pediatr Infect Dis J* 2008, **27**(3):218-223.
15. Berbers GA, de Greeff SC, Mooi FR: **Improving pertussis vaccination**. *Human vaccines* 2009, **5**(7):497-503.
16. Billingsley M: **Pregnant women in UK are offered whooping cough vaccine to protect newborns**. *BMJ (Clinical research ed)* 2012, **345**:e6594.
17. England HPA-PH: **Laboratory confirmed cases of pertussis reported to the enhanced pertussis surveillance programme in 2012**. *Health Protection Report* 2013, **7**(14-17):26-28.
18. Berbers GAM, Lafeber AB, Labadie J, Vermeer-de Bondt PE, Bolscher DJA, Plantinga AD: **A randomised controlled study with whole-cell or acellular pertussis vaccines in combination with regular DT-IPV vaccine and a new poliomyelitis (IPV-Vero) component in children 4 years of age in the Netherlands**. *RIVM report 105000 001* 1999:1-67.
19. van der Maas NAT, Mooi FR, Berbers GAM, Swaan C, de Greeff SC, de Melker HE: **Kinkhoest, terug van weggeweest**. *Infectieziekten Bulletin - RIVM* 2012, **23**(8):229-234.
20. Amirthalingam G, Gupta S, Campbell H: **Pertussis immunisation and control in England and Wales, 1957 to 2012: a historical review**. *Euro surveillance : bulletin Europeen sur les maladies transmissibles = European communicable disease bulletin* 2013, **18**(38).
21. McVernon J, Andrews N, Slack MP, Ramsay ME: **Risk of vaccine failure after Haemophilus influenzae type b (Hib) combination vaccines with acellular pertussis**. *Lancet (London, England)* 2003, **361**(9368):1521-1523.
22. Southern J, McVernon J, Gelb D, Andrews N, Morris R, Crowley-Luke A, Goldblatt D, Miller E: **Immunogenicity of a fourth dose of Haemophilus influenzae type b (Hib) conjugate vaccine and antibody persistence in young children from the United Kingdom who were primed with acellular or whole-cell pertussis component-containing Hib combinations in infancy**. *Clinical and vaccine immunology : CVI* 2007, **14**(10):1328-1333.

23. Campbell H, Amirthalingam G, Andrews N, Fry NK, George RC, Harrison TG, Miller E: **Accelerating control of pertussis in England and Wales**. *Emerg Infect Dis* 2012, **18**(1):38-47.

24. England PH: **Pertussis: guidelines for public health management - GOV.UK**. In. Edited by Group PG, July 2016 edn; 2016.

25. England PH: **The complete routine immunisation schedule**. In., 28 June 2016 edn: GOV.UK; 2014.

26. de Melker HE, Versteegh FG, Schellekens JF, Teunis PF, Kretzschmar M: **The incidence of Bordetella pertussis infections estimated in the population from a combination of serological surveys**. *The Journal of infection* 2006, **53**(2):106-113.

27. de Greeff SC, de Melker HE, van Gageldonk PG, Schellekens JF, van der Klis FR, Mollema L, Mooi FR, Berbers GA: **Seroprevalence of pertussis in The Netherlands: evidence for increased circulation of Bordetella pertussis**. *PloS one* 2010, **5**(12):e14183.

28. England PH: **Pertussis: laboratory confirmed cases reported in England 2015 - GOV.UK**. In., vol. 10, 6 May 2016 edn; 2015.

29. Wiese-Posselt M, Tertilt C, Zepp F: **Vaccination Recommendations for Germany**. *Dtsch Arztebl Int* 2011, **108**(45):771-780.

30. Skoff TH, Cohn AC, Clark TA, Messonnier NE, Martin SW: **Early Impact of the US Tdap vaccination program on pertussis trends**. *Archives of pediatrics & adolescent medicine* 2012, **166**(4):344-349.

31. Zepp F, Heininger U, Mertsola J, Bernatowska E, Guiso N, Roord J, Tozzi AE, Van Damme P: **Rationale for pertussis booster vaccination throughout life in Europe**. *The Lancet Infectious diseases* 2011, **11**(7):557-570.

32. Canada PHAo: **Canadian Immunization Guide - Part 4 "Active Vaccines"**. 2012.

33. Jackson DW, Rohani P: **Perplexities of pertussis: recent global epidemiological trends and their potential causes**. *Epidemiology and infection* 2013:1-13.

34. Crowcroft NS, Pebody RG: **Recent developments in pertussis**. *Lancet (London, England)* 2006, **367**(9526):1926-1936.

35. Hendrikx LH, Schure RM, Ozturk K, de Rond LG, de Greeff SC, Sanders EA, Berbers GA, Buisman AM: **Different IgG-subclass distributions after whole-cell and acellular pertussis infant primary vaccinations in healthy and pertussis infected children**. *Vaccine* 2011, **29**(40):6874-6880.

36. Hallander HO, Gustafsson L, Ljungman M, Storsaeter J: **Pertussis antitoxin decay after vaccination with DTPa. Response to a first booster dose 3 1/2-6 1/2 years after the third vaccine dose**. *Vaccine* 2005, **23**(46-47):5359-5364.

37. Riffelmann M, Littmann M, Hulsse C, von Konig CH: **Antibody decay after immunisation of health-care workers with an acellular pertussis vaccine**. *European journal of clinical microbiology & infectious diseases : official publication of the European Society of Clinical Microbiology* 2009, **28**(3):275-279.
38. Cherry JD, Gornbein J, Heininger U, Stehr K: **A search for serologic correlates of immunity to Bordetella pertussis cough illnesses**. *Vaccine* 1998, **16**(20):1901-1906.
39. Storsaeter J, Hallander HO, Gustafsson L, Olin P: **Levels of anti-pertussis antibodies related to protection after household exposure to Bordetella pertussis**. *Vaccine* 1998, **16**(20):1907-1916.
40. Hewlett EL, Halperin SA: **Serological correlates of immunity to Bordetella pertussis**. *Vaccine* 1998, **16**(20):1899-1900.
41. Edelman KJ, He Q, Makinen JP, Haanpera MS, Tran Minh NN, Schuerman L, Wolter J, Mertsola JA: **Pertussis-specific cell-mediated and humoral immunity in adolescents 3 years after booster immunization with acellular pertussis vaccine**. *Clinical infectious diseases : an official publication of the Infectious Diseases Society of America* 2004, **39**(2):179-185.
42. Hendrikx LH, Felderhof MK, Ozturk K, de Rond LG, van Houten MA, Sanders EA, Berbers GA, Buisman AM: **Enhanced memory B-cell immune responses after a second acellular pertussis booster vaccination in children 9 years of age**. *Vaccine* 2011, **30**(1):51-58.
43. Mills KH: **Immunity to Bordetella pertussis**. *Microbes and infection* 2001, **3**(8):655-677.
44. Klein NP, Bartlett J, Rowhani-Rahbar A, Fireman B, Baxter R: **Waning protection after fifth dose of acellular pertussis vaccine in children**. *N Engl J Med* 2012, **367**(11):1012-1019.
45. Warfel JM, Zimmerman LI, Merkel TJ: **Acellular pertussis vaccines protect against disease but fail to prevent infection and transmission in a nonhuman primate model**. *Proceedings of the National Academy of Sciences of the United States of America* 2014, **111**(2):787-792.
46. Le T, Cherry JD, Chang SJ, Knoll MD, Lee ML, Barenkamp S, Bernstein D, Edelman R, Edwards KM, Greenberg D *et al*: **Immune responses and antibody decay after immunization of adolescents and adults with an acellular pertussis vaccine: the APERT Study**. *The Journal of infectious diseases* 2004, **190**(3):535-544.
47. de Greeff SC, de Melker HE, Westerhof A, Schellekens JF, Mooi FR, van Boven M: **Estimation of household transmission rates of pertussis and the effect of cocooning vaccination strategies on infant pertussis**. *Epidemiology (Cambridge, Mass)* 2012, **23**(6):852-860.

48. Hendrikx LH, Ozturk K, de Rond LG, de Greeff SC, Sanders EA, Berbers GA, Buisman AM: **Serum IgA responses against pertussis proteins in infected and Dutch wP or aP vaccinated children: an additional role in pertussis diagnostics**. *PloS one* 2011, **6**(11):e27681.

49. Schure RM, Hendrikx LH, de Rond LG, Ozturk K, Sanders EA, Berbers GA, Buisman AM: **T-cell responses before and after the fifth consecutive acellular pertussis vaccination in 4-year-old Dutch children**. *Clinical and vaccine immunology : CVI* 2012, **19**(11):1879-1886.

50. von Hunolstein C, Aggerbeck H, Andrews N, Berbers G, Fievet-Groyne F, Maple PA, Olander RM, Raux M, Tischer A: **European sero-epidemiology network: standardisation of the results of diphtheria antitoxin assays**. *Vaccine* 2000, **18**(28):3287-3296.

51. de Melker HE, van den Hof S, Berbers GA, Nagelkerke NJ, Rumke HC, Conyn-van Spaendonck MA: **A population-based study on tetanus antitoxin levels in The Netherlands**. *Vaccine* 1999, **18**(1-2):100-108.

52. van Gageldonk PG, van Schaijk FG, van der Klis FR, Berbers GA: **Development and validation of a multiplex immunoassay for the simultaneous determination of serum antibodies to Bordetella pertussis, diphtheria and tetanus**. *Journal of immunological methods* 2008, **335**(1-2):79-89.

53. McCormack PL: **Reduced-antigen, combined diphtheria, tetanus and acellular pertussis vaccine, adsorbed (Boostrix(R)): a review of its properties and use as a single-dose booster immunization**. *Drugs* 2012, **72**(13):1765-1791.

54. Li S, Rouphael N, Duraisingham S, Romero-Steiner S, Presnell S, Davis C, Schmidt DS, Johnson SE, Milton A, Rajam G *et al*: **Molecular signatures of antibody responses derived from a systems biology study of five human vaccines**. *Nature immunology* 2014, **15**(2):195-204.
