# Supplementary material for: Antiviral responses induced by Tdap-IPV vaccination are associated with persistent humoral immunity to Bordetella pertussis
Source: Nat Commun. 2024 Mar 8;15:2133. doi: 10.1038/s41467-024-46560-w (PMC10923912; doi:10.1038/s41467-024-46560-w)
Supplement: Supplementary file 2 — Reporting Summary [file 41467_2024_46560_MOESM2_ESM.pdf]

nature portfolio

Corresponding author(s): Dr. Dimitri Diavatopoulos

Last updated by author(s): Oct 31, 2023

Reporting Summary

Nature Portfolio wishes to improve the reproducibility of the work that we publish. This form provides structure for consistency and transparency in reporting. For further information on Nature Portfolio policies, see our Editorial Policies and the Editorial Policy Checklist.

Statistics

For all statistical analyses, confirm that the following items are present in the figure legend, table legend, main text, or Methods section.

n/a	Confirmed	
☐	☒	The exact sample size (*n*) for each experimental group/condition, given as a discrete number and unit of measurement
☐	☒	A statement on whether measurements were taken from distinct samples or whether the same sample was measured repeatedly
☐	☒	The statistical test(s) used AND whether they are one- or two-sided<br>*Only common tests should be described solely by name; describe more complex techniques in the Methods section.*
☐	☒	A description of all covariates tested
☐	☒	A description of any assumptions or corrections, such as tests of normality and adjustment for multiple comparisons
☐	☒	A full description of the statistical parameters including central tendency (e.g. means) or other basic estimates (e.g. regression coefficient) AND variation (e.g. standard deviation) or associated estimates of uncertainty (e.g. confidence intervals)
☐	☒	For null hypothesis testing, the test statistic (e.g. *F*, *t*, *r*) with confidence intervals, effect sizes, degrees of freedom and *P* value noted<br>*Give P values as exact values whenever suitable.*
☒	☐	For Bayesian analysis, information on the choice of priors and Markov chain Monte Carlo settings
☒	☐	For hierarchical and complex designs, identification of the appropriate level for tests and full reporting of outcomes
☐	☒	Estimates of effect sizes (e.g. Cohen's *d*, Pearson's *r*), indicating how they were calculated

Our web collection on statistics for biologists contains articles on many of the points above.

Software and code

Policy information about availability of computer code

Data collection	collection of flow cytometry data: FACS Aria II cell sorter (BD Bioscience) with FACSDiva version 7, antibody data: Bio-Plex LX200 (Bio-Rad), complete blood counts: Sysmex XN-450 haematology analyser, mass cytometry data: Helios mass cytometer (Fluidigm), Gene expression data: Illumina Next Seq 550.
Data analysis	All statistical analyses were performed using the Rstudio environment (version 3.6.2), with libraries 'stats' (hypothesis tests and correlations), 'lme4' (v1.1-33), 'lmerTest' (v3.1-3) for mixed-effects modeling and associated p-values. R library "Seurat" (v3) was used for single-cell RNAseq analyses; "DESeq2" (v1.38.3) for whole-blood RNA seq analyses. Cytobank v6.1.2 for ex vivo data; Cytobank v7.3.0 for in vitro data (Beckman Coulter) was used for analysis of mass cytometry data.<br><br>Code availability:<br>R code to replicate the findings of this study is available on the corresponding Github repository (https://doi.org/10.5281/zenodo.10560736.)

For manuscripts utilizing custom algorithms or software that are central to the research but not yet described in published literature, software must be made available to editors and reviewers. We strongly encourage code deposition in a community repository (e.g. GitHub). See the Nature Portfolio guidelines for submitting code & software for further information.

Data

Policy information about availability of data

All manuscripts must include a data availability statement. This statement should provide the following information, where applicable:

- Accession codes, unique identifiers, or web links for publicly available datasets
- A description of any restrictions on data availability
- For clinical datasets or third party data, please ensure that the statement adheres to our policy

Data availability:
Whole blood and single-cell RNA sequencing data of this study has been deposited to the GEO database (GSE195627, hyperlink = https://www.ncbi.nlm.nih.gov/geo/query/acc.cgi?acc=GSE195627). The processed data generated in this study are provided in the Source Data file. The raw data are available from the corresponding author upon reasonable request. The raw data are not publicly available due to data privacy laws. External whole blood RNA sequencing data analyzed in this study is also available on GEO (GSE152683, hyperlink = https://www.ncbi.nlm.nih.gov/geo/query/acc.cgi?acc=GSE152683). External antibody data analyzed in this study are available in the original report of the corresponding study (hyperlink = https://insight.jci.org/articles/view/141023).

Research involving human participants, their data, or biological material

Policy information about studies with human participants or human data. See also policy information about sex, gender (identity/presentation), and sexual orientation and race, ethnicity and racism.

Reporting on sex and gender	Sex was not explicitly considered in the study design but all children between ages 11 and 15 years old were eligible for inclusion in the study. Male and female adolescents were included in the present study (NL cohort: N = 14, 8 males and 6 females; UK cohort: N = 12, 6 males and 6 females). Sex was self-reported by study participants. Source data is provided disaggregated by sex. Table S1 contains participant characteristics including sex. Sex-based analyses are provided in Figure S4A and Table S2.
Reporting on race, ethnicity, or other socially relevant groupings	Not applicable, no socially constructed or socially relevant categorization variables were used in this manuscript
Population characteristics	The pertussis vaccine (acellular pertussis, aP or whole-cell pertussis, wP) received during infancy was considered as a relevant covariate (referred to as 'background' in the manuscript) for participants in the NL cohort (aP, N = 7; wP, N = 7). All participants in the UK cohort received aP in infancy. Assignment of aP or wP background in this study was based the participant date of birth and the available pertussis vaccine in the Netherlands at that time. Age was also considered a relevant covariate (NL cohort: range = 12-15 years; UK cohort range = 11 - 13 years). Sex was also considered a relevant covariate (NL cohort: Male, N = 8; Female = 6. UK cohort: Male, N = 6; Female N = 6).
Recruitment	Participants in the Netherlands cohort were recruited by mail-outs in the Hoofddorp region, facilitated by the Municipal Administration, and the study was conducted by the Spaarne Academy (Spaarne Hospital, Hoofddorp, the Netherlands). Participants in the UK cohort were recuirted via mail out to eligible participants within postal areas. Participants received received moderate compensation.
Ethics oversight	This trial was approved by the Medical Research Ethics Committees United (MEC-U, NL60807.100.17-R17.039) in the Netherlands and the South Central - Hampshire B Research Ethics Committee (REC, 19/SC/0368) in the UK.

Note that full information on the approval of the study protocol must also be provided in the manuscript.

Field-specific reporting

Please select the one below that is the best fit for your research. If you are not sure, read the appropriate sections before making your selection.

☒ Life sciences ☐ Behavioural & social sciences ☐ Ecological, evolutionary & environmental sciences

For a reference copy of the document with all sections, see nature.com/documents/nr-reporting-summary-flat.pdf

Life sciences study design

All studies must disclose on these points even when the disclosure is negative.

Sample size	N = 14 participants were included in the Netherlands cohort. N = 12 participants were included in the UK cohort. The number of participants included in the present study was informed on the basis of feasibility for carrying out a study for systems vaccinology.
Data exclusions	No exclusions
Replication	All antibody measurements were independently performed in duplicate, with all replication attempts being successful. All cellular measurements, e.g. mass cytometry and flow cytometry measurements, were performed as a single experiment. Gene expression measurments (whole blood rnaseq and single-cell RNA seq) were performed as a single experiment.

Randomization	Randomization was not applicable to this study since there were not multiple intervention groups (ie, all study participants received Tdap-IPV vaccination).
Blinding	Blinding was not applicable to this study since there were not multiple intervention groups (ie, all study participants received Tdap-IPV vaccination).

Reporting for specific materials, systems and methods

We require information from authors about some types of materials, experimental systems and methods used in many studies. Here, indicate whether each material, system or method listed is relevant to your study. If you are not sure if a list item applies to your research, read the appropriate section before selecting a response.

Materials & experimental systems

n/a	Involved in the study
☐	☒ Antibodies
☒	☐ Eukaryotic cell lines
☒	☐ Palaeontology and archaeology
☒	☐ Animals and other organisms
☐	☒ Clinical data
☒	☐ Dual use research of concern
☒	☐ Plants

Methods

n/a	Involved in the study
☒	☐ ChIP-seq
☐	☒ Flow cytometry
☒	☐ MRI-based neuroimaging

Antibodies

Antibodies used

Flow cytometry antibodies are available in Figure S6 of the manuscript.
Antigen Supplier Fluorochrome Clone Catelog No. Lot Titration (ul)
CD141 BD Biosciences BV421 1A4 565321 6291827 2.5
CD45 Cytognos OC515 GA90 CYT-45OC 520371 10
CD62L Biolegend BV605 DREG-56 304834 B213455 5
HLA-DR BD Biosciences BV711 G46-6 563696 7068615 2.5
CD16 BD Biosciences BV786 3G8 563690 7139586 5
CD1c BD Biosciences BB515 F10/21A3 565054 6083777 5
CD36 Immunostep PerCP Cy5.5 CLB-IVC7 36PP5-100T 590015 10
Slan Miltenyi Biotech PE DD.1 130-093-029 5161227022 10
FCeR1 eBioscience PE AER-37 12-5899-42 4273945 5
CD14 BD Biosciences PE-CF594 MOP9 562335 7174983 5
CD33 BD Biosciences PE-Cy7 P67.6 333952 6279949 5
IREM2 Immunostep APC UP-H2 IREM2A-100T 123541 10
CD303 Miltenyi Biotech APC AC144 130-090-905 5161221268 10
CD56 Biolegend APC-Cy7 HCD56 318332 B233132 5
CD3 BD Biosciences BUV395 SK7 564001 6294654 5
CD19 BD Biosciences BUV395 SJ25C1 563549 6273604 5

Mass cytometry antibodies for analysis of whole blood are provided in table S2:
Antigen Supplier Isotype Clone Reference Lot Titration (ul)
CD45 Fluidigm France 89Y HI30 3089003B 1531706 1
CD8 CHUV/Biolegend 113In RPA-T8 301018 24082017 1
CD4 CHUV/Biolegend 115In RPA-T4 300516 07072017 1
CD19 Fluidigm France 142Nd HIB19 3142001B 1031708 2
HLA-DR Fluidigm France 143Nd L243 3143013B 3421602 2
CD69 Fluidigm France 144Nd FN50 3144018B 0041711 1
CD31 Fluidigm France 145Nd WM59 3145004B 3531201 2
CD86 CHUV/Biolegend 146Nd IT2.2 305435 20022015 1
CD7 Fluidigm France 147Sm CD7-6B7 3147006B 2741608 1
CD16 Fluidigm France 148Nd 3G8 3148004B 0831702 2
IL12p40 CHUV/Biolegend 149Sm C11.5 501813 10072017 0.8
IFNa CHUV/Myltenyi Biotec 150Nd LT27.295 130-092-604 10072017 0.6
CD123 Fluidigm France 151Eu 6H6 3151001B 0521709 2
TNFa Fluidigm France 152Sm Mab11 3152002B 0841602 0.5
IL1b CHUV/Lab Force 153Eu AS10 LS-C26495-500 29052017 0.5
IL6 Fluidigm France 154Sm MQ2-13AS 3154011B 1121511 0.8
CD27 Fluidigm France 155Gd L128 3155001B 1031712 1
TCRg/d CHUV/Biolegend 156Gd B1 331204 17072017 1
CD33 Fluidigm France 158Gd WM53 3158001B 0571502 1
NKp30 Fluidigm France 159Tb Z25 3159017B 1481505 1
CD14 Fluidigm France 160Gd M5E2 3160001B 1351724 2
CD1c CHUV/Biolegend 161Dy L161 331502 06072017 1
CD11c Fluidigm France 162Dy Bu15 3162005B 1561503 2

CD62L CHUV/Biolegend 163Dy DREG-56 304835 17072017 1
IL17a Fluidigm France 164Dy N49-653 3164002B 0161713 0.8
IFNg Fluidigm France 165Ho B27 3165002B 0911301 0.5
NKG2D Fluidigm France 166Er ON72 3169013B 0091703 1
CD38 Fluidigm France 167Er HIT2 3167001B 0191508 1
CD66b CHUV/BD Biosciences 168Er G10F5 555723 29032017 1
NKG2A Fluidigm France 169Tm Z199 3169013B 0961515 1
CD3 Fluidigm France 170Er UCHT1 3170001B 2581608 1
Granzyme B Fluidigm France 171Yb GB11 3171002B 1031504 0.5
CD15 Fluidigm France 172Yb W6D3 3172021B 0791506 1
CD141 Fluidigm France 173Yb 1A4 3173002B 2351601 2
NKG2 Fluidigm France 174Yb HP-3D9 3174015B 2181508 1
Perforin Fluidigm France 175Lu B-D48 3175004B 3421604 0.6
CD56 Fluidigm France 176Yb R19-760 3176013B 1031703 2
CD11b Fluidigm France 209Bi ICRF44 3209003B 2241612 1

Mass cytometry antibodies used for in vitro stimulation experiments are provided in table S5
Antigen Supplier Isotype Clone Reference Lot Titration (ul)
CD8 CHUV/Biolegend 113In RPA-T8 301018 08112018 0.3
CD4 CHUV/Biolegend 115In RPA-T4 300516 11092017 0.8
CD45 Fluidigm France 141Pr HI30 3141009B 3331705 0.7
CD19 Fluidigm France 142Nd HIB19 3142001B 0381907 1.5
HLA-DR Fluidigm France 143Nd L243 3143013B 0941808 0.75
CD69 Fluidigm France 144Nd FN50 3144018B 3041705 0.63
CD31 Fluidigm France 145Nd WM59 3145004B 1351719 0.6
CD86 CHUV/Biolegend 146Nd IT2.2 305410 17072017 0.4
CD7 Fluidigm France 147Sm CD7-6B7 3147006B 2741608 0.56
CD16 Fluidigm France 148Nd 3G8 3148004B 1571806 0.75
CD56 Fluidigm France 149Sm NCAM16.2 3149021B 3331703 0.3
pSTAT5 Fluidigm France 150Nd 47 3150005A 0391801 1
CD123 Fluidigm France 151Eu 6H6 3151001B 2431808 0.8
pAKT Fluidigm France 152Sm D9E 3152005A 1201812 2
pSTAT1 Fluidigm France 153Eu 58D6 3153003A 2001805 2
CD3 Fluidigm France 154Sm UCHT1 3154003B 3391723 0.25
CD27 Fluidigm France 155Gd L128 3155001B 1731805 0.5
pp38 CHUV/Biolegend 156Gd D3F9 3156002A 3441603 1
pSTAT3 Fluidigm France 158Gd 4/P-STAT3 3158005A 1501710 2.6
pMAPKAPK2 Fluidigm France 159Tb 27B7 3159010A 1111705 2
CD14 Fluidigm France 160Gd M5E2 3160001B 0241814 1.2
CD1c CHUV/Biolegend 161Dy L161 331502 17072017 0.6
CD11c Fluidigm France 162Dy Bu15 3162005B 0041909 1.2
CD62L CHUV/Biolegend 163Dy DREG-56 304835 17072017 0.6
CD45RA Fluidigm France 164Dy HI100 555486 16082018 0.5
pNFKb Fluidigm France 165Ho K10-895.12.50 558393 08112018 1.4
NKG2D Fluidigm France 166Er ON72 3169013B 2511702 0.63
CD38 Fluidigm France 167Er HIT2 3167001B 1031705 0.38
CD66b CHUV/BD Biosciences 168Er G10F5 555723 02072018 0.3
NKG2A Fluidigm France 169Tm Z199 3169013B 0961515 0.63
pERK 1/2 Fluidigm France 170Er MILAN8R 14-9109-82 00072019 1
CD20 Fluidigm France 171Yb 2H7 3171012B 0251702 0.5
CX3CR1 Fluidigm France 172Yb 2A9-1 3172017B 1351727 0.5
CD141 Fluidigm France 173Yb 1A4 331204 2351601 0.5
TCRgd CHUV/Biolegend 173Yb B1 3173002B 05092018 0.75
pSTAT4 Fluidigm France 174Yb 38/p-Stat4 3174005A 3271607 2
pSTAT6 Fluidigm France 175Lu 18 3175009A 0641501 2
pCREB Fluidigm France 176Yb 87G3 3176005A 2371710 1
CD11b Fluidigm France 209Bi ICRF44 3209003B 2241612 0.8
CD45 CHUV/Biolegend 194Pt HI30 304002 07122018 0.5
CD45 CHUV/Biolegend 195Pt HI31 304002 11092018 0.5
CD45 CHUV/Biolegend 198Pt HI30 304002 11042019 0.5
CD45 Fluidigm France 89Y HI30 3089003B 3321821 0.6

Secondary antibody for detection of PT-, PRN-, FHA-, TT- and DT- IgG antibodies: R-phycoerythrin conjugated Goat anti-Human IgG (γ chain specific), Jackson Immunoresearch (cat. 109-115-098) , 1:200 dilution.
Coating of beads as part of assay to detect poliovirus antibody in human sera:
antipoliovirus type 1 clone 9B4 (HYB 295-17-02 ThermoFischer scientific, Waltham, MA USA), type 2 clone 24E2 (HYB 294-06-02 ThermoFischer scientific) and type 3 clone 4D5 (HYB 300-06-02 ThermoFischer scientific); each at 50ug/ml

In-house reference sera for quantification of pertussis, TT, and Dt, poliovirus IgG were calibrated against WHO reference sera:
Pertussis (NIBSC code: 06/140)
TT (NIBSC code: TE-3)
Dt (NIBSC code: DI-3)
Poliovirus (NIBSC code: 82/585)

Validation	Specificity of flow cytometry and mass cytometry antibodies is confirmed using flow cytometry/mass cytometry of human peripheral blood cells.<br>Specificity of secondary antibodies for detection of human IgG is confirmed using immunoelectrophoresis and/or ELISA.<br>Specificity of monoclonal poliovirus antibodies is confirmed using ELISA.<br>Relevant references, technical data sheets providing data on the primary application, and certificates of analysis from specific lots is available from the manufacturer.<br>Validation of in house pertussis reference standard is provided in the publication: van Gageldonk et al. 2008 https://doi.org/10.1016/j.jim.2008.02.018.<br>Validation of in house poliovirus reference standards is provided in the publication: Schepp RM et al. 2016 https://doi.org/10.1016/j.jviromet.2016.12.006

Clinical data

Policy information about clinical studies

All manuscripts should comply with the ICMJE guidelines for publication of clinical research and a completed CONSORT checklist must be included with all submissions.

Clinical trial registration	The trial is registered at the EU Clinical Trial database (EudraCT number 2016-003678-42) and was approved by the Medical Research Ethics Committees United (MEC-U, NL60807.100.17-R17.039) in the Netherlands and the South Central - Hampshire B Research Ethics Committee (REC, 19/SC/0368) in the UK.
Study protocol	The full trial protocol can be sent upon request to the corresponding author. The brief protocol is available at https://www.clinicaltrialsregister.eu/ctr-search/trial/2016-003678-42/results (EudraCT number 2016-003678-42)
Data collection	Clinical data of participants was recorded using OpenClinica, electronic case record form software that enables compliance with regulatory guidelines such as 21 CFR Part 11. The same online database system was used across the Dutch and UK sites. In the Netherlands, participants/parents/legal guardians were asked to keep their vaccination booklets at hand for the first visit. In case participants/parents/legal guardians did not have a vaccination booklet anymore, permission was asked to contact DVP (Vaccine Supply and Prevention Programs Service) to check their vaccination status according to NIP. In the UK, vaccination history was obtained either from the child's 'Red Book', or alternatively by means of the participant seeking confirmation from the GP recorded on a standard document.
Outcomes	The primary outcome was pertussis toxin-specific IgG antibody concentration at D28 post-vaccination. Secondary outcomes include but are not limited to IgG responses against the other Tdap vaccine antigens TT, Dt, Prn, FHA at D0, D28, and Y1, and PT at D0 and Y1. Exploratory outcomes relevant for this manuscript were measured at D0 and D1 post vaccination, whole blood transcriptomic analysis, measurements of immune cell abundance and cytokine production by mass cytometry, and single-cell gene expression profiling of innate immune cells.

Plants

Seed stocks	NA
Novel plant genotypes	NA
Authentication	NA

Flow Cytometry

Plots

Confirm that:

- [x] The axis labels state the marker and fluorochrome used (e.g. CD4-FITC).
- [x] The axis scales are clearly visible. Include numbers along axes only for bottom left plot of group (a 'group' is an analysis of identical markers).
- [x] All plots are contour plots with outliers or pseudocolor plots.
- [x] A numerical value for number of cells or percentage (with statistics) is provided.

Methodology

Sample preparation	In order to isolate cells for single-cell RNA sequencing, up to 4ml of whole blood was treated with in-house prepared ammonium-chloride-potassium red blood cell lysis buffer for 15 minutes at RT. Cells were washed with PBS and stained with a solution of PBS + 2mM EDTA (hereafter referred to as PBS-EDTA) containing fixable viability dye (FVD) blue (Thermo Fisher) at RT, and then washed again. Cells were then stained with a mixture of fluorescently labelled antibodies (Table 6 of the manuscript) for 30 minutes at RT, washed with PBS-EDTA, resuspended in FACS buffer (PBS + 0.2% bovine serum albumin + 0.09% sodium azide) with 2mM EDTA (Sigma), filtered through a 30µm pore size mesh cell strainer (Sysmex) and kept on ice.
Instrument	FACS Aria II cell sorter (BD Bioscience)
Software	FACSDiva software version 7 (BD Bioscience)
Cell population abundance	Single-cell sorting was performed into 384-well plates.
Gating strategy	The boolean gating strategy is available in Figure S13A. All gates starting from the raw data until the final single-cell sorted population are shown. Boundaries of negative and positive cell populations are shown for each marker.

☒ Tick this box to confirm that a figure exemplifying the gating strategy is provided in the Supplementary Information.
